# Pharmacokinetic–pharmacodynamic cutoff values for benzylpenicillin in horses to support the establishment of clinical breakpoints for benzylpenicillin antimicrobial susceptibility testing in horses

**DOI:** 10.3389/fmicb.2023.1282949

**Published:** 2023-10-25

**Authors:** Elodie A. Lallemand, Alain Bousquet-Mélou, Laura Chapuis, Jennifer Davis, Aude A. Ferran, Butch Kukanich, Taisuke Kuroda, Marlène Z. Lacroix, Yohei Minamijima, Lena Olsén, Ludovic Pelligand, Felipe Ramon Portugal, Béatrice B. Roques, Elizabeth M. Santschi, Katherine E. Wilson, Pierre-Louis Toutain

**Affiliations:** ^1^INTHERES, Université de Toulouse, INRAE, ENVT, Toulouse, France; ^2^Department of Biomedical Sciences and Pathobiology, Virginia-Maryland College of Veterinary Medicine, Blacksburg, VA, United States; ^3^Department of Anatomy and Physiology, Kansas State University, Manhattan, KS, United States; ^4^Clinical Veterinary Medicine Division, Equine Research Institute, Japan Racing Association, Shimotsuke, Japan; ^5^Drug Analysis Department, Laboratory of Racing Chemistry, Utsunomiya, Japan; ^6^Division of Pharmacology and Toxicology, Department of Clinical Sciences, Swedish University of Agricultural Sciences, Uppsala, Sweden; ^7^Department of Comparative Biomedical Sciences, The Royal Veterinary College, University of London, London, United Kingdom; ^8^Department of Clinical Sciences, Kansas State University, Manhattan, KS, United States

**Keywords:** benzylpenicillin, horse, antimicrobial susceptibility testing, PK/PD cutoff, population pharmacokinetics, Monte Carlo simulations, probability of target attainment

## Abstract

**Introduction:**

The aim of this international project was to establish a species-specific Clinical Breakpoint for interpretation of Antimicrobial Susceptibility Testing of benzylpenicillin (BP) in horses.

**Methods:**

A population pharmacokinetic model of BP disposition was developed to compute PK/PD cutoff values of BP for different formulations that are commonly used in equine medicine around the world (France, Sweden, USA and Japan). Investigated substances were potassium BP, sodium BP, procaine BP, a combination of procaine BP and benzathine BP and penethamate, a prodrug of BP. Data were collected from 40 horses that provided 63 rich profiles of BP corresponding to a total of 1022 individual BP plasma concentrations.

**Results:**

A 3-compartment disposition model was selected. For each of these formulations, the PK/PD cutoff was estimated for different dosage regimens using Monte Carlo simulations. The *f*AUC/MIC or *f*T>MIC were calculated with a free BP fraction set at 0.4. For *f*AUC/MIC, a target value of 72 h (for a 72h treatment) was considered. For *f*T>MIC, efficacy was assumed when free plasma concentrations were above the explored MIC (0.0625-2 mg/L) for 30 or 40 % of the dosing interval. For continuous infusion, a *f*T>MIC of 90 % was considered. It was shown that a PK/PD cutoff of 0.25 mg/L can be achieved in 90 % of horses with routine regimen (typically 22,000 IU/kg or 12.4 mg/kg per day) with IM procaine BP once a day (France, Japan, Sweden but not USA1) and with IM sodium BP at 14.07 mg/kg, twice a day or IV sodium BP infusion of 12.4 mg/kg per day. In contrast, penethamate and the combination of procaine BP and benzathine BP were unable to achieve this PK/PD cutoff not even an MIC of 0.125 mg/L.

**Discussion:**

The PK/PD cutoff of 0.25 mg/L is one dilution lower than the clinical breakpoint released by the CLSI (0.5 mg/ L). From our simulations, the CLSI clinical breakpoint can be achieved with IM procaine BP twice a day at 22,000 IU i.e. 12.4 mg/kg.

## 1. Introduction

Benzylpenicillin (BP), including procaine BP, sodium BP, potassium BP, benzathine BP, and penethamate hydriodide, is reported to be the most frequently used beta-lactam in equine medicine in the UK (Hughes et al., 2013) and the EU (De Briyne et al., 2014). Because BP has a narrow spectrum with a lower risk of selection for resistant bacteria, the veterinary committee of the European Medicines Agency (EMA/CVMP) has recommended that BP should be used for first-line treatment wherever possible (Anonymous, 2015). This is the case in horses, where it is used to treat various conditions associated with gram-positive pathogens such as *Streptococcus* spp. The emergence of multi-drug-resistant bacteria is a global issue in horses, as in other species (Weese et al., 2008). Beta-hemolytic *Streptococci* are almost always susceptible to BP, with a reported MIC_90_ ≤ 0.06 μg/mL (Younkin et al., 2019). On the other hand, resistance to *Staphylococcus aureus* (SA) is reported in horses; furthermore, although the prevalence of methicillin-resistant SA (MRSA) remains low (Maddox et al., 2012; Choi et al., 2018), the susceptibility of SA to BP is unpredictable, and *in vitro* antimicrobial susceptibility testing (AST) is required to distinguish methicillin-susceptible SA (MSSA) from MRSA (Weese et al., 2008). The interpretation of AST requires the establishment of clinical breakpoints (CBs) to enable qualitative reporting of the results of AST as Susceptible (S), Intermediate (I) or Resistant (R). The Clinical and Laboratory Standards Institute (CLSI), with its Subcommittee on Veterinary Antimicrobial Susceptibility Testing (VAST), is the only organization that has proposed CBs for BP in horses. For both *Streptococcus* spp. and SA, the VAST/CLSI CBs are ≤0.5, 1, and ≥ 2 μg/mL for S, I, and R, respectively (Wayne, 2018). These CBs were derived from data obtained using a procaine BP formulation at an intramuscular (IM) dose of 22,000 IU/kg every 24 h (Wayne, 2018). CBs are dose- and formulation-dependent, and there is no reason to assume that these VAST/CLSI CBs are also valid for any other BP formulations or dosage regimens. Hence, the objectives of this project have been to document the pharmacokinetics (PK) of the different formulations and modalities of administration of BP as they are used in four different countries (France, Sweden, Japan, and the United States) and, if required, to revisit and/or extend the current CBs according to the VetCAST approach (Toutain et al., 2017).

VetCAST is the veterinary sub-committee of the European Committee on Antimicrobial Susceptibility Testing (EUCAST), the reference committee for AST in human medicine for the EU (Toutain et al., 2017). According to VetCAST, CBs are determined by considering at least an epidemiological cutoff (ECOFF) and a PK/PD cutoff (PK/PD_CO_). The PK/PD_CO_ is defined as the highest possible MIC at which a given percentage of animals in the target population (most often 90%) achieve a predefined pharmacodynamic target (PDT) value. In addition, a clinical cutoff can also be considered when clinical data are available to link MICs to clinical efficacy (Turnidge and Martinez, 2017), but such data do not exist for BP in horses. A robust estimation of PK/PD_CO_, which is pivotal for the VetCAST approach, first requires the construction of a valid population PK model from individual animal data collected from a variety of sources in order to quantify typical values of PK parameters and their between-subject variability (BSV). Subsequently, this population PK model is used to generate, *via* Monte Carlo simulations, large meta-populations of PK profiles for different dosage scenarios based on the different types of formulations and methods of administration used for BP treatment in horses. From these *in silico* profiles, the values of the PK/PD indices can be computed and ultimately used to determine the highest MIC value for which the predefined PDT is reached in 90% of horses (Toutain et al., 2017b). BP is a time-dependent antimicrobial, and the PK/PD index selected to predict its efficacy is usually the time (T) that the free (*f*) plasma concentration of BP exceeds the MIC of the pathogen (*f*T > MIC). In horses, the mean plasma protein binding of BP has been reported to be 62.8 ± 1.8% (Olsén et al., 2013). Using bioassay or spectrophotometry, similar values of 57.3 ± 7.3% (Firth et al., 1986) and from 46 to 48% (Dürr, 1976) have been reported. The PDT to achieve for *f*T > MIC is typically 30–40% of the dosing interval, i.e., when BP is administered repeatedly (e.g., q6h) as aqueous potassium or sodium BP solution by intravenous (IV) or IM routes. For long-acting (LA) formulations, the *f*T > MIC criterion may pose difficulties with the occurrence of a jump discontinuity (Toutain et al., 2021), and the *f*AUC/MIC PK/PD index (with AUC being the area under the plasma concentration curve) is considered more stable (Toutain et al., 2021). In addition, it has been shown for beta-lactams that the *f*AUC/MIC index is the most suitable index when the half-life of the drug is rather long (Nielsen et al., 2011), as is the case with LA formulations of BP. For the present analysis, both PK/PD indices were investigated.

In equine medicine, BP can be administered using a variety of formulations (solution and suspensions) and different routes of administration (IV for solution and IM for both solution and suspensions). The most commonly administered suspension in horses is procaine BP. Procaine BP is inexpensive and convenient, requiring less frequent dosing (e.g., q12–24 h) than for solutions (q4–6h), but the result is lower and more variable plasma concentrations (Love et al., 1983), depending on the route of administration (Firth et al., 1986). More importantly, procaine BP can also result in severe adverse effects, including central nervous system excitement, seizures, and even death caused by inadvertent intravascular injection of procaine (Nielsen et al., 1988; Tjälve, 1997; Olsén et al., 2007). In addition, in some jurisdictions, procaine BP is a prohibited drug in racehorses (Kuroda et al., 2021) due to the persistence of procaine, a doping substance (Tobin and Blake, 1976). Several LA formulations exist as alternatives to procaine BP for use in horses, including benzathine BP and penethamate. Benzathine BP is formulated from two BP molecules reacting with diphenylethylene diamine (Gartlan et al., 2021). Penethamate is the diethylaminoethyl ester of BP. Penethamate is a prodrug of BP and is slowly absorbed into the circulation, then hydrolyzed to BP (Love et al., 1983). In formulations intended for veterinary use, the compound is incorporated as the hydriodide (Anonymous, 1995). Penethamate is approved in the EU for horses (Anonymous, 1995), but it is not approved by the FDA for horses in the USA (Knych and Magdesian, 2021). To the best of our knowledge, no PK data have been published on the disposition of BP after administration of penethamate in horses. Based on data already published or specifically generated for this project, our goal was to document the PK of the different BP formulations used in equine medicine and to calculate, for each of them, the PK/PD cutoff corresponding to the different dosing scenarios with which they are or can be prescribed. See Table 1 for definitions of the main terms, abbreviations, and acronyms used in this paper.

**Table 1:** Definition of the main terms, abbreviations, and acronyms used in this paper.

Antimicrobial susceptibility testing (AST)
Area under the plasma concentration curve (AUC)
Benzylpenicillin (BP)
Bayesian Information Criterion (BIC)
Below the limit of quantification (BLOQ)
Between-subject variability (BSV)
Clinical breakpoint (CB)
Clearance (CL)
Clinical and Laboratory Standards Institute (CLSI)
Continuous-rate infusion (CRI)
Coefficient of variation (CV)
Committee for Veterinary Medicinal Products (CVMP)
Epidemiological cutoff (ECOFF)
European Medicines Agency (EMA)
European Committee on Antimicrobial Susceptibility Testing (EUCAST)
Free (*f*)
Bioavailability (F)
Ratio of the area under the plasma concentration time curve for free drug to minimum inhibitory concentration (*f*AUC/MIC)
Time that the free plasma concentration of the antibiotic exceeds the MIC of the pathogen (*f*T > MIC)
Intermediate (I)
Intramuscular (IM)
International units (IU)
Intravenous (IV)
First-order rate constants of absorption (Ka)
Long-acting (LA)
Liquid chromatography coupled to tandem mass spectrometry (LC–MS/MS)
Limits of quantification (LOQ)
Mean absorption time (MAT)
Minimum inhibitory concentration (MIC)
Methicillin-resistant *Staphylococcus aureus* (MRSA)
Mean residence time (MRT)
Methicillin-susceptible *Staphylococcus aureus* (MSSA)
Non-compartmental analysis (NCA)
Non-linear mixed-effects model (NLMEM)
Pharmacodynamic (PD)
Pharmacodynamic target value (PDT)
Pharmacokinetic (PK)
Pharmacokinetic/pharmacodynamic cutoff (PK/PDco)
Probability of target attainment (PTA)
Quantiles (Q%)
Resistant (R)
Susceptible (S)
*Staphylococcus aureus* (SA)
Summary of product characteristics (SPC)
Time (T)
Lag time (Tlag)
Volume(s) of distribution (V)
Subcommittee on Veterinary Antimicrobial Susceptibility Testing (VAST)
EUCAST Veterinary Subcommittee on Antimicrobial Susceptibility Testing (VetCAST)
Visual predictive check (VPC)
Steady-state volume of distribution (Vss)

## 2. Materials and methods

### 2.1. Raw data collection

Raw data collected in four countries (France, Sweden, the USA, and Japan) were considered. Some of the datasets USA1 (Younkin et al., 2019), USA2 (Wilson et al., 2022), and Sweden (Olsén et al., 2013, 2018) have previously been published, but the French data were specifically generated for this project. The Japanese data have not been published. All raw data are available in a Supplementary Excel file.

The doses reported in the corresponding publications or in the Summary of Product Characteristics (SPC) of the formulations were presented in terms of either international units (IU) or mass (mg/kg). For the present meta-analysis, all the doses were harmonized in terms of BP (mg/kg), which required the use of different conversion factors (Supplementary Table 1). Doses were expressed in terms of BP according to the second international standard for BP, i.e., considering 1 mg of sodium BP to be equivalent to 1,670 IU or 1 mg of BP equivalent to 1780 IU. Details on registered name, dosage, and conversion factor for each of the different formulations are given in Supplementary Tables 1, 2.

Data from horses (*n* = 40) were considered for the present analysis; these corresponded to different trials conducted in France (*n* = 6), in Sweden (*n* = 15), in Japan (*n* = 6), and in the USA (USA1, *n* = 7; USA2, *n* = 6). In Japan, horses received a single IM dose of procaine BP. In USA1, seven horses received two IV administrations of potassium BP, followed by a single dose of procaine BP (Younkin et al., 2019). In USA2, six horses received a single IV administration of potassium BP in association with gentamicin (Wilson et al., 2022). In Sweden, four horses received a single IV dose of sodium BP (Olsén et al., 2018) and eight other horses were enrolled in a 2 × 2 crossover design aiming to compare a series of four IM administrations of procaine BP and a series of seven IM administrations of sodium BP at 12 h intervals (Olsén et al., 2013). In France, six horses were first enrolled in a 3 × 3 crossover design to compare three formulations: procaine BP (three IM administrations at 24 h intervals), an LA formulation consisting of a mixture of procaine BP and benzathine BP (two IM administrations with a 48 h interval), and penethamate hydriodide (three IM administrations at 24 h intervals). After completion of the 3 × 3 crossover and a washout period of 39 weeks, the same six horses received a single IV administration of sodium BP. A total of 63 rich profiles of BP plasma concentration were available for the present meta-analysis, corresponding to a total of 1,022 individual BP plasma concentration measurements. Details on the different experimental designs are given in Supplementary Table 2, and the demographics of the horses used (body weight, age, sex, breed, etc.) are given in Supplementary Table 3. All BP plasma concentrations were measured by liquid chromatography coupled to tandem mass spectrometry (LC–MS/MS). The limits of quantification (LOQ) were 5.5 ng/mL for Sweden, 10 ng/mL for France and USA1, 30 ng/mL for Japan, and 40 ng/mL for USA2. Details of the French and Japanese analytical methods that have not been published previously are given in Supplementary Data 1.

**Figure 1:**
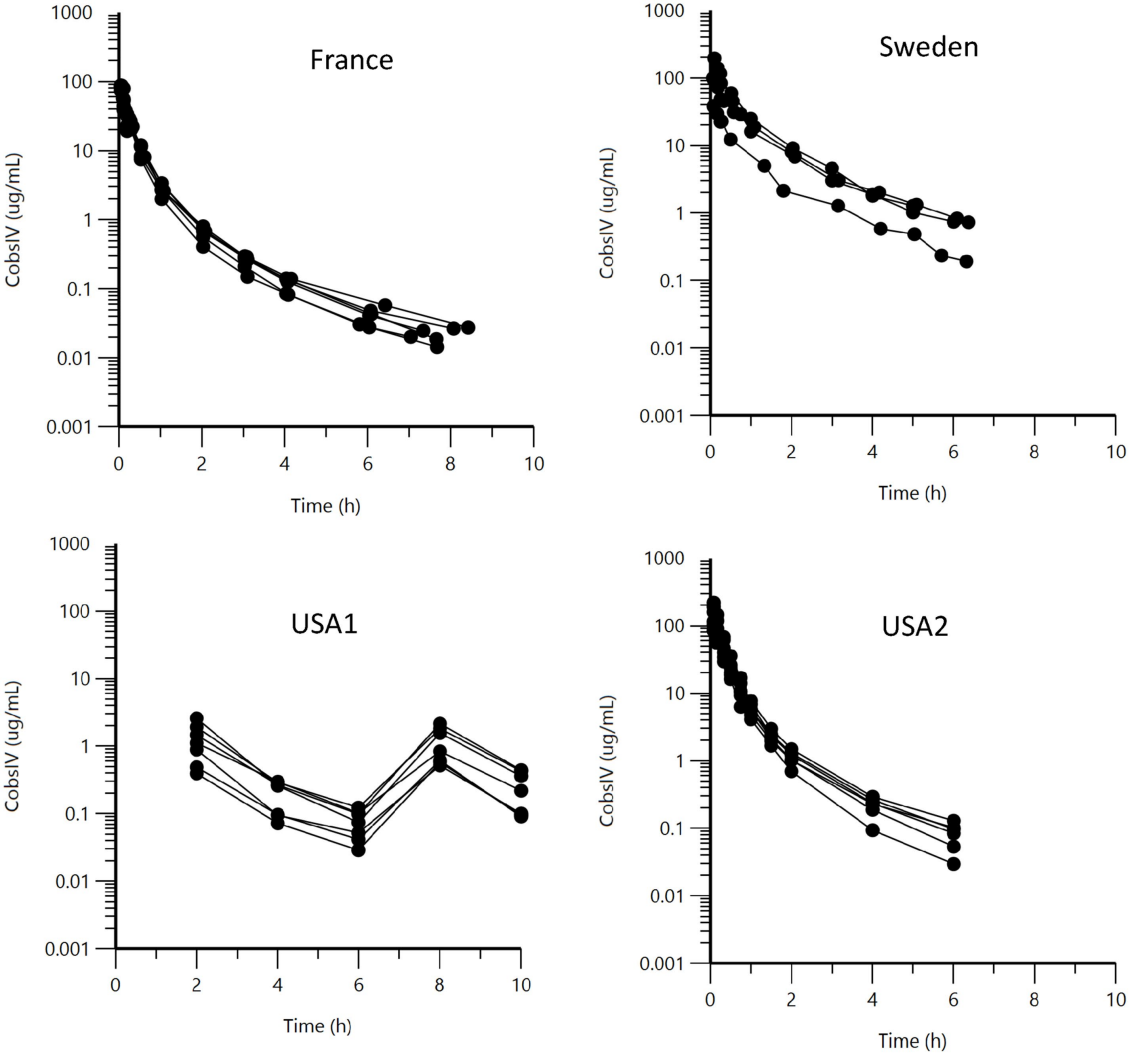
Semi-logarithmic spaghetti plots of the plasma concentration (μg/mL) vs. time (h) disposition curves for BP after IV administration of sodium or potassium BP at 12.36 mg/kg (22,000 IU/kg) in six French horses, six USA2 horses (in association with gentamicin, 6.6 mg/kg), and seven USA1 horses. For the four Swedish horses, doses ranged from 16.44 to 25.11 mg/kg.

**Figure 2:**
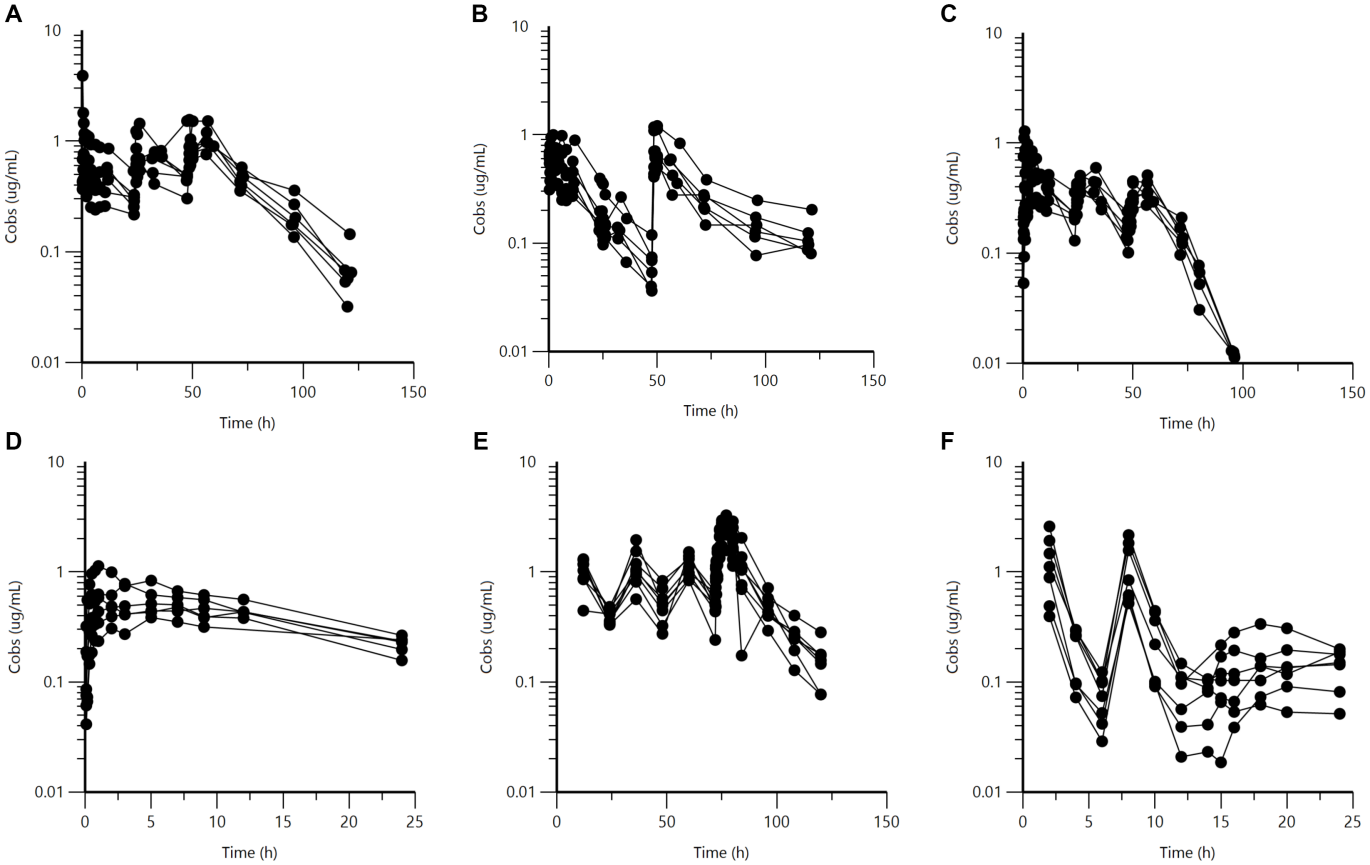
Semi-logarithmic spaghetti plots of the plasma concentration (μg/mL) vs. time (h) disposition curves for BP after IM administration of different long-acting BP formulations. Upper panel: French horses (*n* = 6) administered **(A)** procaine BP, **(B)** a combination of procaine and benzathine BP, and **(C)** penethamate. Dosing (BP) consisted of three doses of 10 mg/kg at 24 h intervals for procaine BP, and 12.4 mg/kg twice with a 48-h interval for the combination of procaine and benzathine BP. For penethamate, the SPC gives a fixed dose independent of the body weight of the horse, namely, 7.72 g of penethamate or 5.96 g of BP on the first day and 3.86 g of penethamate or 2.98 g of BP per horse on the second and third day of treatment. This corresponds to a BP dose of 11.91 mg/kg on Day 1, followed by 5.96 mg/kg on Day 2 and Day 3 for a typical 500 kg BW horse. Lower panel: procaine BP for **(D)** Japanese, **(E)** Swedish, and **(F)** USA1 horses. For Japanese horses (*n* = 6), the single dose was 5.617 mg/kg. For Swedish horses (*n* = 8), four doses of approximately 11.80 mg/kg were administered at 24-h intervals, and for USA1 horses (*n* = 7), procaine BP (12.36 mg/kg) was administered once 6 h after the second of two IV administrations of potassium BP (12.36 mg/kg) with a 6-h interval.

**Figure 3:**
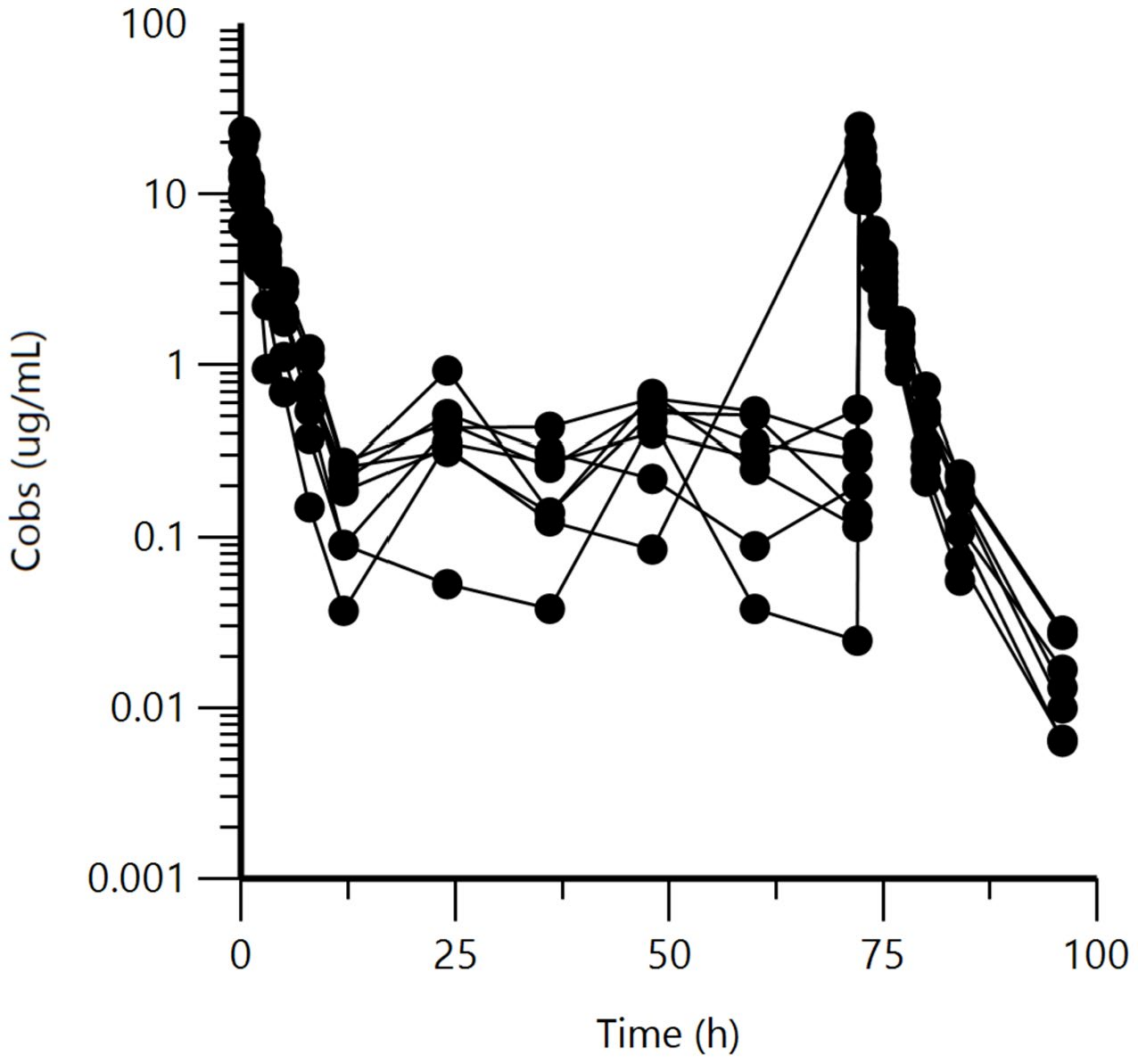
Semi-logarithmic spaghetti plots of the plasma concentration (μg/mL) vs. time (h) disposition curves for BP after IM administration of sodium BP at dose of approximately 14.07 mg/kg, seven times at 12-h intervals, to eight Swedish horses. Rich samplings were only performed for the first and the last administrations.

Raw data are presented as a series of spaghetti plots in Figures 1–3. All doses are expressed in terms of BP (mg/kg).

Visual inspection of Figure 2 indicates that USA1 BP plasma concentrations after procaine BP (panel F) were significantly lower than those from the other three datasets (French, Swedish, and Japanese) for procaine BP, which did not appear to vary among themselves in any major way. The slope of procaine BP for USA1 horses was rather flat.

Other spaghetti plots comparing the different formulations with or without scaling for the administered doses are given in Supplementary Figures 1–3. They show consistency in the IV data and differences between LA formulations of BP.

### 2.2. Data modeling

Data analysis was carried out using Phoenix^®^WinNonlin^®^8.3, Certara, Princeton, New Jersey, USA. The first step was to perform a non-compartmental analysis (NCA) to obtain an initial estimate of basic pharmacokinetic parameters, such as plasma clearance and volumes of distribution from the four IV data sets. The AUC for each profile was also estimated to assess relative bioavailability under the different modalities of administration and formulations, using the French IV data as the reference. These parameters were used as initial starting values for the comprehensive population PK model that was developed using a non-linear mixed-effects model (NLMEM).

All raw data were considered, but concentrations reported as below the limit of quantification (BLOQ; less than 5% of concentration measurements) were ignored, without introducing a risk of leading to any structural model misspecification (Byon et al., 2008). The 23 IV data profiles were modeled first using a 2- or a 3-compartment model to identify the structural model that best described the BP disposition. The two models were compared using the likelihood ratio test and the 3-compartment model was selected.

Parameterization of this IV model was in terms of plasma clearance (CL), inter-compartmental clearance(s) (CL2 and CL3), and volume(s) of distribution (V), with Vc, V2, V3, CL, CL2, and CL3 being the primary estimated parameters.

Between-subject variability (BSV) was modeled using an exponential model of the form:


(1) $$\theta_{\text{clearance}:i}=\theta_{tv:\text{clearance}}\times \exp\left(\eta_{i}\right)$$

where *θ_clearance_i_* is the value of theta (here, plasma clearance) in the *i*th horse, $\theta_{tv:\text{clearance}}$ is the typical population value of this theta variable (Vc, V2, V3, CL, CL2, CL3), and $\eta_{i}$ is the deviation (denoted by eta) from the corresponding population theta value associated with the *i*th horse. The distribution of eta values was assumed to be normal, with a mean of 0 and variance ω^2^. Each of the eta distributions associated with each theta variable had its own variance, which was estimated. For the present analysis of the IV data, a full OMEGA matrix was considered, meaning that both variance and covariance terms were estimated. BSV was estimated from ω^2^ (variance) using equation (2), which converts variance terms in the form of coefficients of variation (CV%) back onto the original scale.


(2) $$CV\left(\%\right)=100\times \sqrt{\exp\left(\omega^{2}\right)-1}$$

To compute PK/PD cutoff values corresponding to selected scenarios (in terms of route of administration, formulation, and dosage regimen) that encompass the entire population of horses, no covariate (such as breed, age, sex, etc.) should be considered. However, in the present analysis, we documented the “source of dataset” as a categorical covariate in order to assess the comparability of the four IV data sets and to compute bioavailability, taking into account the fact that some horses received both IV administration of BP and one (USA1) or more (French) IM LA formulations. The selected covariate model was an exponential model for the six structural parameters of the IV model. The Phoenix covariate Search Shotgun run mode was used to assess the significance of the effect of the covariate on these different PK parameters. The 64 scenarios were ranked based on the Bayesian Information Criterion (BIC) (Burnham and Anderson, 2004). In addition, the BIC was evaluated to assess the significance of adding or not adding a covariate. A difference of >10 in BIC between models is accepted as “very strong” evidence in favor of the model with the lower BIC (Kass and Raftery, 1995). For the present analysis, the BIC was 241.6 for the model without the covariate and 208.7 for the best model that included the covariate “source of dataset” only in the case of plasma clearance. This last IV model was selected as the best one to use for subsequent simple runs.

**Figure 4:**
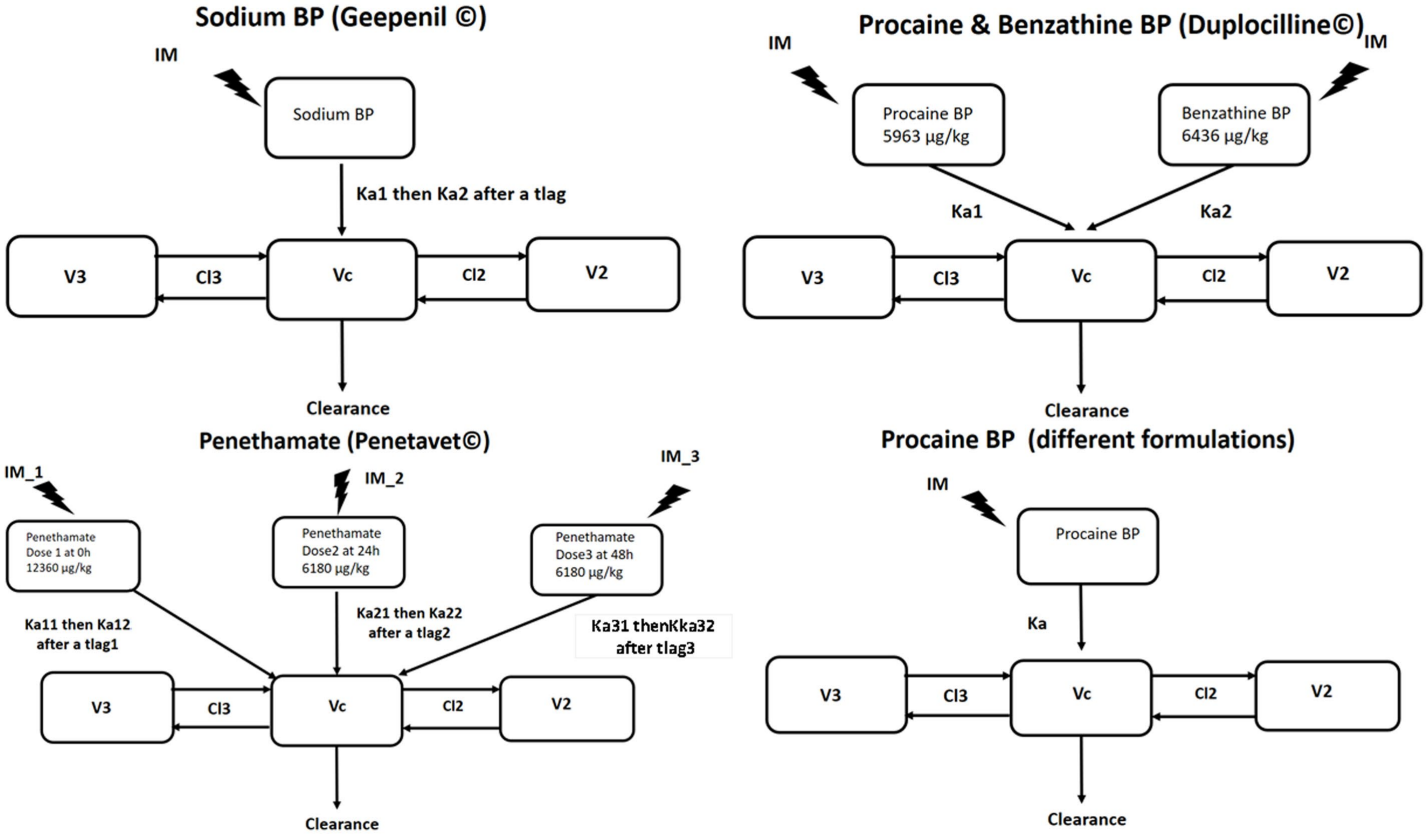
The 3-compartment model selected for analysis of BP plasma concentration after IM administration of sodium BP, procaine BP, a combination of procaine BP and benzathine BP, and penethamate. The first-order absorption process was described by a single rate constant (Ka) for procaine BP; by two sequential rate constants (Ka1 then Ka2) separated by a lag time for sodium BP; by two parallel rate constants (Ka1 and Ka2) for the procaine/benzathine combination; or by two sequential rate constants (Ka1 then Ka2) separated by a lag time for each administration site for penethamate. Vc, V2, V3, CL, CL2, and CL3 are the shared disposition parameters of BP for IV and IM administration.

In a second step, each IM formulation was analyzed separately using the above-described consolidated IV 3-compartment model to determine the best structural model to describe the absorption process and to obtain initial parameter values (first-order rate constants for absorption Ka and bioavailability F) for the final model. The absorption process was described by a single rate constant for procaine BP. For sodium BP, two sequential rate constants of absorption separated by a lag time (Tlag) were required to describe biphasic absorption, with a rapid initial phase followed by a second slower phase of absorption. For the fixed combination of procaine BP and benzathine BP, the total administered dose of BP was split into two fractions in proportion to the composition indicated by the SPC. For this combination, the plasma concentration measurements corresponded to the sum of the concentrations of BP coming from each of the ingredients, and the model consisted of two sets of equations corresponding, respectively, to each of the two administered prodrug ingredients, each with its own absorption rate constant (both in parallel) and its own bioavailability. For BP plasma concentration measurements taken after a series of three IM administrations of penethamate at 24 h intervals, it was necessary to use two sequential rate constants of absorption (rapid then slow) for each site of administration (*n* = 3), separated by a lag time. Figure 4 depicts these different structural models for the IM formulations.

For all IM formulations, bioavailability (*F*) was estimated using an ilogit transformation (equation 3), preventing the possibility of estimating *F* > 1.


(3) $$F=\frac{\exp\left(\text{ilogitF}\right)}{1+\exp\left(\text{ilogitF}\right)}$$

For this preliminary data analysis of each IM data set, the IV fixed parameters (theta values) were set (frozen) to the optimal values identified in the analysis of IV data alone, but OMEGA, which now included the BSV of parameters associated with the process of absorption (Ka and F), were allowed to be optimized.

Finally, all data were modeled simultaneously. The motivation for this global analysis was that the data collected for these meta-analyses were very unbalanced (e.g., no IV data were available for Japan; only IV data were available in the USA2 dataset; LOQ varied). By simultaneously analyzing all the collected data, it was possible to estimate plasma clearance for horses not investigated IV, as in the case of Japanese and Swedish horses treated with procaine BP (see later). The second motivation was to assess whether it was reasonable for the Monte Carlo simulations to group together certain formulations administered IM to generate a common PK/PD cutoff value with shared parameters, or whether, instead, it was more realistic to compute different PK/PD cutoff values for each formulation.

Given the size of the full model and the number of parameters to be estimated, the IV disposition parameters (CL, CL2, CL3, Vc, V2, and V3) were frozen at their optimal values obtained from the analysis of IV data, including the covariate “source of dataset,” for clearance values. However, all the other parameters of the model and OMEGA were re-estimated. In addition, for clearance, the covariate “source of dataset” was introduced into the model for the 8 Swedish horses having received procaine BP and sodium BP IM (no IV route for these 8 horses investigated under a crossover 2 × 2 design) and for the Japanese horses that only received procaine BP. For the 8 Swedish horses, the justification for this covariate was that the estimated bioavailability computed using the clearance of the other 4 Swedish horses having received IV (without IM) was very low (37%). There was no reason to assume that the 8 Swedish horses having received IM administration of procaine and sodium BP had the same clearance as the other 4 Swedish horses having received an IV administration of sodium BP. The introduction of the covariate “source of dataset” for the clearance of Swedish horses made it possible to calculate an estimated bioavailability of procaine BP for Swedish horses that was similar to that of other horses (French, USA1, and Japanese). For French and USA1 horses, bioavailability was estimated using the actual measured plasma clearance for these horses.

For all modeling, the residual model was an additive plus multiplicative (proportional) model.

The corresponding Phoenix script for the final model is given in Supplementary Data 2.

Parameters and their associated SE (as a measure of the precision of the estimation) were estimated using the quasi-random parametric expectation maximization engine to fit the IV data, and then using FOCE ELS for all other runs. The Jacobian SEs could not always be obtained, and a bootstrap method was used when possible. When the computation time was prohibitive, as for the adjustment of the full model (which required approximately 1 h for each iteration), no SEs were generated because this would have required several weeks of computation time.

Several parameters were computed as secondary parameters, namely, the steady-state volume of distribution (Vss, with Vss being the sum of Vc, V2, and V3); the mean residence time (MRT), as the ratio of Vss to clearance; and the terminal half-life, computed from clearance and volume terms according to equations given by Dubois et al. (2011). The mean absorption time (MAT) for the different rate constants of absorption (Ka) was computed as 1/Ka.

### 2.3. PK/PD integration and Monte Carlo simulations

The goal of this data analysis was to compute the different PK/PD cutoff values corresponding to the different routes of BP administration (IV vs. IM), formulations, and dosage regimens. For the present project, two PK/PD indices were compared: first, the classical index for beta-lactam antibiotics,% *f*T > MIC; and additionally, *f*AUC/MIC, which can be used as a relevant index for any LA formulation, regardless of the antimicrobials investigated (Nielsen et al., 2011). The italic *f* indicates that the free concentration of BP must be considered in order to compute a PK/PD cutoff value. The extent of BP plasma protein binding was evaluated in Swedish horses at 62.8% (Olsén et al., 2013). For our simulations, *f_u_* was introduced into the model, with a typical value of 0.4. For the index % *f*T > MIC, the percentage of the dosage interval considered to achieve efficacy was 30 or 40% (Toutain et al., 2021). For the index *f*AUC/MIC, results are reported in hours; an *f*AUC/MIC of 72 h over a period of 72 h corresponds to an average free plasma concentration of BP equal to the MIC over the treatment duration (Toutain et al., 2007). For both indices, MICs of 0.0625, 0.125, 0.25, 0.375, 0.5, 1, and 2 mg/L were explored. A variety of IV and IM dosages and dosing regimens were simulated; these are given in Supplementary Table 4. These reflect modalities of administration (IV bolus, IV infusion, IM) and dosage regimens that are used around the world, depending on the pathogen to be eradicated, prescriber opinion, or the SPC of marketed formulations. The final models were used for these simulations. For procaine BP, PK/PD cutoff values were computed considering the clearance covariate for France, Sweden, USA1, and Japan. Using Monte Carlo simulations, 5,000 curves were generated for each explored scenario, and metrics of interest (*f*AUC/MIC and %Time > *f*MIC) were computed directly using appropriate coding in Phoenix (see Supplementary Data 2). These vectors of 5,000 values were then processed using the relevant statistical tool in Phoenix to compute different statistics: specifically, the different quantiles (Q%) of interest, with Q10% of the distribution corresponding to the 90% quantile of interest. The Q10% value enables determination of the PK/PD cutoff value, i.e., the highest MIC for which 90% of horses would be able to achieve the predetermined PDT of the PK/PD index. To construct plots of probability of target attainment (PTA) vs. MIC, the Phoenix statistical tool was used to calculate all quantiles (from 1 to 99) of the variable of interest. These variables were *f*AUC/MIC and time (h) above the fixed PDT. For Time > *f*MIC, a PDT of 30% or 40% of the dosing interval corresponds to 21.6 h and 28.8 h, respectively, for a treatment of 72 h duration. After exporting the results table to Excel, we identified the PTAs for these PDTs.

## 3. Results

### 3.1. Non-compartmental analysis

IV data from the France (*n* = 6), Sweden (*n* = 4), and USA2 (*n* = 6) datasets were analyzed using an NCA approach to obtain an initial estimate of basic pharmacokinetic parameters such as plasma clearance and volumes of distribution. The USA1 dataset was not analyzable *via* an NCA approach given that only 3 sampling points were obtained. Table 2 presents the IV results.

**Table 2:** Results of the NCA analysis (Model 200–202, linear trapezoidal rule) for IV data in 16 horses (French, Swedish, and USA2).

Variable	Units	Source	Mean	SD	CV (%)	Min	Median	Max	Harmonic Mean
Clearance	mL/h/kg	France	564	69	12.31	486	559	676	
Clearance	mL/h/kg	Sweden	423	363	85.78	162	289	953	
Clearance	mL/h/kg	USA2	300	95	31.62	193	289	453	
Cmax	ug/mL	France	79	12	15.06	57	85	88	
Cmax	ug/mL	Sweden	117	66	56.10	38	118	195	
Cmax	ug/mL	USA2	145	53	36.75	84	137	220	
HL_Lambda_z	h	France	1.49	0.23	15.36	1.24	1.40	1.83	1.46
HL_Lambda_z	h	Sweden	1.28	0.26	20.63	0.91	1.35	1.50	1.23
HL_Lambda_z	h	USA2	0.955	0.095	9.98	0.787	0.962	1.072	0.947
MRTINF	h	France	0.478	0.061	12.74	0.415	0.466	0.585	
MRTINF	h	Sweden	1.03	0.15	14.93	0.895	1.000	1.239	
MRTINF	h	USA2	0.45	0.04	8.62	0.384	0.456	0.498	
Vz	mL/kg	France	1,216	255	20.93	872	1,248	1,584	
Vz	mL/kg	Sweden	792	665	83.89	213	615	1727	
Vz	mL/kg	USA2	403	86	21.32	298	401	515	
AUCINF	h*ug/mL	France	22.0	2.5	11.49	18.2	22.2	24.9	
AUCINF	h*ug/mL	Sweden	70.4	33.3	47.30	24.5	77.7	101.8	
AUCINF	h*ug/mL	USA2	44.6	13.6	30.47	27.3	42.9	64.2	

AUCINF: Area under the curve extrapolated to infinity; Cmax: maximal initial plasma concentration; HL_lambda_z: terminal half-life; MRTINF: mean residence time extrapolated to infinity; Vz: volume of distribution of the terminal phase.

Table 3 gives the observed AUCs for all tested formulations. For comparability, all AUCs were scaled to a typical dose of 12.36 mg/kg (22,000 IU per kg).

**Table 3:** Observed AUC (0–tlast) (μg*h/mL) scaled to a typical dose of 12.36 mg/kg (22,000 IU/kg) for the 40 horses and 63 profiles.

Product	Horses (N)	Country	Mean	SD	CV Percent	Min	Max
Procaine BP	6	France	24.39	6.41	26.27	17.67	36.15
Procaine BP + benzathine BP	6	France	16.49	6.40	38.81	12.20	29.17
Penethamate	6	France	12.96	1.24	9.60	11.43	14.45
Procaine BP	6	Japan	21.38	5.21	24.37	15.32	30.46
Potassium BP + procaine BP	7	USA1	2.50	1.29	51.49	1.03	4.26
Sodium BP IV	4	Sweden	44.33	27.06	61.04	12.79	75.25
Sodium BP IV	6	France	22.12	2.63	11.88	18.26	25.39
Potassium BP IV	6	USA2	44.49	13.54	30.44	27.25	64.01
Procaine BP	8	Sweden	23.74	3.09	13.02	18.59	28.70
Sodium BP IM	8	Sweden	15.93	12.12	76.08	10.07	45.74

AUC (0–tlast): area under the curve from time 0 to the last data point computed by the linear trapezoidal rule without extrapolation to infinity. For sodium BP after IM administration (Sweden), the observed AUC was largely underestimated due to the sparse sampling during the dosing interval in the case of the multiple dose administration. This is also the case for the USA1 data, as the last sample was taken 12 h after the administration of procaine BP.

Inspection of Table 2 shows that plasma clearance was lower in Swedish and USA2 horses than in French horses (approximately 25 and 47%, respectively). It can be observed that the half-life was relatively similar for horses in each of the three datasets, the differences in clearance being largely compensated for by differences in the volumes of distribution.

### 3.2. Population modeling: IV data

**Figure 5:**
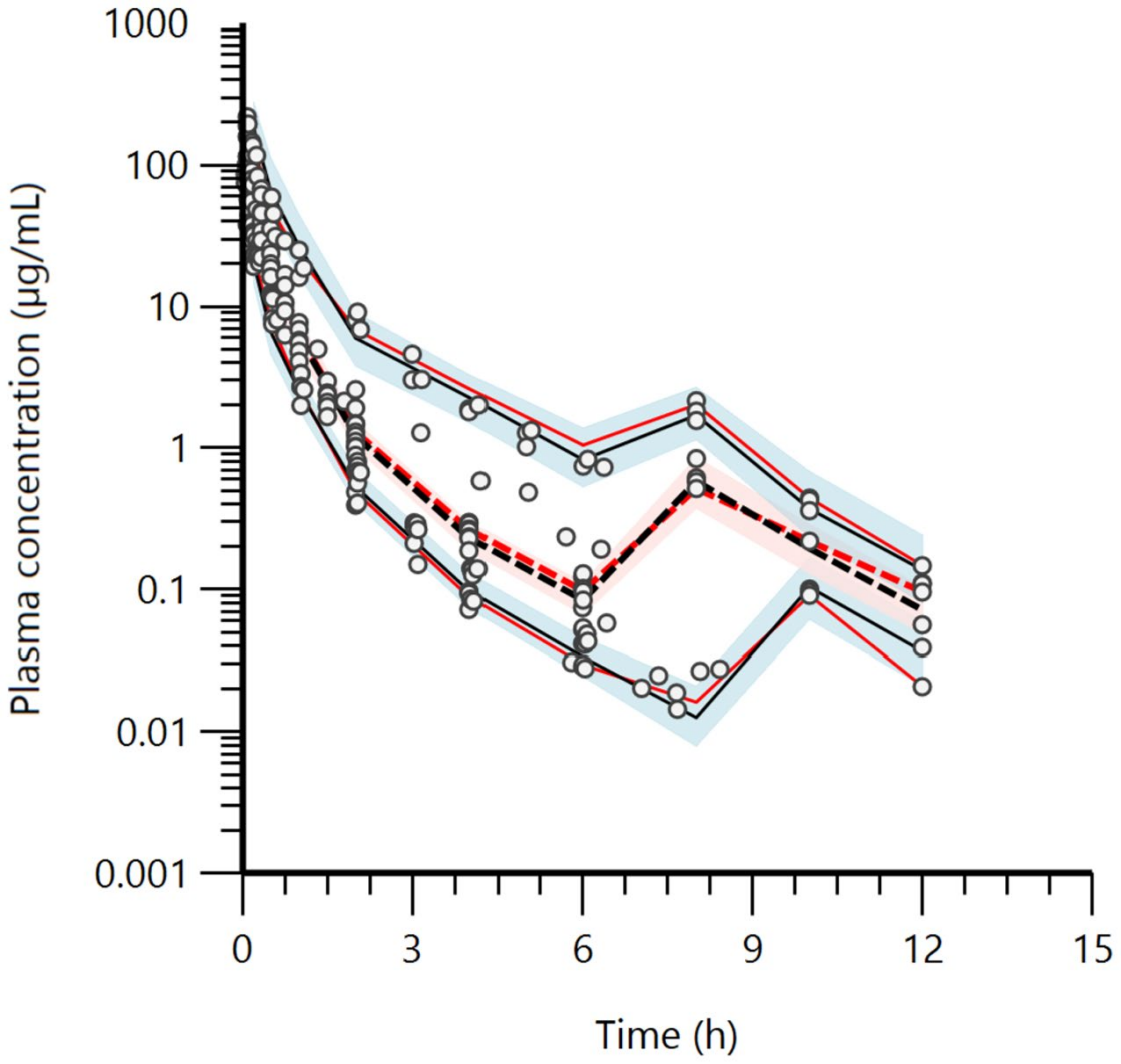
Visual predictive check (VPC) for the IV route obtained with 500 replicates of each horse. The observed quantiles are the 10^th^ (lower), 50th (middle), and 90^th^ (upper), denoted as red lines, with their 90% confidence intervals of predicted quantiles (shaded area). They are rather well superimposed onto the corresponding predictive check quantiles (black lines) over the observed data. The rebound at 8 h is due to the second IV administration in the case of USA1, 6 h after the first one.

Parameters obtained *via* the NCA were used as initial values for the population PK model. In a first step, IV data were modeled to estimate basic parameters of the selected 3-compartment model with “source of dataset” as a covariate for plasma clearance. Visual predictive checks (VPCs) evaluating the capacity of the IV model to reproduce the original dataset are presented in Figure 5. Other goodness-of-fit (GOF) plots for the IV data supporting the 3-compartmental structural model, the exponential model for the random component, and the additive plus multiplicative model for the error submodel, which were used to analyze all the data, are given in Supplementary Figures 4A–E.

Typical values of the primary structural parameters of the model (theta values), their associated CV%, and the SD of the residual for the IV model are given in Table 4.

**Table 4:** Population primary parameters of BP after an IV administration in horses, as obtained with a 3-compartment model with “source of dataset” as a covariate for plasma clearance (estimates, CV%, 2.5, and 97.5% percentiles).

Parameters	Estimate	Units	SE	CV%	2.5% CI	97.5% CI
tvVc	106	mL/kg	25.6	24.14	55.6	157
tvV2	46.3	mL/kg	14.0	30.29	18.6	74.0
tvV3	50.4	mL/kg	14.6	28.94	21.6	79.2
tvCl (France)	481	mL/kg/h	106	22.10	271	691
tvCl (Sweden)	243	mL/kg/h				
tvCl (USA1)	424	mL/kg/h				
tvCl (USA2)	420	mL/kg/h				
tvCl2	126	mL/kg/h	64.4	51.16	−1.1997	253.1218
tvCl3	25.0	mL/kg/h	8.7	34.79	7.8	42.1
tvCMultStdev	0.152817042	scalar	0.00752	4.92	0.13797	0.16766
dCldSource_of_data1 (Sweden)	−0.68325471	scalar	0.09201	13.47	−0.86481	−0.50170
dCldSource_of_data2 (USA1)	−0.12576635	scalar	0.10289	81.81	−0.32879	0.07726
dCldSource_of_data3 (USA2)	−0.13609778	scalar	0.05041	37.04	−0.23557	−0.03663
stdev0	0.000713844	μg/mL	0.00729	1020.91	−0.01367	0.01509

Vc: volume of the central compartment; V2: volume of the superficial peripheral compartment; V3: volume of the deep peripheral compartment; CL: plasma clearance; CL2: distribution clearance between central compartment and compartment 2; CL3: distribution clearance between central compartment and compartment 3; CMultStdev: multiplicative component of the error model, which should be read as a CV of 15.3%; stdev0: additive component of the residual error model; dCldSource_of_data: typical value of the “source of dataset” covariate for Sweden, USA1, and USA2. The covariate “dCldSource_of_data” is significant when its 95% confidence interval excludes 0. The covariate “dCldSource_of_data” was significant for Sweden and USA2, but not for USA1, meaning that plasma clearance in USA1 horses did not differ significantly from plasma clearance in French horses (reference). Plasma clearance in Swedish horses was approximately 50% of that of French horses, and clearance in USA2 was 87.3% of that of French horses.

Population secondary parameters of BP after IV administration are given in Supplementary Table 5. Estimates of the random effects for the IV model are given in Table 5. Supplementary Table 6 presents the full OMEGA matrix for the IV route.

**Table 5:** Estimates of the random effects (full variance/covariance matrix) and shrinkage for the four IV data sets.

Label	nVc	nCl	nV2	nV3	nCl2	nCl3
Omega (variance/covariance)						
nVc	0.4450					
nCl	0.3552	0.2944				
nV2	0.4165	0.3803	0.6459			
nV3	0.4298	0.3614	0.5405	0.5219		
nCl2	0.4600	0.4480	0.8423	0.6532	1.1466	
nCl3	0.3975	0.3508	0.5711	0.5091	0.7254	0.5207
**BSV%**	**75**	**59**	**95**	**83**	**147**	**83**
Correlation						
nV	1					
nCl	0.9813	1.0000				
nV2	0.7768	0.8721	1.0000			
nV3	0.8918	0.9220	0.9308	1.0000		
nCl2	0.6439	0.7711	0.9787	0.8444	1.0000	
nCl3	0.8258	0.8960	0.9847	0.9766	0.9388	1.0000
**Shrinkage (from 0 to 1)**	**0.24**	**0.24**	**0.27**	**0.27**	**0.31**	**0.28**

Between-subject variability was computed using equation (2). Between-subject variability (BSV) and shrinkage are shown in bold. Shrinkage was rather low, indicating that the data were sufficiently rich to enable a proper estimate of the BSV, which was high due to the heterogeneity of the horses in the dataset.

### 3.3. Population modeling: IV and IM data

Finally, all of the data (IV and IM) were modeled simultaneously; however, given the size of the model and the number of parameters to be estimated, the disposition parameters for French horses (CL, CL2, CL3, Vc, V2, and V3) were used as a reference (i.e., no covariate). The “source of dataset” covariate for the clearances of Swedish, USA1, and USA2 horses were set (frozen) at the optimal values obtained with the analysis of IV data only (see Table 3). The OMEGA component and BSV were reassessed for all parameters having a random component.

**Figure 6:**
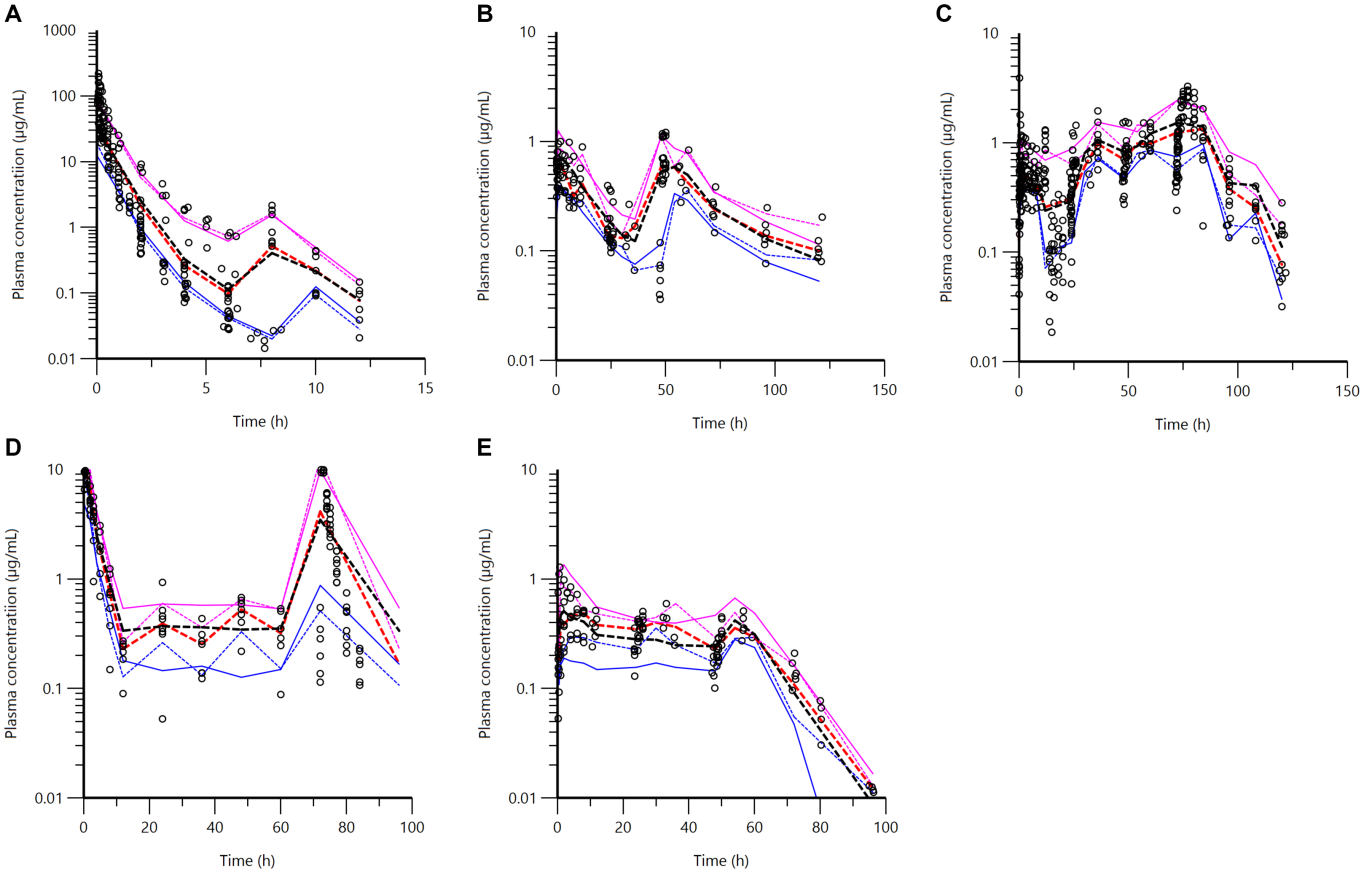
Visual predictive check (VPC) obtained with 500 replicates of each horse, for each stratification (IV, IM for each formulation) for the final model. Blue, red, and magenta dashed lines: observed 20th (lower line), 50th (middle line), and 80th quantiles (upper line); blue, red, and magenta full lines: predicted quantiles; open black points: observed data. **(A)** IV route in French, Swedish, USA1, and USA2 horses. **(B)** Combination of procaine and benzathine BP in French horses. **(C)** Procaine BP in French, Swedish, Japanese, and USA1 horses. The values located below the 20% quantile between the times 12 and 24 h (lower blue lines) are observed data from USA1. **(D)** Sodium BP in Swedish horses. **(E)** Penethamate in French horses.

Figure 6 presents VPC plots for the different routes (IV, IM) and the different IM formulations (procaine, combination procaine and benzathine, penethamate, and sodium BP). Other goodness-of-fit plots are provided in Supplementary Figures 5A–J. These figures support the previous adjustments stratified by formulation and route of administration, and they indicate that it was reasonable to postulate that the disposition of BP as a substance can be described by a set of typical values of common parameters, regardless of its formulation and route of administration in France, Sweden, the USA, and Japan.

A selection of typical values for the primary structural parameters of the model (theta values) and the secondary parameters (mean absorption time, lag time, and bioavailability) are given in Table 6. All the values are provided in Supplementary Table 7. Supplementary Table 8 lists the secondary parameters, and the corresponding full OMEGA matrices are given in Supplementary Tables 9–12.

**Table 6:** A selection of typical values for primary and secondary parameters of BP as obtained by the full population model, integrating the different modalities of BP administration (IV and IM) with different formulations.

Parameter	Estimate	Units
Plasma clearance of Japanese horses (Bayesian estimate)	411	mL/kg/h
Plasma clearance of Swedish horses having received IM procaine and sodium BP (Bayesian estimate)	351	mL/kg/h
MAT of BP after administration of sodium BP (initial rapid phase of absorption) in Swedish horses (Geepenil ^®^)	0.976	h
MAT of BP after administration of sodium BP (second slow phase of absorption after Tlag) in Swedish horses (Geepenil ^®^)	4.03	h
Lag-time between Ka1 and Ka2 for sodium BP in Swedish horses (Geepenil ^®^)	0.471	h
Bioavailability for sodium BP in Swedish horses. (Geepenil ^®^)	89.1	%
MAT of BP after administration of procaine BP for the combination of procaine and benzathine BP (Duplocilline^®^) in French horses	12.1	h
Bioavailability of BP from procaine BP for the combination of procaine and benzathine BP (Duplocilline^®^) in French horses	82.2	%
MAT of BP after administration of benzathine for the combination of procaine and benzathine BP (Duplocilline^®^) in French horses	107	h
Bioavailability of BP from benzathine BP for the combination of procaine and benzathine BP (Duplocilline^®^) in French horses	100.0	%
MAT of BP after administration of procaine BP, French formulation (Depocilline^®^)	21.46	h
MAT of BP after administration of procaine BP, Swedish formulation (Penovet^®^)	20.64	h
MAT of BP after administration of procaine BP, USA1 formulation (Norocillin^®^)	230	h
MAT of BP after administration of procaine BP, Japanese formulation	21.55	h
Bioavailability for BP from procaine BP after administration of procaine BP, French formulation (Depocilline^®^)	100.0	%
Bioavailability of BP from procaine BP, Swedish formulation (Penovet^®^)	99.96	%
Bioavailability of BP from procaine BP, USA1 formulation (Norocillin^®^)	99.97	%
Bioavailability of BP from procaine BP, Japanese formulation (Procaine BP G sol for Animals “KS”^®^)	99.99	%
Bioavailability of BP from penethamate (Penetavet^®^)	68.8	%
CV for the multiplicative component of the residual error	0.283	Scalar

Associated CV% and SE as a measure of precision of theta estimates were not computed due to the size of the model that would have required several weeks of computation by a bootstrap method. The disposition parameters (CL, CL2, CL3, Vc, V2, and V3), as well as the “source of dataset” covariates for the clearances under IV for Swedish, USA1, and USA2 horses, were frozen at the optimal values obtained with the analysis of IV data only (see Table 3) and are not reported again in this table. Mean absorption time (MAT) was computed from the corresponding estimated 1/Ka and taking into account the covariate, if any.

The bioavailability of BP for procaine BP was nearly complete for all formulations tested, including that of USA1. A value of 99% indicates that the ilogit transformation prevented the estimation of bioavailability above 100% in certain cases.

### 3.4. PK/PD integration and Monte Carlo simulations

**Figure 7:**
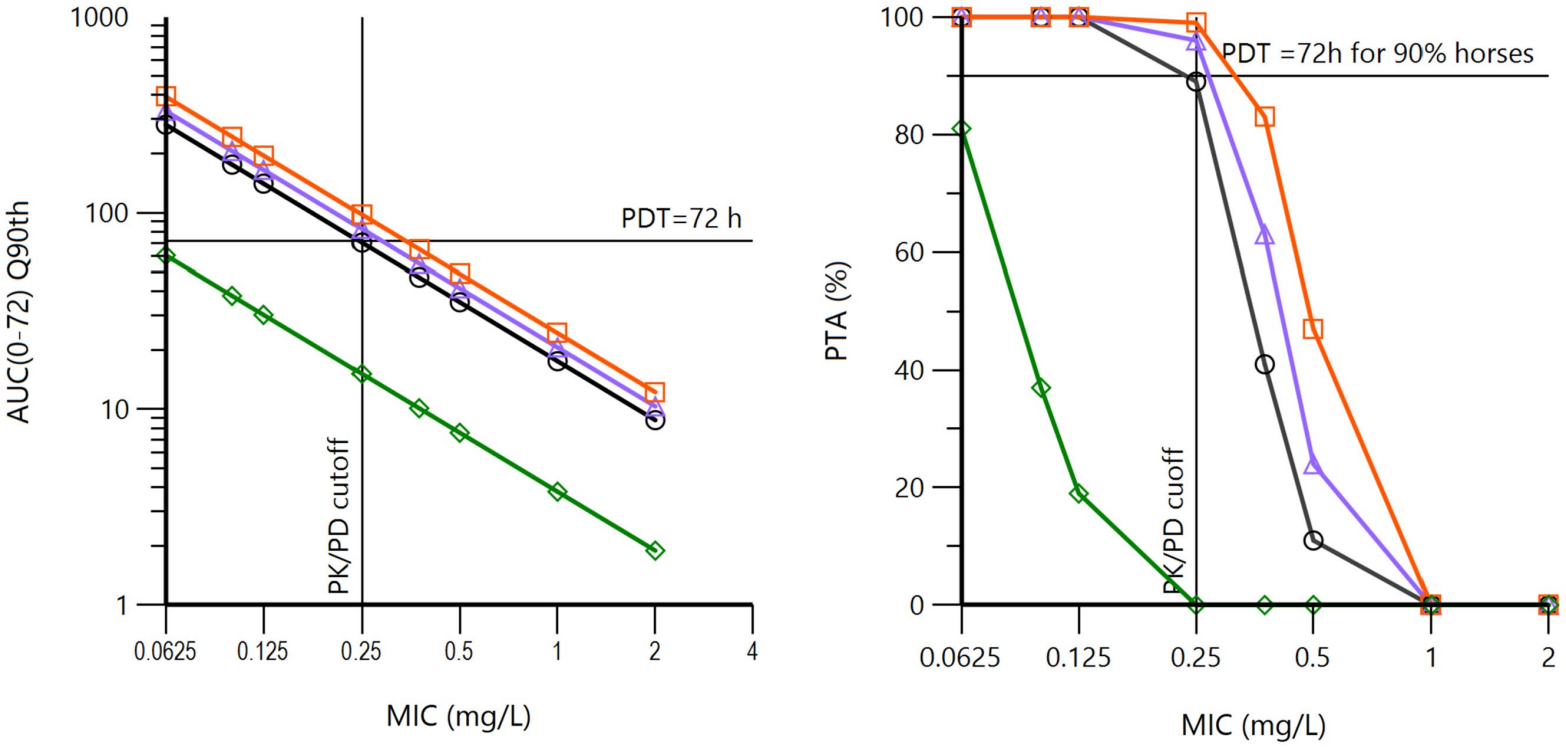
Left: 90th quantiles of *f*AUC/MIC (log10 scale) vs. MIC (log2 scale) for procaine BP at a daily dose of 12.360 mg/kg (22,000 IU/kg) for three days (72 h). The pharmacodynamic target (PDT) was fixed to 72 h. These curves were obtained *via* Monte Carlo simulations (*n* = 5,000) of the population model, with global estimates of the pharmacokinetic parameters of BP from procaine BP from the French (black curve), Japanese (purple curve), Swedish (red curve), and USA1 (green curve) datasets, each nation having been simulated with its own covariate for plasma clearance. It can be noted that the French, Japanese, and Swedish data lead to the same results (PK/PD cutoff value of 0.25 mg/L), whereas the USA1 data are very different, with a PK/PD cutoff value close to 0.0625 mg/L. Right: the corresponding probability of target attainment (PTA). It can be noted that the French, Japanese, and Swedish data lead to the same results (PK/PD cutoff value of 0.25 mg/L), whereas the USA1 data are very different, with a PK/PD cutoff value of 0.0625 mg/L for a PTA of 80%.

The ultimate goal of this data analysis was to compute the different PK/PD cutoff values corresponding to the different routes of BP administrations (IV, IM), for different IM formulations and dosage regimens. The full model was used for Monte Carlo simulations. For procaine BP, PK/PD cutoff values were computed for France, Sweden, USA1, and Japan, accounting for their clearance as a covariate. All these results are given in Supplementary Tables 13–21. For procaine BP, when considering the PK/PD index *f*AUC/MIC and a PDT of 72 h for a duration of treatment of 72 h (daily dose of 12.36 mg/kg or 22,000 IU/kg with a dosing interval of 24 h), the PK/PD cutoff value was 0.25 mg/L for France, Sweden, and Japan but only approaching 0.0625 mg/L in the case of USA1 (Figure 7). This means that in 90% of horses, for formulations investigated in France, Sweden, and Japan, the average free plasma concentration over the 72 h of treatment would be at least equal to 0.25 mg/L. For the USA1 formulation, the Q90% for an MIC of 0.0625 mg/L was 61 h, i.e., approaching the PK/PD target of 72 h. This is likely sufficient for *Streptococcus* spp. having an MIC90 ≤ 0.06 mg/L (Wayne, 2018).

Doubling the dose (a daily dose of 24.72 mg/kg) doubled the PK/PD cutoff value to 0.5 mg/L, and a similar result was obtained with a regimen of 12.36 mg/kg at 12 h intervals over three days; this last regimen is the dosage regimen most often recommended in various textbooks.

BP plasma concentrations resulting from Duplocilline^©^ (a combination of procaine BP and benzathine BP) were simulated at a dose of 12.36 mg/kg administered twice with a 48-h interval. The duration of treatment is 96 h rather than 72 h because the interval of administration in clinical settings is 48 h, suggesting a total treatment duration of 96 h with two doses. Thus, the PDT of *f*AUC/MIC was set to 96 h. With this regimen, the PK/PD cutoff value of 0.25 mg/L was not achievable. For an MIC of 0.0625 mg/L, the Q90% is at 101 h, i.e., just above the PDT of 96 h, and this is likely sufficient for *Streptococcus* sp. having an MIC90 ≤ 0.06 mg/L.

For penethamate and the SPC regimen, the PK/PD cutoff value was 0.0625 mg/L.

**Figure 8:**
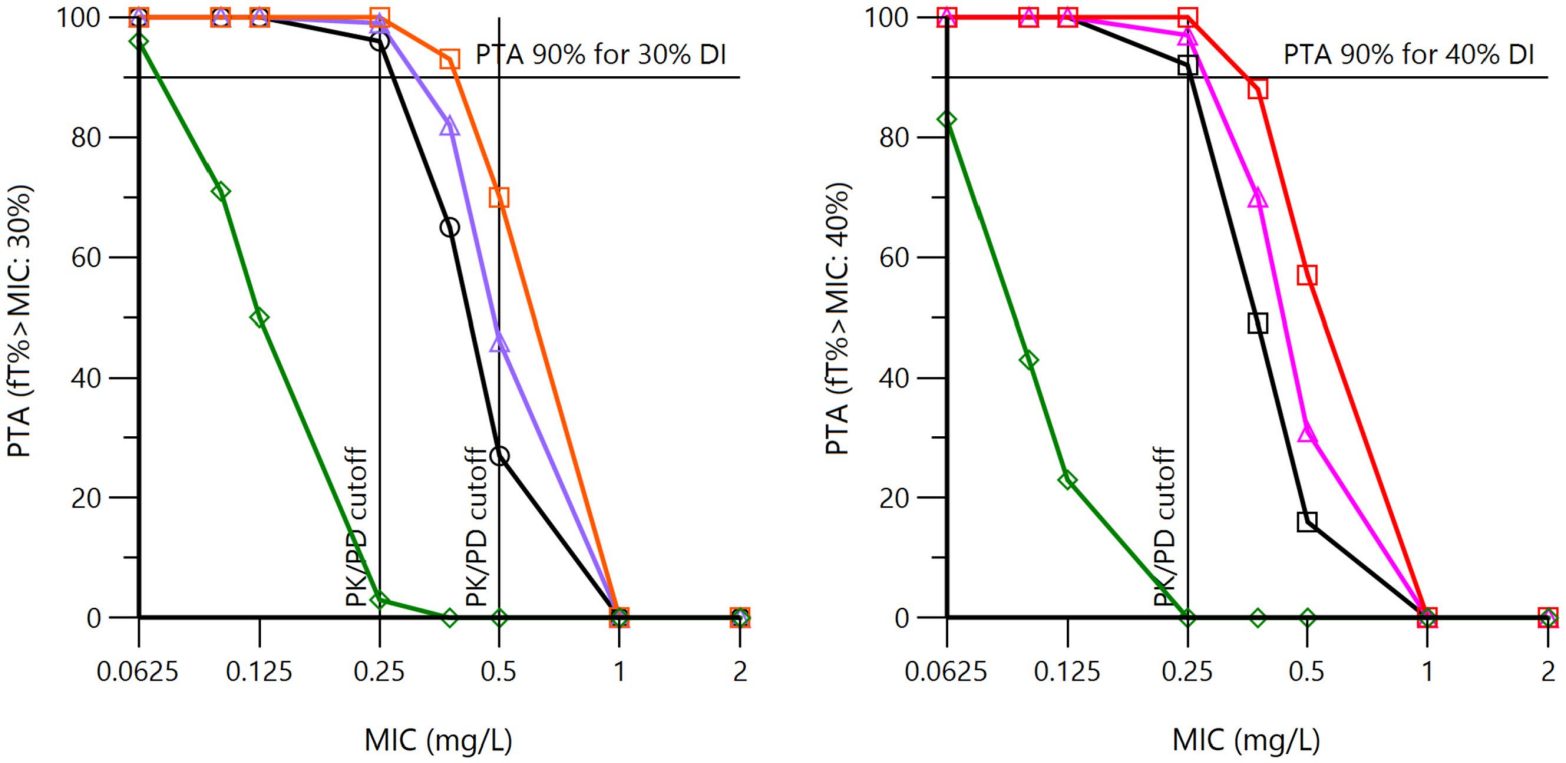
Probability of target attainment (PTA) for 30% (left) and 40% (right) of the dosing interval for procaine BP at a daily dose of 12.36 mg/kg (22,000 IU/kg) for three days (72 h). The pharmacodynamic target (PDT) is set at 30% or 40% of the dosing interval (DI), which means that the MIC selected as the PK/PD cutoff value must guarantee that the free plasma BP concentration is at least equal to or higher than this MIC over 30 or 40% of the DI (i.e., 21.6 and 28.8 h *in toto* over 72 h) in 90% of horses. These curves were obtained by Monte Carlo simulations (*n* = 5,000) of the population model, with global estimates of the pharmacokinetic parameters of BP from French (black curve), Japanese (purple curve), Swedish (red curve), and USA1 (green curve) datasets, each nation being simulated with its own covariate for plasma clearance.

When *f*T > MIC was selected as the PK/PD index with a PDT of 30 or 40% of the dosing interval, the PK/PD cutoff value was also 0.25 mg/L for the French, Swedish, and Japanese procaine formulation, whereas no procaine BP formulation reached the PDT of 30% for an MIC of 0.50 mg/L over 72 h with daily administration of 12.36 mg/kg (22,000 IU/kg) (Figure 8). For penethamate and for the combination of procaine BP and benzathine BP, the PK/PD cutoff value was 0.0625 mg/L, confirming the conclusion reached with *f*AUC/MIC as the index.

Inspection of Figure 8 shows that using *f*T > MIC as the PK/PD index results in the selection of the same PK/PD cutoff of 0.25 mg/L for the French, Japanese, and Swedish formulations, as with *f*AUC/MIC as the PK/PD index. The USA1 data were very different, with a PK/PD cutoff of 0.0625 mg/L for a PDT of 30%.

For IM sodium BP (Supplementary Table 19), the objective of 40% of the dosing interval was found to be achieved for an MIC of 0.25 mg/L for a dose of 12.36 mg/kg at 6, 8, or 12 h intervals. For an MIC of 0.5 mg/L, the objective of 30% of the dosing interval was also achieved for all regimens. For an MIC of 1 mg/L, only an IM dosage regimen of 12.36 mg/kg every 6 or 8 h was found to reach a PDT of 40% of the dosing interval in 90% of horses.

For an MIC of 0.25 mg/L, 24-h infusions of a total dose of 12.36 mg/kg (22,000 IU/kg) were found to to maintain BP plasma concentrations above this MIC for more than 22 h out of the 24-h infusion for 90% of the horses across the three datasets examined (France, Sweden, and USA2). On the other hand, for an MIC of 0.50 mg/L, only the Swedish horses were able to maintain values above this MIC for 21 h. To exceed the MIC of 0.5 mg/L, a total dose of 18,840 μg/kg (USA2) or 24,720 μg/kg (France) was required. These differences reflect the differences in plasma clearances.

With a series of four IV bolus doses administered at 6-h intervals, it was impossible to achieve a PK/PD cutoff of 0.25 mg/L with a total dose of 12.360 mg/kg. Doubling the dose allowed only Swedish horses to achieve this PK/PD cutoff of 0.25 mg/L. With a total dose of 37.08 mg/kg or 66,000 IU per day, a PK/PD cutoff of 0.25 mg/L could be achieved for Swedish and USA2 horses, but not for French horses. With a total dose of 49.456 mg/kg (i.e., 12.36 mg/kg four times at 6-h intervals), the PK/PD of 0.25 mg/L could be achieved for all horses, validating the most common dosage regimen for IV BP.

## 4. Discussion

The purpose of this project was to establish a PK/PD cutoff value for BP in horses that would be generic enough to be suitable for different organizations responsible for establishing CBs in veterinary medicine. Accordingly, this PK/PD cutoff value should ideally take into account the multiple modes of administration of BP in horses, including the route of administration (IV bolus, IV infusion, and IM), the formulations used (LA and short-acting), and the different dosage regimens (doses, administration intervals) that are recommended by experts in the field or in the SPC of marketed formulations. It was not *a priori* obvious that a single value could reasonably cover a majority of situations, but our analysis allows us to conclude that a PK/PD cutoff of 0.25 mg/L is consistent with most current veterinary practices.

A PK/PD cutoff is a variable that is essentially of a PK nature and that must meet the statistical requirement to apply to 90% of the targeted horse population. Given our objectives, it was necessary to study the PK of different formulations of BP representative of practices in different regions of the world. This was achieved with data collected in Europe (France and Sweden), in North America (the USA) and in Asia (Japan), particularly in the case of procaine BP, four different formulations of which have been aggregated in our database. Two other LA formulations were also assessed, namely, a combination of procaine BP and benzathine BP and a formulation of penethamate, a prodrug of BP. In addition, data corresponding to repeated IM administration of sodium BP, as an alternative to procaine BP, were also included in the base. The IV route is also commonly used, in the form of a series of IV bolus doses or, if possible, a continuous-rate infusion (CRI) (Magdesian, 2015). We also collected data obtained by the IV route and computed corresponding PK/PD cutoffs for different regimens. Importantly, the IV data were the only ones that allowed for direct comparison of the different sources of data, ensuring comparability of the different tested formulations without the risk of a confounding effect associated with the source of the data.

The VetCAST approach requires reanalyzing raw data, rather than considering published parameters, in performing Monte Carlo simulations. As such, it is inevitable that the aggregation of datasets obtained with different experimental designs, having enrolled different numbers of horses, and having used more or less sensitive analytical techniques in terms of levels of quantification (LOQ) leads to great heterogeneity in the informative value of each dataset. Technically, our datasets are regarded as unbalanced. This is a situation frequently encountered in human medicine during meta-analyses of pharmacokinetic data collected during the different phases of development of a drug. A non-linear mixed-effects model is recommended for the analysis of this type of data (Schoemaker and Cohen, 1996). This is supported by the FDA guidance on population modeling, which indicates that population PK modeling is the only appropriate tool for meta-analysis of data retrieved from different studies with unbalanced designs (Food and Drug Administration, 1999). A framework for meta-analysis of veterinary drug pharmacokinetic data using mixed-effects modeling has previously been presented (Li et al., 2015) and has already been used to compute PK/PD cutoff values for florfenicol in cattle (Toutain et al., 2019) and amoxicillin in pigs (Rey et al., 2014).

There are three major aspects to the development of a population pharmacokinetic model: (i) selection of a structural model; (ii) inclusion of a statistical model describing variability in the structural model; and (iii) inclusion of covariate models to explain variability. Structural models describe the typical plasma concentration *vs* time course within the population. For the present meta-analysis, a 3-compartment model was selected, while modeling with a 2-compartment model is generally reported in the literature (Firth et al., 1986; Horspool and McKellar, 1995). This is explained by the fact that quantifiable data were obtained up to 8 h after IV administration in the case of the French dataset, thanks to a low LOQ of 10 ng/mL. This rendered a third phase of the disposition of BP identifiable. This was not the case for the Swedish and USA2 datasets, for which the last quantifiable concentration occurred at 6 h post-administration, leading the authors to analyze these data under a 2-compartment model in their original publications (Olsén et al., 2018; Wilson et al., 2022). When all these data were analyzed simultaneously using the NLME model, the 3-compartment model prevailed. Animals for which data were not collected or quantified beyond 6 h post-administration were treated as censored between 6 and 8 h post-administration. These considerations regarding the contributions of the NLME model are not only of academic interest. The identification of a final phase of BP disposition under the IV route enables correct interpretation of the absorption parameters obtained by the extravascular route; and, in particular, this allows for the identification of ‘flip-flops’ in the absorption *vs* elimination phases. When the terminal half-life after IM administration exceeds that of the IV route (here, 1.5 h), the occurrence of a flip-flop can be deduced. This was the case for all investigated formulations, including sodium BP, explaining the fact that a series of intramuscular administrations of sodium BP was able to maintain a sustained BP plasma concentration despite the short half-life of BP and was proposed as an alternative to procaine BP (Olsén et al., 2013).

The second component of an NLME model consists of the statistical models that account for random variability in plasma concentration within the population. For the final model (without covariates), the BSV for clearance was 26.4%. This is rather moderate for a population of horses investigated at five different geographical sites spread across three different continents. This was not the case for the different volumes of distribution, with BSVs of 174% for Vc, 56.7% for V2, and 42% for V3. This difference in BSV between clearance and volumes of distribution should be considered when calculating a PK/PD cutoff value using either *f*AUC/MIC or *f*T > MIC. The two determinants of AUC are clearance and bioavailability. For the procaine BP formulations, bioavailability was 100% for the French, Swedish, and Japanese formulations, which means that the only source of variability of the AUC for these formulations was plasma clearance. On the other hand, the *f*T > MIC index depends on the shape of the plasma concentration profiles, and therefore not only on plasma clearance but also on the volumes of distribution. We should therefore expect, all other things being equal, greater variability in the distribution of this index, with a risk of calculating lower PK/PD cutoff values with *f*T > MIC than with *f*AUC/MIC. In fact, this was not the case for the present meta-analysis, the two indices each having led to an estimated PK/PD cutoff value of 0.25 mg/L; however, these statistical considerations regarding BSV are additional arguments for favoring *f*AUC/MIC for LA formulations (Toutain et al., 2021), including beta-lactam antibiotics.

The final component of a NLME model is the inclusion (or not) of covariates. Covariates are intended to account for variability, and the most frequently included covariates are subject characteristics (age, breed, sex, etc.). Here, no biological covariates were considered in order to compute the most generic PK/PD cutoff values possible. However, we tested the influence of source of dataset as a factor in variability and in order to assess the consistency of the datasets. Only the IV route allowed for such a comparison. Statistically significant differences in clearances appeared between France (the reference source, with a clearance of 481 mL/kg/h) and Sweden (50% of the reference value) and between France and USA2 (87% of the reference value), but not between France and USA1. In the case of Sweden, inspection of Supplementary Figure S4 indicates that 3 of the 4 horses in the trial had significantly higher plasma concentration profiles than the other horses in the study. The origin of this difference remains unexplained, especially since the Bayesian estimate for the 8 other Swedish horses that did not receive BP by the IV route does not suggest that Swedish horses are different from other horses. Beyond this difference, the plasma clearances reported in the present publication remain within the same order of magnitude as those already reported in the literature, i.e., 514 mL/kg/h (Horspool and McKellar, 1995) and 510 mL/kg/h (Firth et al., 1986). For plasma clearance, we retained this “source of dataset” covariate in the full model to better estimate the bioavailability of the formulations that were administered to the same horses as those that received BP by the IV route (French and USA1 data). For Swedish horses that received procaine BP and sodium BP and for Japanese horses that received procaine BP, we also introduced the “source of dataset” covariate for clearance, which was therefore estimated indirectly *via* a Bayesian method. The rationale for this approach is our assumption, based on the results of the NCA, that the Swedish and Japanese procaine BP formulations also had very high bioavailability, like the French and USA1 formulations. We estimated the bioavailability of these different formulations of procaine BP to be approximately 100%. This high bioavailability of 100% for procaine BP administered IM has previously been reported by others (Firth et al., 1986). However, the total bioavailability of procaine BP was surprising in the case of the USA1 formulation, for which plasma concentrations were much lower than for the other investigated formulations (Swedish, French, and Japanese). The explanation is that the USA1 data were collected only during the first 12 h after administration and showed a very flat initial profile. Consequently, a very slow absorption rate constant was estimated for USA1, with a mean absorption time (MAT) of 230 h, in comparison to approximately 21 h for the other three formulations. In other words, the NLME model predicts that, for USA1, BP plasma concentrations will be maintained at this low level for several days; however, in the absence of data beyond 12 h, it is advisable to remain cautious about this conclusion.

A very long MAT was also estimated for the benzathine BP Duplocilline (107 h) delivered IM, which is consistent with what has been reported for benzathine BP in cattle (Papich et al., 1994). Our data support their conclusion, which is that BP plasma concentrations following benzathine BP administration remained below the level of susceptibility for most pathogens.

For sodium BP, IM bioavailability was estimated at 89%, and a MAT of 4 h was observed after a short delay following a rapid initial phase of absorption. This indicates a flip-flop process for a fraction of sodium BP, allowing therapeutic concentrations to be maintained over the 12-h dosing interval. For penethamate, IM bioavailability was incomplete (68.8%), with a relatively long MAT (28–65 h). It can be noted that this formulation gave rise to relatively significant local reactions, and aside from the fact that it does not contain procaine, which is an advantage for doping control in racehorses, this formulation has lower performance in terms of BP exposure than procaine BP formulations.

The main result of this meta-analysis is that a PK/PD cutoff value of 0.25 mg/L can be guaranteed in 90% of horses for the usual dosage of 12.4 mg/kg per day with the three formulations of procaine BP that have been documented over a sufficient period of time to qualify the full plasma concentration profile (the France, Sweden, and Japan datasets). In contrast, the PK/PD cutoff value is only around 0.0625 mg/L in the case of penethamate and the combination of procaine and benzathine BP, which may still be acceptable for treatment of more sensitive pathogens such as *Streptococcus* spp. In the case of a 24-h continuous IV infusion, and under analysis using *f*T > MIC as a PK/PD index, a PK/PD cutoff of 0.25 mg/L is also achievable with a total dose of 12.4 mg/kg, but not by splitting this dose into a series of three IV bolus doses at 8-h or 6-h intervals, indicating the PK/PD advantage of a continuous infusion over intermittent administration. For IV bolus administration, the routine regimen of 12.36 mg/kg four times at 6-h intervals was also able to achieve a PK/PD cutoff of 0.25 mg/L for all horses.

Finally, a PK/PD cutoff of 0.25 mg/L may be considered as a generic cutoff in equine medicine. In addition, this estimate is likely to be conservative, as the free fraction of drug was computed by simply scaling total BP plasma concentrations by the unbound fraction of 0.4. This scaling ineluctably adds a source of variability that does not exist (i.e., *f_u_* variability) to the computed free concentration, thus inflating the estimate of the 90% quantile of the PK/PD index; see Toutain et al. (2023) for explanation. For human medicine, EUCAST has proposed a “nonspecific PK/PD breakpoint” for BP of 0.25 mg/L (S) and 2 mg/L (R) (Anonymous, 2010). USCAST has proposed a CB of 0.25 mg/L for *Streptococcus* spp. and 0.12 mg/L for *S. aureus* (Anonymous, 2021). For veterinary medicine, the VAST/CLSI has established CBs for both *Streptococcus* spp. and *Staphylococcus* spp. of <=0.5, 1, and ≥ 2 mg/L for Susceptible (S), Intermediate (I), and resistant (R), respectively (Wayne, 2018). All these results are consistent with the fact that the PK parameters of BP (plasma clearance, volume of distribution, unbound fraction) are practically identical in humans (Anonymous, 2010) and horses and that both procaine BP and sodium BP have excellent bioavailability in horses when administered by the IM route.

## Data availability statement

The original contributions presented in the study are included in the article/Supplementary material, further inquiries can be directed to the corresponding author.

## Ethics statement

The animal study was approved by Ecole nationale Veterinaire de Toulouse. The study was conducted in accordance with the local legislation and institutional requirements.

## Author contributions

EL: Conceptualization, Data curation, Funding acquisition, Investigation, Supervision, Validation, Writing – review & editing. AB-M: Conceptualization, Funding acquisition, Writing – review & editing, Project administration, Resources. LC: Writing – review & editing, Data curation, Investigation. JD: Investigation, Writing – review & editing. AF: Investigation, Writing – review & editing, Conceptualization. BK: Investigation, Writing – review & editing, Funding acquisition. TK: Funding acquisition, Investigation, Writing – review & editing. ML: Investigation, Writing – review & editing. YM: Investigation, Writing – review & editing, Methodology. LO: Investigation, Writing – review & editing, Funding acquisition. LP: Writing – review & editing, Formal analysis, Methodology. FP: Writing – review & editing, Investigation. BR: Investigation, Writing – review & editing, Data curation. ES: Investigation, Data curation, Writing – review & editing, Methodology. KW: Investigation, Writing – review & editing, Funding acquisition. P-LT: Formal analysis, Writing – original draft.
