# Supplementary material for: Pharmacokinetic–pharmacodynamic cutoff values for benzylpenicillin in horses to support the establishment of clinical breakpoints for benzylpenicillin antimicrobial susceptibility testing in horses
Source: Front Microbiol. 2023 Oct 25;14:1282949. doi: 10.3389/fmicb.2023.1282949 (PMC10634207; doi:10.3389/fmicb.2023.1282949)
Supplement: Supplementary file 2 [file Data_Sheet_1.docx]

Supplementary Material

# Pharmacokinetic-pharmacodynamic cutoff values of benzylpenicillin in horses to support the establishment of clinical breakpoints for benzylpenicillin Antimicrobial Susceptibility Testing in horses

**Elodie Anne Lallemand, Alain Bouquet-Mélou, Laura Chapuis, Jennifer Davis, Aude A. Ferran, Butch Kukanich, Taisuke Kuroda, Marlène Z. Lacroix, Yohei Minamijima, Lena Olsén, Ludovic Pelligand, Felipe Ramon Portugal, Béatrice B. Roques, Elizabeth M. Santschi, Katherine E. Wilson, Pierre-Louis Toutain***

**Correspondence:** Pierre-Louis Toutain: [pltoutain@wanadoo.fr](mailto:pltoutain@wanadoo.fr)

# Supplementary data1. Details of the French and Japanese analytical methods for benzyl penicillin

**Penicillin G assays (France)**

Penicillin G (PeniG) assays were performed on an Ultra High Performance Liquid Chromatography system (Acquity UPLC, Waters) coupled to a Xevo triple quadrupole mass spectrometer (Waters, MA, USA). PeniG plasma samples (100 µL) were extracted from the matrix by adding 10 µL of IS (ampicillin 10 µg/mL) and 150 µL of MeOH with 1% formic acid to precipitate the plasma proteins. The mixture was shaken at 10°C for 2 minutes at 1400 rpm (MB-102, Bioer, Hangzhou, China) and centrifuged for 10 min at 4°C and 20000g. Ten microliters of extracted sample were concentrated on an XBridge® C8 Direct Connect HP cartridge (2.1 mm × 30 mm, 10 µm ; Waters) at 1 mL/min flow with 100 % H2O, 0.1% FA for 0.2 min and then eluted on an Acquity HSS T3 column (2.1 mm × 100 mm ; 1.8 µm ; Waters) at 0.3 mL/min with an acidified (FA 0.2%, HFBA 0.02%) H2O/AcN gradient (t0 = 80 % H2O, 0.2 % FA, HFBA 0.02% t3min= 10% H2O, 0.2 % FA, HFBA 0.02%). On-line SPE cartridge and column temperatures were both set at 40°C and analytical run was 4.50 min. PeniG and IS were ionized by electrospray in positive mode (ESI+) with the multiple reaction monitoring (MRM) transitions m/z 365>176 and 350>192, respectively. The retention times were 2.67 min and 1.97 min for PeniG and the IS, respectively. The method was validated with a calibration curve ranging from 0.01 to 10 µg/mL with a quadratic model weighted by 1/X² (X= PeniG concentration). The coefficient of variation (CV %) of the intra-day and inter-day precisions were lower than 9% for both with an accuracy varying from 91 to 108 %. The limit of quantification (LOQ) was 0.01 µg/mL with a precision of 16% and an accuracy of 114%. Extracted samples were stable in the autosampler set at 15°C over 8H.

**Penicillin G assays (Japan)**

Penicillin G (PeniG) assays were performed on an Ultra High-Performance Liquid Chromatography system (Nexera X2 system, Shimadzu) coupled to a QTRAP 4500 mass spectrometer (SCIEX, MA, USA). PeniG plasma samples (100 µL) were extracted from the matrix by adding 20 µL of IS (oxacillin 1 µg/mL) and 600 µL of AcN to precipitate the plasma proteins. The mixture was shaken at room temperature for 5 minutes at 1,000 rpm and centrifuged for 5 min at 4°C and 9100g. Fifty microliters of extracted sample was diluted with 200 µL of water. Five microliters of diluted sample were applied to an ACQUITY UPLC BEH column (100 mm × 2.1 mm; 1.7 µm; Waters) and eluted at 0.4 mL/min flow with an acidified (FA 0.1%) H2O/AcN gradient (t0 = 98 % H2O, 0.1 % FA, t5.5min= 20% H2O, 0.1 % FA). Column temperature was at 40°C and analytical run time was 5.5 min. PeniG and IS were ionized by electrospray in negative mode (ESI-) with the multiple reaction monitoring (MRM) transitions m/z 333>192 and 400>259, respectively. The retention times were 4.04 min and 4.40 min for PeniG and the IS, respectively. The method was validated with a calibration curve ranging from 0.03 to 10 µg/mL weighted by 1/Y² (Y= PeniG concentration). The lower limit of quantitation (LOQ) was 0.03 µg/mL. Intra- and inter-day accuracy and precision were assessed using QC samples at three concentrations of PeniG: 0.03, 0.3 and 8 µg/mL. The coefficient of variation (CV %) of the intra-day and inter-day precisions were ≤5.7%. Intra-assay and inter-assay accuracy were between 91.3 and 112%. Extracted samples were stable in the autosampler set at 4°C over 8 hours.

# Supplementary data2. Phoenix code for the final model

Copy and paste this code into Phoenix; Green sentences (in Phoenix) are comments. All comments start with a # If you have trouble with this code, I can send you a Phoenix file

test(){

####### Phoenix CODE developed by PL Toutain 11/2021 ##############

#Green sentences are comments., all comments start with an #

#Covariate “nation” with France=0 i.e. no covariate, Sweden=1 (IV), Sweden=11 (for IM BP-PROC and IM BP-Na), USA1=2 , Japan=3 and USA2=4) for plasma clearance with a vector nation in data sheet

fcovariate(Nationcode())

#1-Block IV.

##IV data was available for 4 countries (France, USA1 Sweden (code=1) and USA2 and not available for Japan and for Swedish horses having received procaine BP and sodium BP. For the 6 Japanese horses having received procaine BP and the 8 Swedish horses having received procaine BP and sodium BP, the clearances were estimated in a Bayesian way by observing that the exposures to procaine BP were very similar for Japan, Sweden and France. This made it possible to evaluate the bioavailabilities (F) of procaine BP for Japan and Sweden and for the sodium BP of Swedish horses For Swedish horses, not studied by the IV route (SW11), there was no reason to assume that it was the same plasma clearance as those of 4 other Swedish horses that had received BP IV.

deriv(A1IV = - Cl * CIV - Cl2 * (CIV - C2) - Cl3 * (CIV - C3))

deriv(A2IV = Cl2 * (CIV - C2))

deriv(A3IV = Cl3 * (CIV - C3))

dosepoint(A1IV)

CIV = A1IV / V

C2 = A2IV / V2

C3 = A3IV / V3

error(CEpsIV = 0.00792812036590013)

#add+multiplicative error model

observe(CObsIV = CIV + CEpsIV * sqrt(1 + CIV^2 * (CMultStdev/sigma())^2))

# to compute individual AUCs after IV administration

deriv(AUC_IV=CIV)

#1-Block IV of shared parameters common to all data set; no covariate except for plasma clearance

stparm(V = tvV * exp(nV))

stparm(V2 = tvV2 * exp(nV2))

stparm(V3 = tvV3 * exp(nV3))

#Covariate nation for plasma clearance: 0=Fr, 1=SW,11=SW for IM, 2=USA1,3=Japan 4=USA2 (actually no IV experimental data for Japan and SW11 but information on clearance exists throughout extravascular administration and observing that AUC for procaine BP were similar for France (for which IV were available), Japan and Sweden))

stparm(Cl = tvCl * exp(dCldNationcode1*(Nationcode==1)) * exp(dCldNationcode2*(Nationcode==2))* exp(dCldNationcode3*(Nationcode==3))* exp(dCldNationcode11*(Nationcode==11))* exp(dCldNationcode4*(Nationcode==4))* exp(dCldNationcode4*(Nationcode==4)) * exp(nCl))

stparm(Cl2 = tvCl2 * exp(nCl2))

stparm(Cl3 = tvCl3 * exp(nCl3))

stparm(CMultStdev = tvCMultStdev)

# initial values; for the full model,IV parameters were set to their best estimates from the previous data analysis

fixef(tvV(freeze) = c(, 106.184336892764, ))

fixef(tvV2(freeze) = c(, 46.2952044536849, ))

fixef(tvV3(freeze) = c(, 50.4297016344536, ))

fixef(tvCl(freeze) = c(, 480.899353392651, ))

fixef(tvCl2(freeze) = c(, 125.961026710481, ))

fixef(tvCl3(freeze) = c(, 24.9818543761259, ))

#Covariate Nation for plasma clearance: 0=Fr, 1=SW_IV,2=USA1; 3=Japan 4=USA2 11=SW procaine & BP sodium

fixef(dCldNationcode1(enable=c(1))(freeze) = c(, -0.683254, ))

fixef(dCldNationcode2(enable=c(1))(freeze) = c(, -0.125766, ))

fixef(dCldNationcode3(enable=c(1))(freeze) = c(, -0.156691638818043, ))

#To estimate plasma clearance of Swedish horses treated with procaine BP and sodium BP and having no IV data

fixef(dCldNationcode11(enable=c(1)) = c(, -0.314630693388979, ))

#To estimate plasma clearance of Japanese horses treated with procaine BP

fixef(dCldNationcode4(enable=c(1)) = c(, -0.136097, ))

#Full OMEGA for BP disposition as substance (frozen)

ranef(block(nV, nCl, nV2, nV3, nCl2, nCl3)(freeze) = c(1.400552,

0.20942975, 0.067363321,

0.40218913,0.087954509, 0.27982625,

0.34658421,0.06450593,0.14584899, 0.16522015,

0.31712857,0.049439529,0.1098892,0.054298124, 0.23712655,

0.33452693,0.065632766,0.077679041,0.10881838,0.12799386, 0.2143448))

#CV% is a fixed effect

fixef(tvCMultStdev = c(, 0.283453226691478, ))

#tv clearance Swedish horse treated with IV BP

secondary(ClearanceSWIV=tvCl*exp(dCldNationcode1))

#tv clearance for Swedish horse having received procaine BP and sodium BP

secondary(ClearanceSW_peniNa_PROCIV=tvCl*exp(dCldNationcode11))

#tv clearance for Japanese horses having not received an IV BP administration

secondary(ClearanceJP=tvCl*exp(dCldNationcode3))

#secondary tv clearance for USA1

secondary(ClearanceUS1=tvCl*exp(dCldNationcode2))

#tv clearance for USA2

secondary(ClearanceUS2=tvCl*exp(dCldNationcode4))

# Secondary parameters for a 3-cp model (be careful that actually Cl2 must be >Cl3 #to compute these secondary parameters)

secondary(tvKe=tvCl/tvV)

secondary(tvK12=tvCl2/tvV)

secondary(tvK13=tvCl3/tvV)

secondary(tvK21=tvCl2/tvV2)

secondary(tvK31=tvCl3/tvV3)

secondary(tvVss=tvV+tvV2+tvV3)

secondary(tvMRTIV=tvVss/tvCl)

#Block to compute macroparameters for a 3-cpt model

secondary(a0=tvKe*tvK21*tvK31)

secondary(a1=tvKe*tvK31+tvK21*tvK31+tvK21*tvK13+tvKe*tvK21+tvK31*tvK12)

secondary(a2=tvKe+tvK12+tvK13+tvK21+tvK31)

secondary(p=a1-a2^2/3)

secondary(q=2*a2^3/27-a1*a2/3+a0)

secondary(r1=(-(p^3/27))^0.5)

secondary(r2=2*r1^0.3333)

secondary(PHI=acos(-q/(2*r1))/3)

secondary(root1=-(cos(PHI)*r2-(a2/3)))

secondary(root2=-(cos(PHI+2*3.14159/3)*r2-a2/3))

secondary(root3=-(cos(PHI+4*3.14159/3)*r2-a2/3))

root1

root2

root3

#to compute Alpha>Beta>Gamma otherwise A, B and C are false

secondary(tvAlpha=root1>root2 &&root1>root3 && root2>root3?root1:root2)

secondary(tvBeta=root1>root2 &&root1>root3 && root2>root3?root2:root3)

secondary(tvGamma=root1>root2 &&root1>root3 && root2>root3?root3:root1)

secondary(tvHL_alpha=ln(2)/tvAlpha)

secondary(tvHL_Beta=ln(2)/tvBeta)

secondary(tvHL_Gamma=ln(2)/tvGamma)

# alpha>beta>gamma to compute consistently A, B and C

#for a dose of BP of 10000 µg/kg : to edit if you wish another dose

Dose=10000

secondary(tvA=((Dose)/tvV)*((tvK21-tvAlpha)/(tvAlpha-tvBeta))*(tvK31-tvAlpha)/(tvAlpha-tvGamma))

secondary(tvB=((Dose)/tvV)*((tvK21-tvBeta)/(tvBeta-tvAlpha))*(tvK31-tvBeta)/(tvBeta-tvGamma))

secondary(tvC=((Dose)/tvV)*((tvK21-tvGamma)/(tvGamma-tvBeta))*(tvK31-tvGamma)/(tvGamma-tvAlpha))

############################ Block 2: IM sodium BP ##################

#2-Block for IM sodium BP ; only Sweden; clearance is the SW clearance with code 11. There are two rate constants of absorption noted Ka1 and Ka2. Ka1 is the initial rapid absorption rate that is replaced after a tlag by a slower Ka noted Ka2. “SW” in code is an indicator to SWitch between Ka1 and Ka2

deriv(AaPeniNa = -(SW1PENI*Ka1PeniNa +SW2PENI*Ka2PeniNa ) * AaPeniNa)

deriv(A1PeniNa = (SW1PENI*Ka1PeniNa +SW2PENI*Ka2PeniNa ) * AaPeniNa- Cl * CPeniNa - Cl2 * (CPeniNa - C2PeniNa) - Cl3 * (CPeniNa - C3PeniNa))

deriv(A2PeniNa = Cl2 * (CPeniNa - C2PeniNa))

deriv(A3PeniNa = Cl3 * (CPeniNa - C3PeniNa))

# The statement sequence allows you to change the value of Ka at different times (at tlag);

#double is to declare constants (here SW1 and SW2) that are 0 or 1.

double(SW1PENI,SW2PENI)

sequence{

#at time 0, only Ka1Peni is runing because SW1PENI=1,and Ka2peni =0 because SW2PENI=0

SW1PENI=1

SW2PENI=0

# After tlag1, Ka1Peni is replaced by Ka2peni

sleep(tlagPeni)#e.g 3h

SW1PENI=0

SW2PENI=1

sleep(12-tlagPeni)#we are here at 12h and admin 2 because time is incremental and tlag must be withdrawn

#admin 2 at 12h

#at time 12h,only Ka1Peni is runing because SW1PENI=1,and Ka2peni=0 because SW2PENI=0

SW1PENI=1

SW2PENI=0

#aftfer tlag1,Ka1Peni is replaced by Ka2peni

sleep(tlagPeni)#e.g3hh

SW1PENI=0

SW2PENI=1

sleep(12-tlagPeni)#we are here at 24h and admini 3 because time is incremental ant tlag must be withdrawn

#admin 3 at 24h

SW1PENI=1

SW2PENI=0

#aftfer tlag1, Ka1Peni is replaced by Ka2peni

sleep(tlagPeni)#e.g3hh

SW1PENI=0

SW2PENI=1

sleep(12-tlagPeni)#we are here at 36h and admini4 because time is incremental and tlag must be withdrawn

#admin 4 at 36h

SW1PENI=1

SW2PENI=0

#after tlag1, Ka1Peni is replaced by Ka2peni

sleep(tlagPeni)#e.g3hh

SW1PENI=0

SW2PENI=1

sleep(12-tlagPeni)#we are here at 48h and admini 5 because time is incremental ant tlag must be withdrawn

#at time 0, only Ka1Peni s runing because SW1PENI=1,and Ka2peni=0 because SW2PENI=0

SW1PENI=1

SW2PENI=0

#aftfer tlag1, Ka1Peni is replaced by Ka2peni

sleep(tlagPeni)#e.g3hh

SW1PENI=0

SW2PENI=1

sleep(12-tlagPeni)#we are here at 60h and admini 6 because time is incremental ant tlag must be withdrawn

#at time 0,only Ka1Peni s runing because SW1PENI=1,and Ka2peni =0 because SW2PENI=0

SW1PENI=1

SW2PENI=0

#aftfer tlag1, Ka1Peni is replaced by Ka2peni

sleep(tlagPeni)#e.g3hh

SW1PENI=0

SW2PENI=1

sleep(12-tlagPeni)#we are here at 72h and admini 7 because time is incremental ant tlag must be withdrawn

SW1PENI=1

SW2PENI=0

#aftfer tlag1, Ka1Peni is replaced by Ka2peni

sleep(tlagPeni)#e.g3hh

SW1PENI=0

SW2PENI=1

}

#dosage regimen for sodium Na IM and bioavailability

dosepoint(AaPeniNa,bioavail=(FPeniNa))

CPeniNa = A1PeniNa / V

C2PeniNa = A2PeniNa / V2

C3PeniNa = A3PeniNa / V3

error(CEpsPeniNa = 0.162677174710096)

observe(CObsPeniNa = CPeniNa + CEpsPeniNa * sqrt(1 + CPeniNa^2 * (CMultStdev/sigma())^2))

# To compute individual AUCs after sodium BP administration

deriv(AUC_PeniNa=CPeniNa)

#Only Sweden hence no covariate

stparm(Ka1PeniNa =tvKa1PeniNa*exp(nKa1PeniNA) )

stparm(Ka2PeniNa =tvKa2PeniNa*exp(nKa2PeniNA) )

# lagtime at which Ka1 is replaced by ka2

stparm(tlagPeni =tvtlagPeni*exp(ntlagPeni) )

stparm(FPeniNa=tvFPeniNa*exp(nFPeniNa))

fixef(tvKa1PeniNa = c(,1.02419485674906, ))

fixef(tvKa2PeniNa = c(,0.248123902641013, ))

fixef(tvtlagPeni=c(,0.470979905259535,))

fixef(tvFPeniNa=c(,0.891328516980401,))

# Full OMEGA for sodium BP formulation with a covariance between Ka and F

ranef(block( nFPeniNa,ntlagPeni,nKa1PeniNA,nKa2PeniNA) = c( 0.030733235,

0.00072792607,0.044429534,

0.0021984035,-0.073040728,0.80538569,

0.0059777827,0.0016714216,-0.16919682,0.044433223))

secondary(MAT1_PeniNa=1/tvKa1PeniNa)

secondary(MAT2_PeniNa=1/tvKa2PeniNa)

####### Block 3: Duplocilline (procaine BP + benzathine BP)##########

##3-Duplocilline is a combo of BP benzathine /and procaine BP i.e. of 2 different substances having their own disposition. The Identifiability of the splitting between benzathine BP and procaine BP was likely difficult when analyzed alone during the initial step. In contrast, with the full model that include formulations with only procaine BP , it can be expected a better separation of the two prodrugs of Duplocilline

#Benza1=peni proc from Duplocilline

deriv(AaBenza1 = - KaBenza1 * AaBenza1)

deriv(A1Benza1 = KaBenza1 * AaBenza1- Cl * CBenza1 - Cl2 * (CBenza1 - C2Benza1) - Cl3 * (CBenza1 - C3Benza1))

deriv(A2Benza1 = Cl2 * (CBenza1 - C2Benza1))

deriv(A3Benza1 = Cl3 * (CBenza1 - C3Benza1))

#Dosage procaine BP from Duplocilline (combo)

dosepoint(AaBenza1,bioavail=(FBenza1))

CBenza1 = A1Benza1 / V

C2Benza1 = A2Benza1 / V2

C3Benza1 = A3Benza1 / V3

error(CEpsBenza = 0.00653731719464946)

#Block benzathine from Duplocilline

deriv(AaBenza2 = - KaBenza2 * AaBenza2)

deriv(A1Benza2 = KaBenza2 * AaBenza2 - Cl * CBenza2 - Cl2 * (CBenza2 - C2Benza2) - Cl3 * (CBenza2 - C3Benza2))

deriv(A2Benza2 = Cl2 * (CBenza2 - C2Benza2))

deriv(A3Benza2 = Cl3 * (CBenza2 - C3Benza2))

# Dosage regimen for Pen benzathine and F (0 to about 1)

dosepoint(AaBenza2,bioavail=(FBenza2))

CBenza2 = A1Benza2 / V

C2Benza2 = A2Benza2 / V2

C3Benza2 = A3Benza2 / V3

# What is measured and observed is the summation of BP released by the 2 products of the combo

CBenza=CBenza1+CBenza2

observe(CObsBenza = CBenza +CEpsBenza * sqrt(1 + CBenza^2 * (CMultStdev/sigma())^2))

# To compute individual AUCs of each product of the combo after Duplocilline administration

deriv(AUC_Benza1=CBenza1)

deriv(AUC_Benza2=CBenza2)

deriv(AUCBenzaTOT=CBenza1+CBenza2)

#only French data for IM,hence no covariate for this parameters

stparm(KaBenza1=tvKaBenza1*exp(nKaBenza1))

stparm(KaBenza2 = tvKaBenza2 * exp(nKaBenza2))

stparm(FBenza2=ilogit(tvFBenza2 +nFBenza2))

stparm(FBenza1=ilogit(tvFBenza1 +nFBenza1))

fixef(tvKaBenza1 = c(,0.0827110143014943, ))

fixef(tvFBenza1=c(,1.53153195755327,))

fixef(tvKaBenza2 = c(,0.00935419980483117, ))

fixef(tvFBenza2=c(,10.9421855761952,))

#OMEGA for Duplocilline formulation with a covariance between Ka and F

ranef(block( nKaBenza1,nKaBenza2,nFBenza1,nFBenza2) = c(0.12663874,0.1798759, 0.61633102,0.081958913,0.77856969,2.1630137,0.20130889,0.58867596,1.3440868,1.160584))

#computation of the bioavailability of BP from the two component of Duplocilline from ilogit estimates

#Ben1 is procaine BP and Ben2 is benzathine BP

secondary(Bioavailability_Ben1=exp(tvFBenza1)/(1+exp(tvFBenza1)))

secondary(Bioavailability_Ben2=exp(tvFBenza2)/(1+exp(tvFBenza2)))

secondary(MAT_Benza1=1/tvKaBenza1)

secondary(MAT_Benza2=1/tvKaBenza2)

############### Block 4 BP-PROC ###################

deriv(AaPROC = - KaPROC * AaPROC)

# with clearance coded for each countries as in block 1

deriv(A1PROC = KaPROC * AaPROC - Cl * CPROC - Cl2 * (CPROC - C2PROC) - Cl3 * (CPROC - C3PROC))

deriv(A2PROC = Cl2 * (CPROC - C2PROC))

deriv(A3PROC = Cl3 * (CPROC - C3PROC))

dosepoint(AaPROC,bioavail=(FPROC))

CPROC = A1PROC / V

C2PROC = A2PROC / V2

C3PROC = A3PROC / V3

error(CEpsPROC = 0.00457902506048033)

observe(CObsPROC = CPROC + CEpsPROC * sqrt(1 + CPROC^2 * (CMultStdev/sigma())^2))

# to compute individual AUCs after procaine BP administration

deriv(AUC_PROC=CPROC)

# F for procaine BP is coded with the covariate nation: 0=Fr, 11=SW,2=USA1; 3=Japan

stparm(FPROC=ilogit(tvFPROC* exp(dFNationcode11*(Nationcode==11)) * exp(dFNationcode2*(Nationcode==2)) * exp(dFNationcode3*(Nationcode==3)) +nFPROC))

# Ka for procaine BP is coded with the covariate nation: 0=Fr, 2=USA1; 3=Japan 11=SW2

stparm(KaPROC = tvKaPROC * exp(dKadNationcode11*(Nationcode==11)) * exp(dKadNationcode2*(Nationcode==2)) * exp(dKadNationcode3*(Nationcode==3)) * exp(nKaPROC))

fixef(tvKaPROC = c(,0.046594811352403, ))

fixef(tvFPROC=c(,7.83103159739662,))

# Covariate for Ka for procaine BP: nation: 0=Fr, 11=SW2,2=USA1 4=Japan

fixef(dKadNationcode11(enable=c(0))(freeze) = c(, 0.0390078308660437, ))

fixef(dKadNationcode2(enable=c(0))(freeze)= c(, -2.37155662564783, ))

fixef(dKadNationcode3(enable=c(0))(freeze) = c(, -0.00427442230486608, ))

# Covariate for F for procaine BP: nation: 0=Fr, 11=SW2,2=USA1 3=Japan

fixef(dFNationcode11(enable=c(0))= c(, -0.00177886964815447, ))

fixef(dFNationcode2(enable=c(0))(freeze) = c(, 0.0517205803762012, ))

fixef(dFNationcode3(enable=c(0)) (freeze)= c(, 0.422679139787995, ))

#Full Omega for Ka and F

ranef(block(nKaPROC,nFPROC)=c(0.10963699,0.52361095,3.6624274))

#computation of the bioavailability of procaine BP from ilogit estimates

secondary(Bioavailability_ProcFr=exp(tvFPROC)/(1+exp(tvFPROC)))

# Bioavailability SW2

bioSW11=exp(tvFPROC*exp(dFNationcode11))

secondary(Bioavailability_procSW=bioSW11/(1+bioSW11))

# Bioavailability USA1

bioUS=exp(tvFPROC*exp(dFNationcode2))

secondary(Bioavailability_procUS=bioUS/(1+bioUS))

# Bioavailability Japan

bioJP=exp(tvFPROC*exp(dFNationcode3))

secondary(Bioavailability_procJP=bioJP/(1+bioJP))

#secondary Ka

secondary(KaSW_PROc=tvKaPROC*exp(dKadNationcode11))

secondary(KaUS1_PROc=tvKaPROC*exp(dKadNationcode2))

secondary(KaJP_PROc=tvKaPROC*exp(dKadNationcode3))

secondary(MAT_PenPROC_France=1/tvKaPROC)

secondary(MAT_KaSW_PROc=1/(tvKaPROC*exp(dKadNationcode11)))

secondary(MAT_KaUS1_PROc=1/(tvKaPROC*exp(dKadNationcode2)))

secondary(MAT_KaJP_PROc=1/(tvKaPROC*exp(dKadNationcode3)))

############### block 5 penethamate #####################

#5- block for penethamate; there was 3 sites of administration with evident differences between them in terms of rate constant (Ka_rapid =KaPenethamate1 and Ka_slow) explaining that I model each site separately with two sequential rate constants of absorption and a delay between them.

#site 1

deriv(AaPenethamate1 = - (SW1A*KaPenethamate1+SW1B*KaPenethamate_slow1) * AaPenethamate1)

deriv(A1Penethamate1 = (SW1A*KaPenethamate1+SW1B*KaPenethamate_slow1) * AaPenethamate1 - Cl * CPenethamate1 - Cl2 * (CPenethamate1 - C2Penethamate1) - Cl3 * (CPenethamate1 - C3Penethamate1))

deriv(A2Penethamate1 = Cl2 * (CPenethamate1 - C2Penethamate1))

deriv(A3Penethamate1 = Cl3 * (CPenethamate1 - C3Penethamate1))

#dose for site 1

dosepoint(AaPenethamate1,bioavail=(FPenethamate))

#contribution of site 1 to plasma concentration

CPenethamate1 = A1Penethamate1 / V

C2Penethamate1 = A2Penethamate1 / V2

C3Penethamate1 = A3Penethamate1 / V3

#site 2

deriv(AaPenethamate2 = - (SW2A*KaPenethamate2+SW2B*KaPenethamate_slow2) * AaPenethamate2)

deriv(A1Penethamate2 = (SW2A*KaPenethamate2+SW2B*KaPenethamate_slow2) * AaPenethamate2 - Cl * CPenethamate2 - Cl2 * (CPenethamate2 - C2Penethamate2) - Cl3 * (CPenethamate2 - C3Penethamate2))

deriv(A2Penethamate2 = Cl2 * (CPenethamate2 - C2Penethamate2))

deriv(A3Penethamate2 = Cl3 * (CPenethamate2 - C3Penethamate2))

dosepoint(AaPenethamate2,bioavail=(FPenethamate))

CPenethamate2 = A1Penethamate2 / V

C2Penethamate2 = A2Penethamate2 / V2

C3Penethamate2 = A3Penethamate2 / V3

#site 3

deriv(AaPenethamate3 = - (SW3A*KaPenethamate3+SW3B*KaPenethamate_slow3) * AaPenethamate3)

deriv(A1Penethamate3 = (SW3A*KaPenethamate3+SW3B*KaPenethamate_slow3) * AaPenethamate3 - Cl * CPenethamate3 - Cl2 * (CPenethamate3 - C2Penethamate3) - Cl3 * (CPenethamate3 - C3Penethamate3))

deriv(A2Penethamate3 = Cl2 * (CPenethamate3 - C2Penethamate3))

deriv(A3Penethamate3 = Cl3 * (CPenethamate3 - C3Penethamate3))

dosepoint(AaPenethamate3,bioavail=(FPenethamate))

CPenethamate3 = A1Penethamate3 / V

C2Penethamate3 = A2Penethamate3 / V2

C3Penethamate3 = A3Penethamate3 / V3

error(CEpsPenethamate = 0.00806824116414487)

# give Cobs (IPRED) by adding the concentration resulting from site1,2 and 3

CPenethamate=CPenethamate1+CPenethamate2+CPenethamate3

# to compute AUCs with the contribution of each site

deriv(AUC_Penethamate1=CPenethamate1)

deriv(AUC_Penethamate2=CPenethamate2)

deriv(AUC_Penethamate3=CPenethamate3)

deriv(AUC_PenethamateTOT=CPenethamate1+CPenethamate2+CPenethamate3)

# give Cobs by adding the concentrations of site1,2 and 3+ error

observe(CObsPenethamate = CPenethamate+ CEpsPenethamate * sqrt(1 + CPenethamate^2 * (CMultStdev/sigma())^2))

# this is for the initial/rapid rate constants of absorption

stparm(KaPenethamate1=tvKaPenethamate1*exp(nKaPenethamate1))

stparm(KaPenethamate2=tvKaPenethamate2*exp(nKaPenethamate2))

stparm(KaPenethamate3=tvKaPenethamate3*exp(nKaPenethamate3))

#this is for the late/slow rate constant of absorption

stparm(KaPenethamate_slow1=tvKaPenethamate_slow1*exp(nKaPenethamate_slow1))

stparm(KaPenethamate_slow2=tvKaPenethamate_slow2*exp(nKaPenethamate_slow2))

stparm(KaPenethamate_slow3=tvKaPenethamate_slow3*exp(nKaPenethamate_slow3))

# The statement sequence allows you to change the value of Ka at different times (at tlag); the advantage of setting up 3 different sequences rather than just one is to be able to manage the time of each administration site separately, which could not be done by a single statement insofar as the times are cumulative within the same sequence (or requires a difficult coding and even sometimes impossible as for the case where tlag1 would be greater than the time of the dose 2 of the site 2

#Double is to declare constants (here SW1 and SW2) that are 0 or 1.

double(SW1A,SW1B)

sequence{

#at time 0, only KaPenethamate1 is running because SW1A=1,and Kapenethamate_slow1=0 because SW1B=0

SW1A=1

SW1B=0

#After tlag1 Kpenthamate1 is replaces by Penethamate_slow1 that is the second rate of absorption for site 1

sleep(tlag1)#e.g15h

SW1A=0

SW2A=1

}

#this is for site 2

double(SW2A,SW2B)

sequence{

#at time 0, neither KaPenethamate2 or Kapenethamate_slow2 are runing because time of administration i 24h hence SW2A=SW2B=0, intially

SW2A=0

SW2B=0

#after 24h, SW2A take value of 1 and Kapenethamate2 start running up to Tlag2 where it is replaced by Kapenethamate_slow2.

sleep(24)

SW2A=1

sleep(tlag2)#e.g15h

SW2A=0

SW2B=1

}

#for site 3

double(SW3A,SW3B)

sequence{

#at time 0, no absorption because time of administration is 48h,hence SW3A=SW3B=0

SW3A=0

SW3B=0

#aftfer 48h, Kapenethamate3 is runing up to Tlag3 when it is replaced by Kapenethamate_slow3

sleep(48)

SW3A=1

sleep(tlag3)#e.g15h

SW3A=0

SW3B=1

}

stparm(tlag1=tvtlag1)

stparm(tlag2=tvtlag2)

stparm(tlag3=tvtlag3)

# I used a common value of F for the 3 sites and the ilogit function to avoid calculating an F> 1

stparm(FPenethamate=ilogit(tvFPenethamate+nFPenethamate))

#initial rate constant for Ka for penethamate

fixef(tvKaPenethamate1 = c(,0.0350071439943249, ))

fixef(tvKaPenethamate2 = c(,0.0223223860881139, ))

fixef(tvKaPenethamate3 = c(,0.0151705494676296, ))

#late rate constant of absorbtion for penethamate

fixef(tvKaPenethamate_slow1= c(,0.0141803796444364, ))

fixef(tvKaPenethamate_slow2 = c(,0.108333675324903, ))

fixef(tvKaPenethamate_slow3 = c(,0.00162233822089588, ))

secondary(MAT_Penethamate1=1/tvKaPenethamate1)

secondary(MAT_Penethamate2=1/tvKaPenethamate2)

secondary(MAT_Penethamate3=1/tvKaPenethamate3)

fixef(tvFPenethamate=c(,0.788807915876975,))

#These are the delays after the first administration

fixef(tvtlag1=c(,70.6482526955672,))

#Delay after 24h,that is the time of administration

fixef(tvtlag2=c(,27.2167070491644,))

#Delay after 48h, that is the time of administration

fixef(tvtlag3=c(,42.3487955074074,))

#Full OMEGA for PENETHAMATE formulation with a covariance between Ka and F

ranef(block(nKaPenethamate1,nKaPenethamate_slow1)(freeze)=c(1.2160073,0.24768147,0.050500768))

ranef(block(nKaPenethamate2,nKaPenethamate_slow2)(freeze)=c(0.094452959,0.044713983,0.021186634))

ranef(block(nKaPenethamate3,nKaPenethamate_slow3)(freeze)=c(3.1459704,0.30982059,0.030569386))

#diagonal OMEGA for F

ranef(diag(nFPenethamate)=c(0.0047176976))

#bioavailability of penethamate

secondary(Bioavailabity_Penethamate=exp(tvFPenethamate)/(1+exp(tvFPenethamate)))

secondary(tvVarea=tvCl/tvGamma)

##################### block Monte Carlo Simulation ##############

#to declare MIC of interest

MIC_00625=0.0625

MIC_010=0.1

MIC_0125=0.125

MIC_025=0.25

MIC_0375=0.375

MIC_05=0.5

MIC_1=1

MIC_2=2

# to compute AUC/MIC for pen proc see line 254 with deriv(AUC_PROC=CPROC); considering fu=0.4 is equivalent to divide the AUC by the targeted MIC divided by 0.4

AUC_PROC_MIC_0_0625=AUC_PROC/0.1563

AUC_PROC_MIC_0_100=AUC_PROC/0.25

AUC_PROC_MIC_0_125=AUC_PROC/0.3125

AUC_PROC_MIC_0_250=AUC_PROC/0.6250

AUC_PROC_MIC_0_0375=AUC_PROC/0.9375

AUC_PROC_MIC_05=AUC_PROC/1.25

AUC_PROC_MIC_1=AUC_PROC/2.5

AUC_PROC_MIC_2=AUC_PROC/5

# to compute AUC/MIC for penethamate see line 356 with deriv(AUC_PenethamateTOT=CPenethamate1+CPenethamate2+CPenethamate3); considering fu=0.4 is equivalent to divide AUC by twice the targeted MIC

AUC_penthamate_MIC_0_0625=AUC_PenethamateTOT/0.1563

AUC_penthamate_MIC_0_0100=AUC_PenethamateTOT/0.25

AUC_penthamate_MIC_0_0125=AUC_PenethamateTOT/0.3125

AUC_penthamate_MIC_0_250=AUC_PenethamateTOT/0.6250

AUC_penthamate_MIC_0_0375=AUC_PenethamateTOT/0.9375

AUC_penthamate_MIC_0_500=AUC_PenethamateTOT/1.250

AUC_penthamate_MIC_1=AUC_PenethamateTOT/2.5

AUC_penthamate_MIC_2=AUC_PenethamateTOT/5

# to compute AUC/MIC for duplocilline (peni_proc+peni_benzathine see line 205 deriv(AUCBenzaTOT=CBenza1+CBenza2) considering fu=0.4 is equivalent to divide AUC by twice the targeted MIC

AUC_Duplo_MIC_0_0625=AUCBenzaTOT/0.1563

AUC_Duplo_MIC_0_0100=AUCBenzaTOT/0.25

AUC_Duplo_MIC_0_0125=AUCBenzaTOT/0.31250

AUC_Duplo_MIC_0_0250=AUCBenzaTOT/0.62500

AUC_Duplo_MIC_0_0375=AUCBenzaTOT/0.9375

AUC_Duplo_MIC_0_500=AUCBenzaTOT/1.250

AUC_Duplo_MIC_1=AUCBenzaTOT/2.5

AUC_Duplo_MIC_2=AUCBenzaTOT/5

#*****Computation of time above MIC ####################

########## computation for procaine BP ##############

#this expression compare CPROC (IPRED of procaine BP concentration) to the MIC in question; if CPROC is lower than MIC, then the returned value is 0, otherwise Phoenix return the actual time . In other words when tested Conc is lower than CPROC, the column is filled with 0 and as CPROC became higher than MIC, the column is filled with the corresponding actual time

#We have to compute the duration for which T>MIC=0.125;0.25.... by coding 0=no and 1=yes allowing to have a series of 1 that we can integrate to give the cumulated time over which the column contains 1 i.e fT>MIC ; here 0.4 is fu

Time_Above_MIC00625_codeCPROC =(0.4*CPROC<0.0625?0:1)

Time_Above_MIC0100_codeCPROC =(0.4*CPROC<0.10?0:1)

Time_Above_MIC0125_codeCPROC =(0.4*CPROC<0.125?0:1)

Time_Above_MIC0375_codeCPROC =(0.4*CPROC<0.375?0:1)

Time_Above_MIC0250_codeCPROC=(0.4*CPROC<0.250?0:1)

Time_Above_MIC0500_codeCPROC=(0.4*CPROC<0.50?0:1)

Time_Above_MIC1_codeCPROC=(0.4*CPROC<1.00?0:1)

Time_Above_MIC2_codeCPROC=(0.4*CPROC<2.00?0:1)

# To compute T>MIC consist to now computing“"deriv”" of code to integrate the series of 1 i.e compute time above code 1

deriv(T_above_00625PROC=Time_Above_MIC00625_codeCPROC)

deriv(T_above_0100PROC=Time_Above_MIC0100_codeCPROC)

deriv(T_above_0125PROC=Time_Above_MIC0125_codeCPROC)

deriv(T_above_0250PROC=Time_Above_MIC0250_codeCPROC)

deriv(T_above_0375PROC=Time_Above_MIC0375_codeCPROC)

deriv(T_above_0500PROC=Time_Above_MIC0500_codeCPROC)

deriv(T_above_10PROC=Time_Above_MIC1_codeCPROC)

deriv(T_above_2PROC=Time_Above_MIC2_codeCPROC)

########## computation of T>MIC for penethamate n##############

#We have to compute the duration for which T>MIC=0.125 by coding 0=no and 1=yes allowing to have a series of 1 that we can integrate to give the cumulated time over which the column contains 1 i.e fT>MIC ; here 0.4 is fu

Time_Above_MIC00625_codePenethamate =(0.4*CPenethamate<0.0625?0:1)

Time_Above_MIC0100_codePenethamate =(0.4*CPenethamate<0.10?0:1)

Time_Above_MIC0125_codePenethamate =(0.4*CPenethamate<0.125?0:1)

Time_Above_MIC0250_codePenethamate=(0.4*CPenethamate<0.250?0:1)

Time_Above_MIC0375_codePenethamate =(0.4*CPenethamate<0.375?0:1)

Time_Above_MIC0500_codePenethamate=(0.4*CPenethamate<0.50?0:1)

Time_Above_MIC1_codePenethamate=(0.4*CPenethamate<1.00?0:1)

Time_Above_MIC2_codePenethamate=(0.4*CPenethamate<2.00?0:1)

# to compute T>MIC consist to now computing“"deriv”" of code integrating the series of 1 i.e compute time above code 1

deriv(T_above_00625Penethamate=Time_Above_MIC00625_codePenethamate)

deriv(T_above_0100Penethamate=Time_Above_MIC0100_codePenethamate)

deriv(T_above_0125Penethamate=Time_Above_MIC0125_codePenethamate)

deriv(T_above_0250Penethamate=Time_Above_MIC0250_codePenethamate)

deriv(T_above_0375Penethamate=Time_Above_MIC0375_codePenethamate)

deriv(T_above_0500Penethamate=Time_Above_MIC0500_codePenethamate)

deriv(T_above_1Penethamate=Time_Above_MIC1_codePenethamate)

deriv(T_above_2Penethamate=Time_Above_MIC2_codePenethamate)

#### computation of T>MIC for BP Duplocilline (benzathine +procaine)##############

#We have to compute the duration for which T>MIC=0.125 by coding 0=no and 1=yes allowing to have a series of 1 that we can integrate to give the cumulated time over which the column contains 1 i.e fT>MIC ; here 0.4 is fu

# #code 0 or 1 if < or 0.125 here

Time_Above_MIC00625_codeCBenza=(0.4*CBenza<0.0625?0:1)

Time_Above_MIC0100_codeCBenza=(0.4*CBenza<0.100?0:1)

Time_Above_MIC0125_codeCBenza=(0.4*CBenza<0.125?0:1)

Time_Above_MIC0250_codeCBenza=(0.4*CBenza<0.250?0:1)

Time_Above_MIC0375_codeCBenza=(0.4*CBenza<0.375?0:1)

Time_Above_MIC0500_codeCBenza=(0.4*CBenza<0.50?0:1)

Time_Above_MIC1_codeCBenza=(0.4*CBenza<1.00?0:1)

Time_Above_MIC2_codeCBenza=(0.4*CBenza<2.00?0:1)

# to compute T>MIC consist to now computing“"deriv”" of code integrating the series of 1 i.e compute time above code 1

deriv(T_above_00625Benza=Time_Above_MIC00625_codeCBenza)

deriv(T_above_0100Benza=Time_Above_MIC0100_codeCBenza)

deriv(T_above_0125Benza=Time_Above_MIC0125_codeCBenza)

deriv(T_above_0250Benza=Time_Above_MIC0250_codeCBenza)

deriv(T_above_0375Benza=Time_Above_MIC0375_codeCBenza)

deriv(T_above_0500Benza=Time_Above_MIC0500_codeCBenza)

deriv(T_above_1Benza=Time_Above_MIC1_codeCBenza)

deriv(T_above_2Benza=Time_Above_MIC2_codeCBenza)

########## computation fo sodium BP IM ##############

#We have to compute the duration for which T>MIC=0.125 by coding 0=no and 1=yes allowing to have a series of 1 that we can integrate to give the cumulated time over which the column contains 1 i.e fT>MIC ; here 0.4 is fu

Time_Above_MIC00625_codeCPeniNa=(0.4*CPeniNa<0.0625?0:1)

Time_Above_MIC0100_codeCPeniNa=(0.4*CPeniNa<0.100?0:1)

Time_Above_MIC0125_codeCPeniNa=(0.4*CPeniNa<0.125?0:1)

Time_Above_MIC0250_codeCPeniNa=(0.4*CPeniNa<0.250?0:1)

Time_Above_MIC0375_codeCPeniNa=(0.4*CPeniNa<0.375?0:1)

Time_Above_MIC0500_codeCPeniNa=(0.4*CPeniNa<0.50?0:1)

Time_Above_MIC1_codeCPeniNa=(0.4*CPeniNa<1.00?0:1)

Time_Above_MIC2_codeCPeniNa=(0.4*CPeniNa<2.00?0:1)

# to compute T>MIC consist to now computing“"deriv”" of code integrating the series of 1 i.e compute time above code 1

deriv(T_above_00625PeniNA_IM=Time_Above_MIC00625_codeCPeniNa)

deriv(T_above_0100PeniNA_IM=Time_Above_MIC0100_codeCPeniNa)

deriv(T_above_0125PeniNA_IM=Time_Above_MIC0125_codeCPeniNa)

deriv(T_above_0250PeniNA_IM=Time_Above_MIC0250_codeCPeniNa)

deriv(T_above_0375PeniNA_IM=Time_Above_MIC0375_codeCPeniNa)

deriv(T_above_0500PeniNA_IM=Time_Above_MIC0500_codeCPeniNa)

deriv(T_above_1PeniNA_IM=Time_Above_MIC1_codeCPeniNa)

deriv(T_above_2PeniNA_IM=Time_Above_MIC2_codeCPeniNa)

##### computation of sodium or potassium BP IV infusion ##############

#We have to compute the duration for which T>MIC=0.125 by coding 0=no and 1=yes allowing to have a series of 1 series of 1 that we can integrate to give the cumulated time over which the column contains 1 i.e fT>MIC ; here 0.4 is fu

Time_Above_MIC00625_codeIV=(0.4*CIV<0.0625?0:1)

Time_Above_MIC0100_codeIV=(0.4*CIV<0.100?0:1)

Time_Above_MIC0125_codeIV=(0.4*CIV<0.125?0:1)

Time_Above_MIC0250_codeIV=(0.4*CIV<0.250?0:1)

Time_Above_MIC0375_codeIV=(0.4*CIV<0.375?0:1)

Time_Above_MIC0500_codeIV=(0.4*CIV<0.50?0:1)

Time_Above_MIC1_codeIV=(0.4*CIV<1.00?0:1)

Time_Above_MIC2_codeIV=(0.4*CIV<2.00?0:1)

# to compute T>Mic consist to now computing“"deriv”" of code integrating the series of 1 i.e compute time above code 1

deriv(T_above_00625_IV_INF=Time_Above_MIC00625_codeIV)

deriv(T_above_0100_IV_INF=Time_Above_MIC0100_codeIV)

deriv(T_above_0125_IV_INF=Time_Above_MIC0125_codeIV)

deriv(T_above_0250_IV_INF=Time_Above_MIC0250_codeIV)

deriv(T_above_0375_IV_INF=Time_Above_MIC0375_codeIV)

deriv(T_above_0500_IV_INF=Time_Above_MIC0500_codeIV)

deriv(T_above_1_IV_INF=Time_Above_MIC1_codeIV)

deriv(T_above_2_IV_INF=Time_Above_MIC2_codeIV) }

#End

# Supplementary Figures and Tables

Supplementary Figure S1**.** Semi-logarithmic spaghetti plots of the disposition curves of BP after administration of sodium or potassium BP by IV route for a scaled dose of 1 mg/kg. Black=French; Blue=USA1; Magenta=Swedish, Red=UAS2). Left panel from 0 to 10h and right panel from 0 to 1 h post administration.

*Visual inspection of figure S1A suggests that the French, USA1 and USA2 data are rather similar but that 3 of the 4 Swedish horses are clearly above the French and USA horses (left panel) but the last Swedish horse (right panel) had the lowest initial plasma concentration profile.*

**
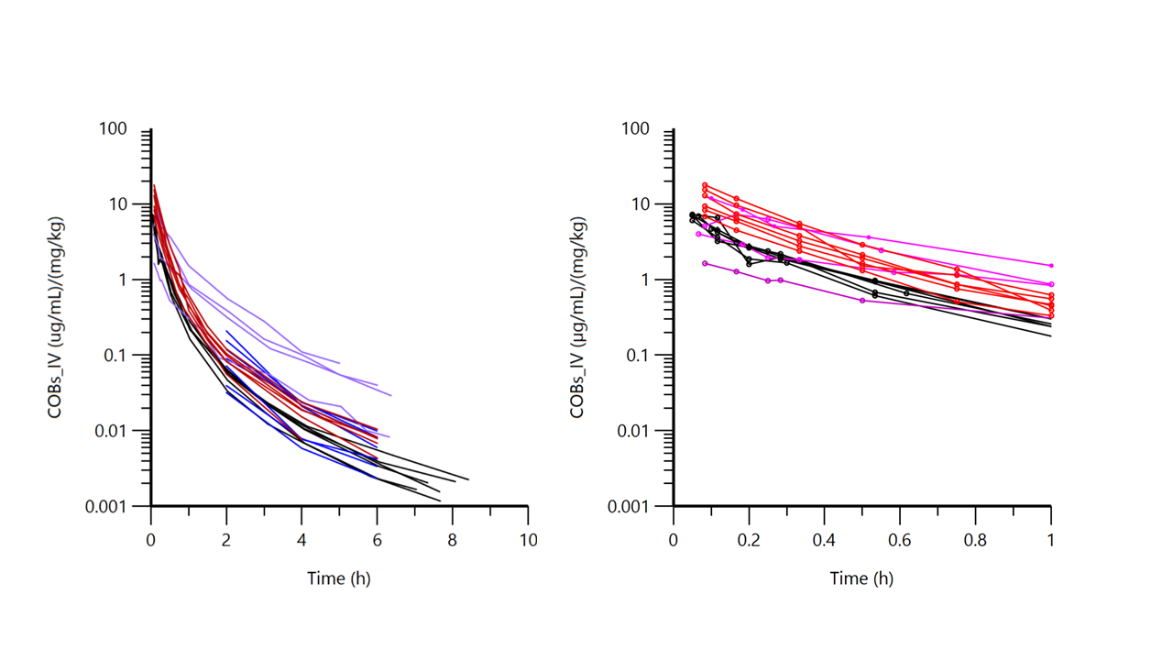
**

Supplementary **Figure S2.** Semi-logarithmic spaghetti plots of the disposition curves of BP after administration of different formulations of procaine BP (Black=French, Red=Japanese; Blue=USA1, Magenta=Swedish with only two times post administration) by IM route for a scaled BP dose of 1 mg/kg.


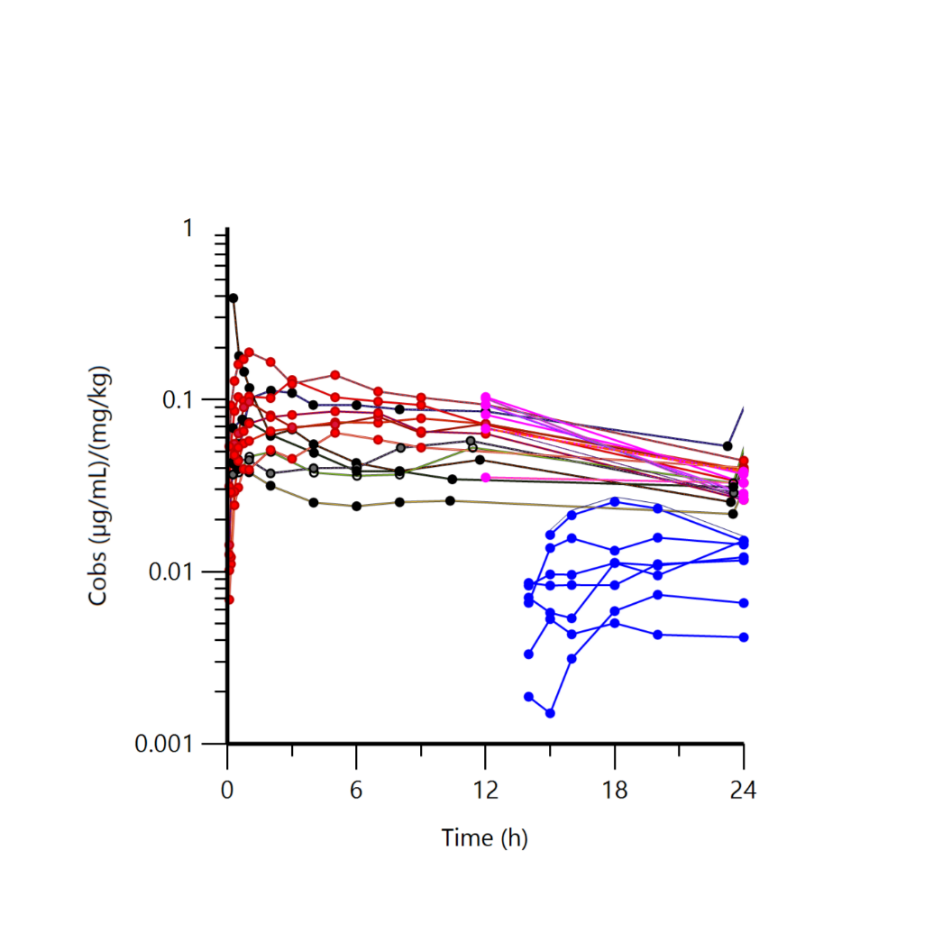


Supplementary **Figure S3.** Semi-logarithmic spaghetti plots of the disposition curves of BP into the 6 French horses for three IM formulations at the recommended SPC dosing regimen. Black curve: procaine BP (Depocilline®) at 10 mg/kg administered 3 times at 24-hour intervals. Red curves: a mixture of procaine BP and benzathine BP (Duplocilline ®) administered twice at 48h interval at 12.5 mg/kg. Blue curves: penethamate hydriodide (Penetavet®) at a dose of about 11.91 mg/kg at Day 1 followed by 5.96 mg/kg at Day 2 and Day 3.

*Visual inspection of figures S1C indicates that the BP exposure was not equivalent for these three formulations of BP administration with a more rapid decay for penethamate and a more higher BP plasma concentration following the three administration of procaine BP than after the two administrations of a combination of procaine and benzathine BP. This figure also indicate that the terminal decay was slower with the combination of procaine and benzathine BP than after an administration of only procaine BP.*


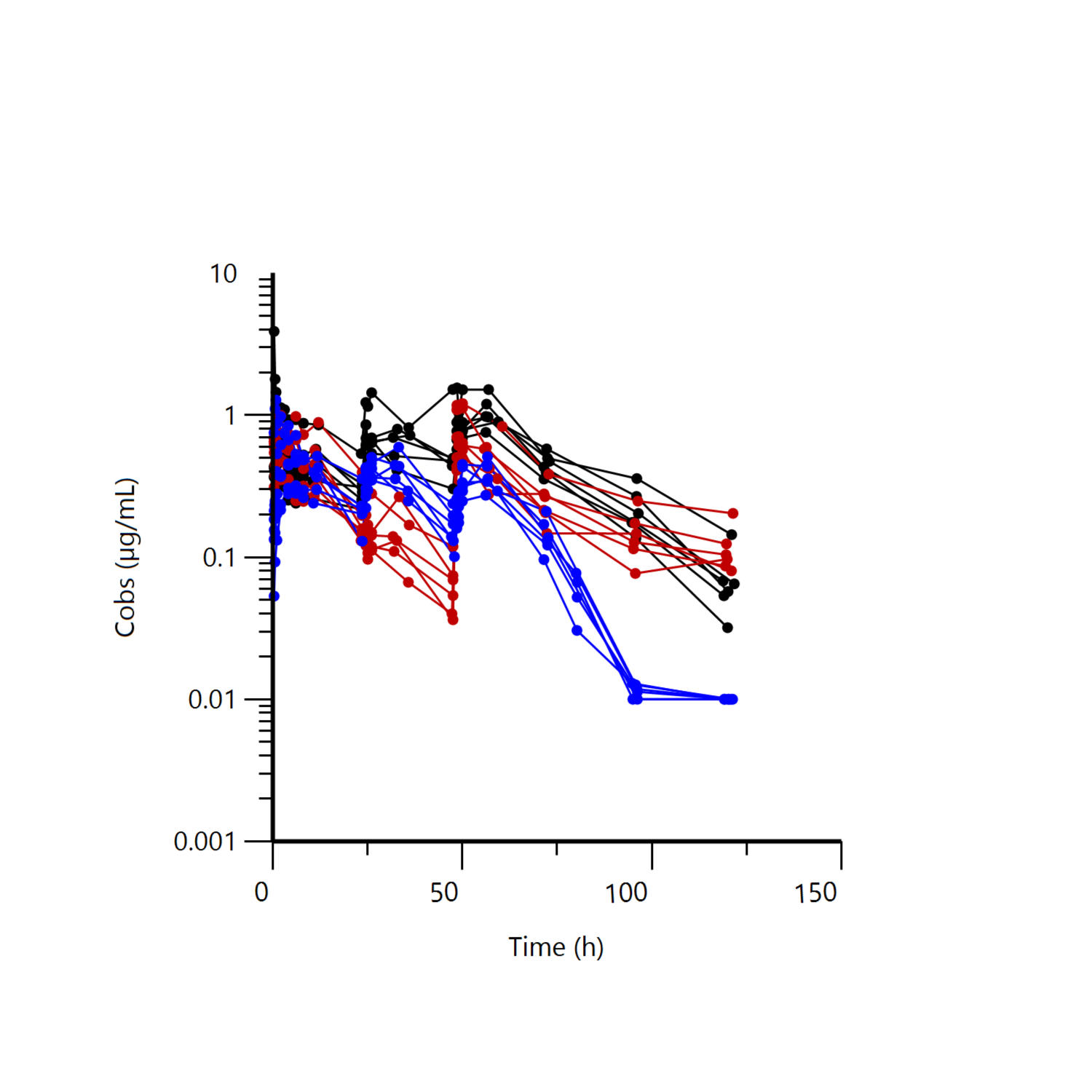


# Supplementary Figure S4: Goodness-of-fit (GOF) plots for the IV data supporting the 3-comparment structural model, the exponential model for the random component and the additive plus multiplicative model for the error submodel that were used to analyses all the data.

**Figure S4A: DV vs PRED**. Plot of the dependent variable i.e. of plasma BP concentrations (µg/mL) versus population predicted plasma BP concentrations (PRED) (no random component). The plot shows observed vs. fitted values of the model function. Ideally, they should fall close to the line of unity y=x. Arithmetic scale (left) and logarithmic scale (right).

*For the arithmetic scale, data are evenly distributed about the line of identity, indicating no major bias in the population component of the model. The log10 scale does not indicate any misfit for the smallest values.*

**Figure S4B: Plot of the dependent variable i.e. of observed plasma BP concentrations (µg/mL) versus individual predicted plasma BP values (IPRED).** Individual predictions are obtained by setting random effects to the 'post hoc' or empirical Bayesian estimate of the random effects for the individual from which the plasma concentration observation was made. Thus, the plot shows observed vs fitted values of the model function. Ideally, they should fall close to the line of unity y=x. Arithmetic scale (left) and logarithmic scale (right).

*For both the arithmetic and log10 scales, data are evenly distributed about the line of identity, indicating no major bias in the random component of the model.*

**Figure S4C: CWRES vs. Time after administration.** Plot of CWRES (conditional weighted residuals), a proposed replacement for the classical WRES (weighted residuals) goodness of fit statistic, against IVAR (time). Values of CWRES should be approximately N(0,1) and hence concentrated between y=-2 and y=+2. Values significantly above 3 or below -3 are suspect and may indicate a lack of fit and/or model misspecification.

*Inspection of the figure shows that data are evenly distributed around zero (see the average trends as given by the blue line that must be as close as possible of the horizontal line), indicating no bias in the structural model. Red and blue curves are loess regression curves (LOESS (LOcally wEighted Scatter plot Smoothing)). The blue curve takes into account the sign of the residuals (positive or negative) while the red curve and its reflection only consider absolute value of residuals. Ideally, the blue line should be at 0 and the red line (with its negative reflection) should not show any fanning. Fanning indicates room for improving the distribution of residuals.*

**Figure S4D: CWRES QQ plot.** Quantile-quantile (QQ) plot for CWRES. If the components of CWRES are well described by a normal distribution, plotted values will fall roughly along a straight line of unity y=x. Significant deviations from normality, particularly in the tails of the distribution, can be seen by deviations from this line. This plot can be considered as a diagnostic of model miss-specification.

**Figure S4E: Plot (latticed by individual) of dependent variable (plasma BP concentration, black circles) and individual predicted curve (black line) vs Time (hours) after dose administration.** Plots1 to 6: French horses, Plots 13 to 19: USA1 horses; Plots 20 to 23: Swedish horses; Plots 33 to 37: USA2 horses.

*Inspection of these different plots suggest a good fitting of all the data with the selected 3-compartmental model.*

**French horses**

**
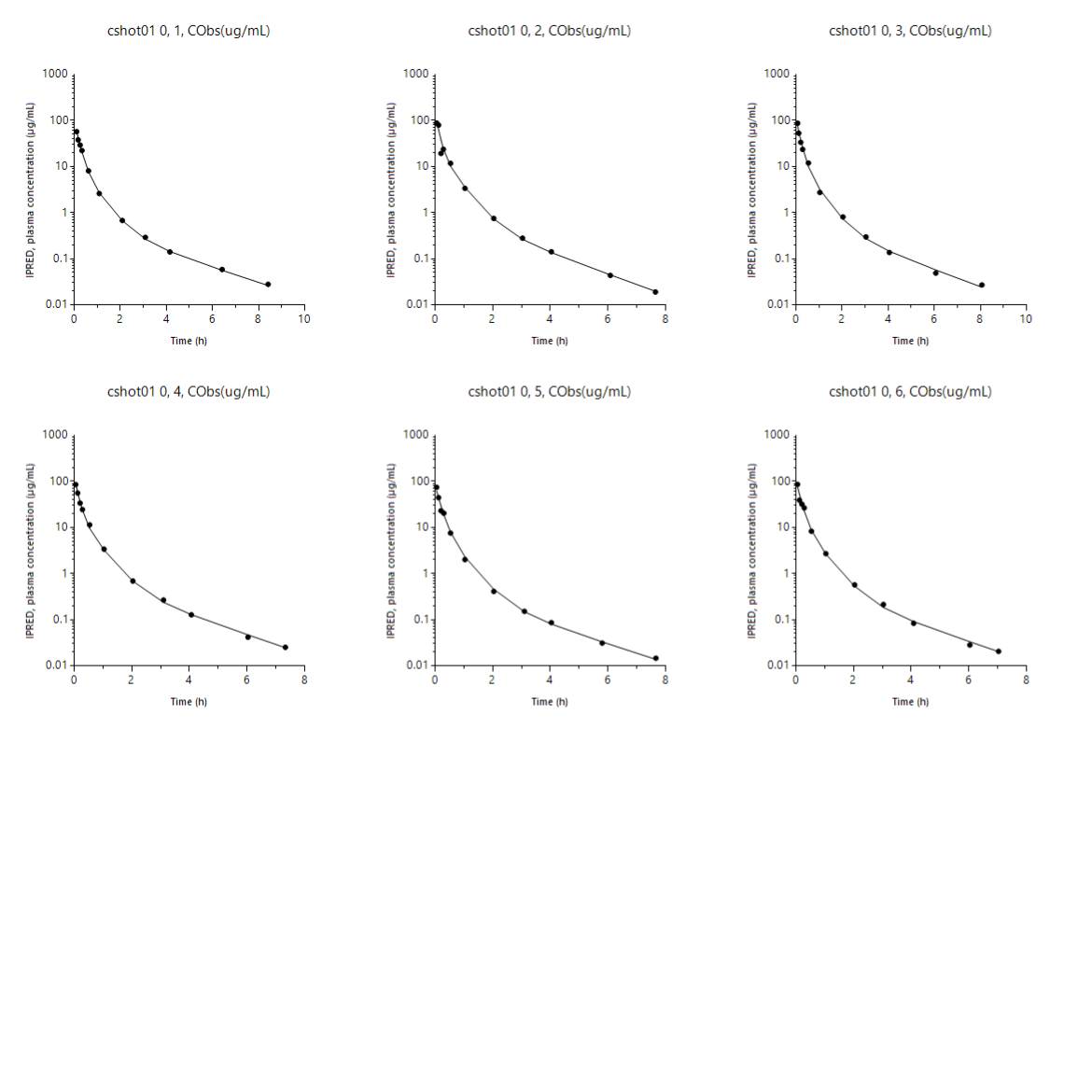
**

**USA1 horses**

**
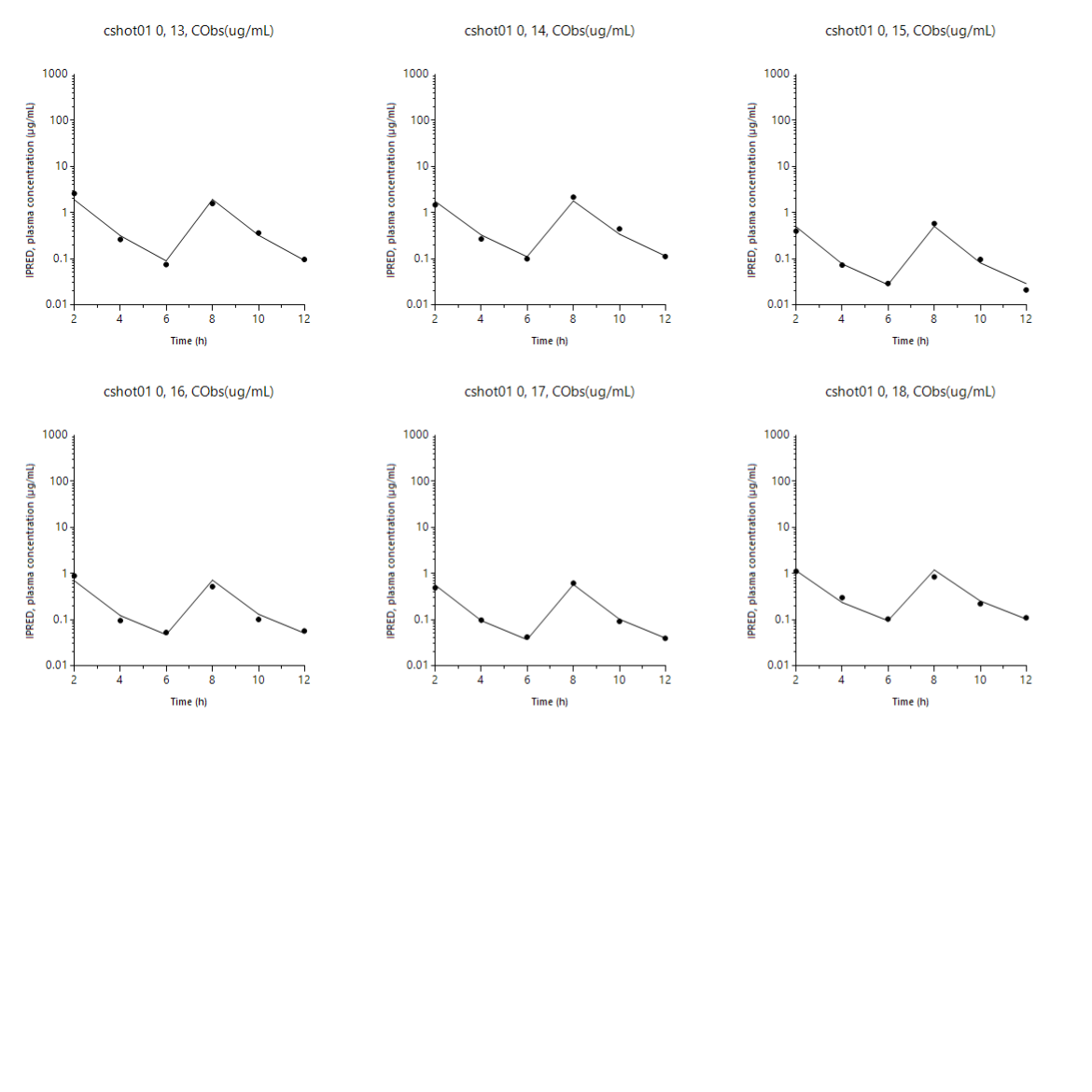
**

**
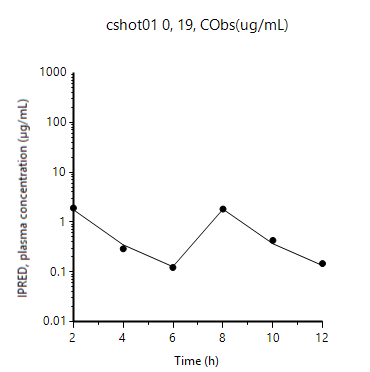
**

**Swedish horses**

**
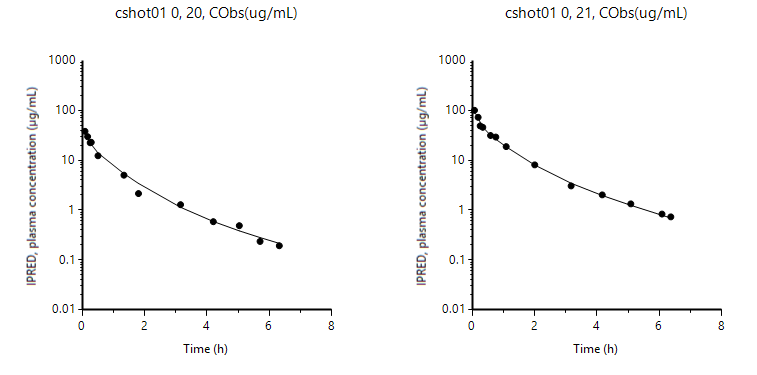
**

**
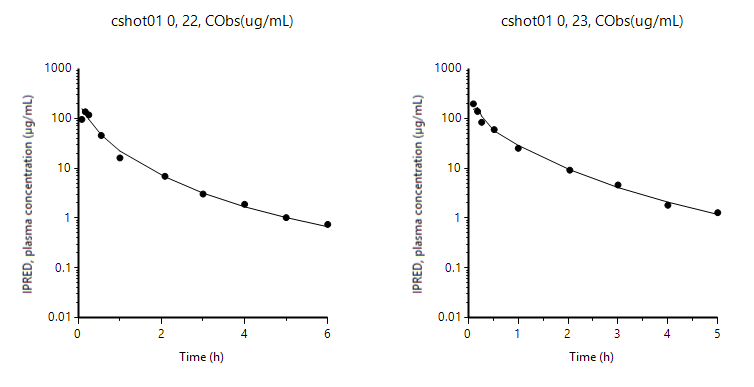
**

**USA2 horses**

**
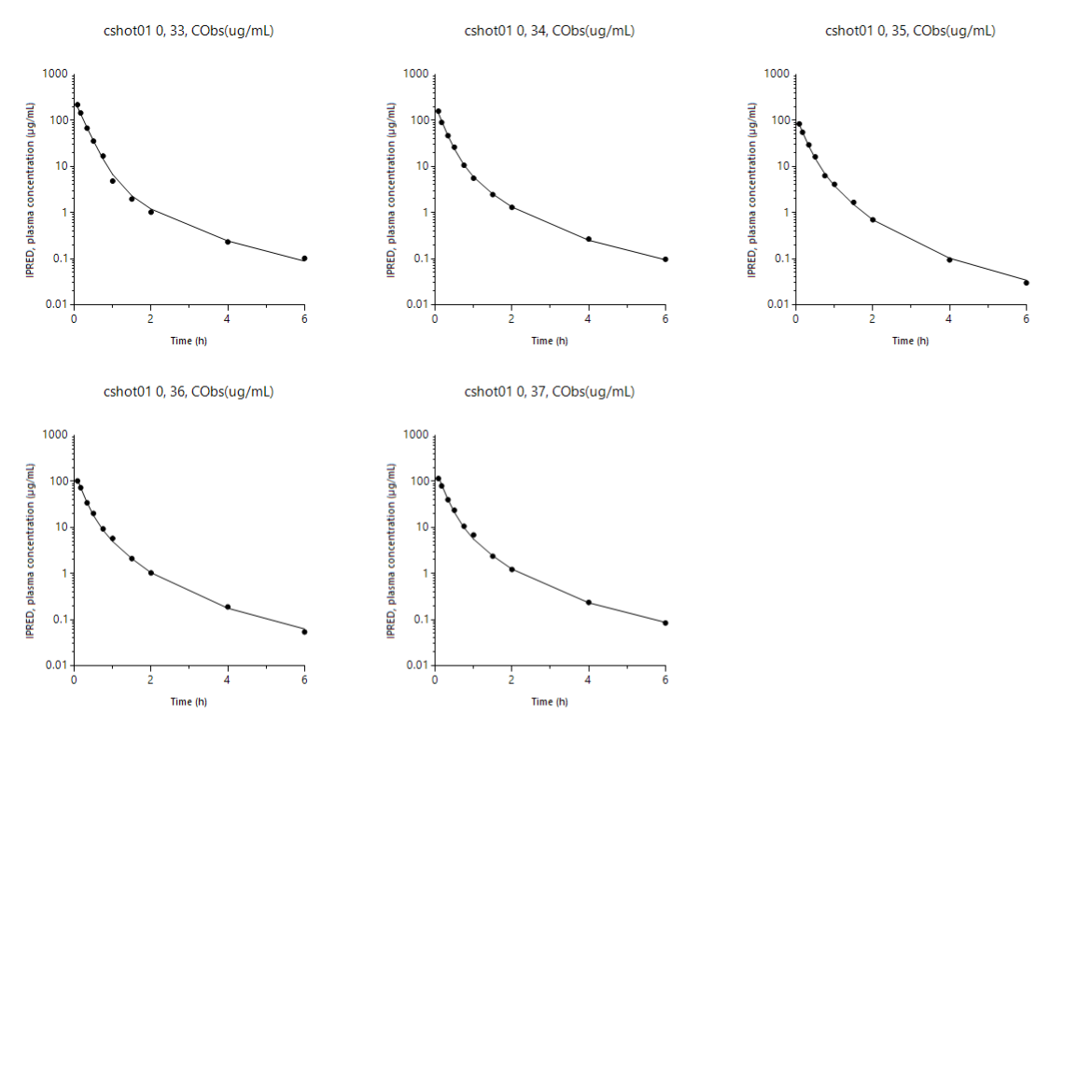
**

**
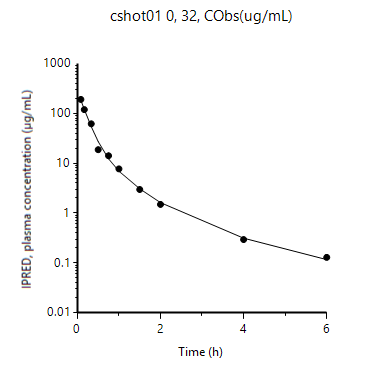
**

# Supplementary Figure S5: Goodness-of-fit (GOF) plots for the different routes of administration (IV, IM)

Figures S5A to S5J are Goodness-of-fit (GOF) plots for the different routes of administration (IV, IM) and the different formulations of BP (BP-PROC, BP Na, benzathine BP in combination with BP-PROC, BP from Penethamate). These figures support the previous adjustments stratified by formulation/route of administration and they indicate that it was reasonable to postulate that the disposition of BP as a substance can be described by a set of typical values of common parameters, and this, whatever its formulation and route of administration in France, USA1, USA2, Japan and Sweden.

**Figure S5A: DV vs PRED.** Logarithmic Plot of the dependent variable (DV) i.e. of plasma BP concentrations (µg/mL) after the IV or IM administration of different BP formulations versus population predicted plasma BP concentrations (PRED) (no random component). The plot shows observed vs. fitted values of the model function. Ideally, they should fall close to the line of unity y=x.

*For the logarithmic scale, data are evenly distributed about the line of identity, indicating no major bias in the population component of the model*.

**Figure S5B: DV vs PRED.** Arithmetic plot of the dependent variable (DV) i.e. of plasma BP concentrations (µg/mL) after the IV or IM administration of different BP formulations versus population predicted plasma BP concentrations (PRED) (no random component). The plot shows observed vs. fitted values of the model function. Ideally, they should fall close to the line of unity y=x.

*For the arithmetic scale, data are evenly distributed about the line of identity, indicating no major bias in the population component of the model*.


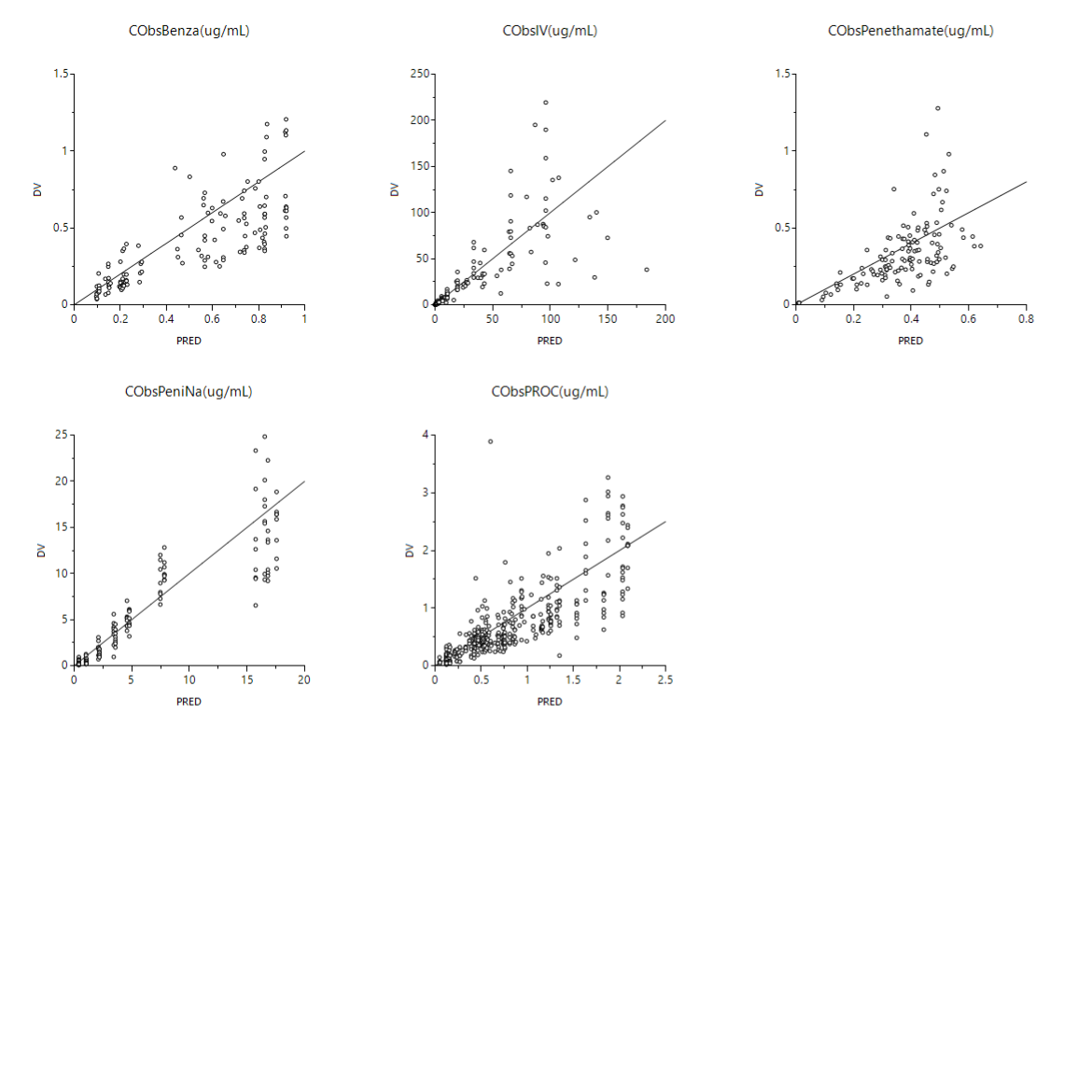


**Figure S5C: DV vs IPRED.** Logarithmic plot of the dependent variable (DV) i.e. of observed plasma BP concentrations (µg/mL) after the IV or IM administration of different formulations of BP versus individual predicted plasma BP values (IPRED). Individual predictions are obtained by setting random effects to the 'post hoc' or empirical Bayesian estimate of the random effects for the individual from which the DV observation was made. Thus, the plot shows observed vs fitted values of the model function. Ideally, they should fall close to the line of unity y=x.

*For the logarithmic scale, data are evenly distributed about the line of identity, indicating no major bias in the random component of the model allowing an appropriate individual fitting.*


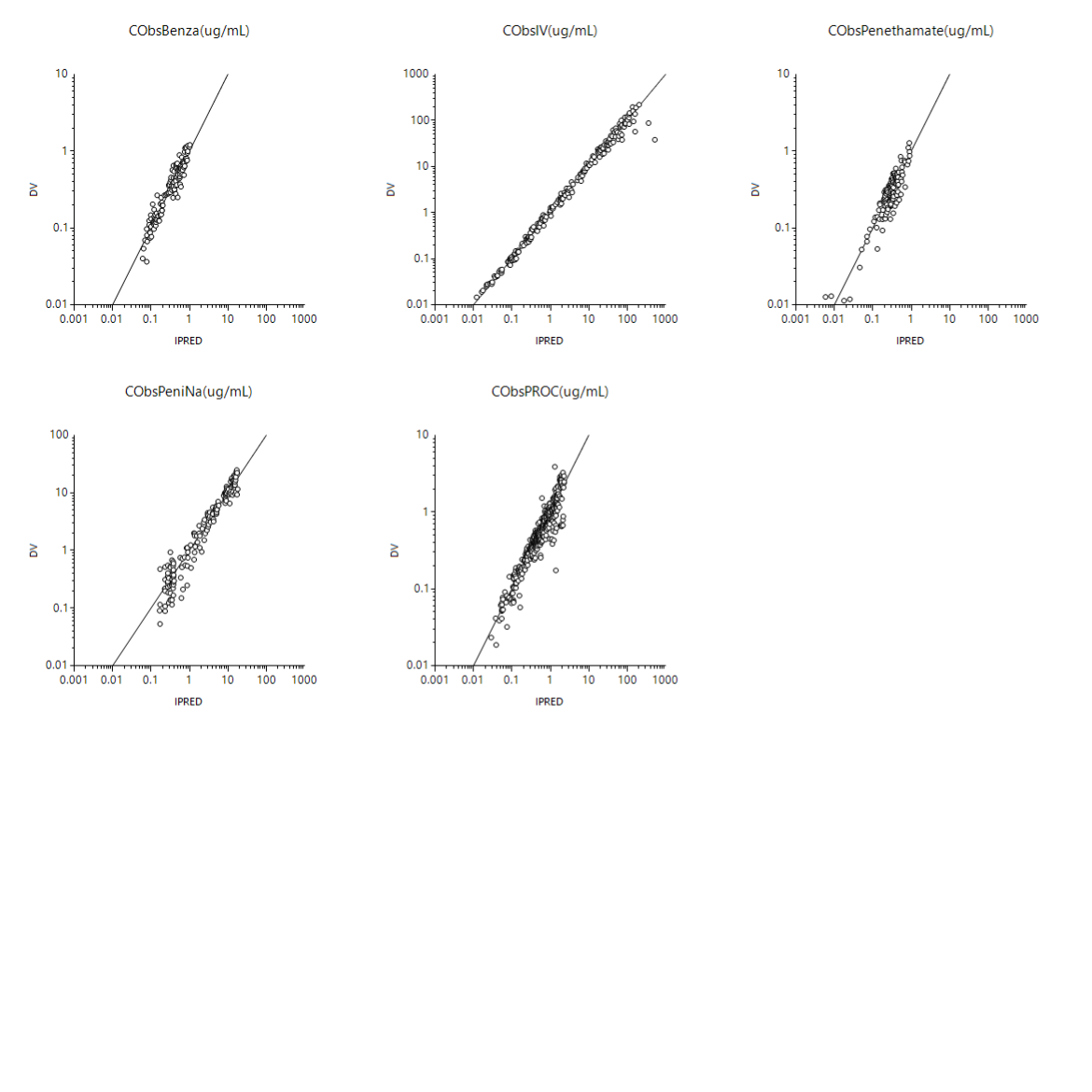


**Figure S5D DV vs IPRED:** Arithmetic plot of the dependent variable (DV) i.e. of observed plasma BP concentrations (µg/mL) after the IV or IM administration of different formulations of BP versus individual predicted plasma BP values (IPRED). Individual predictions are obtained by setting random effects to the 'post hoc' or empirical Bayesian estimate of the random effects for the individual from which the DV observation was made. Thus, the plot shows observed vs fitted values of the model function. Ideally, they should fall close to the line of unity y=x.

*For the arithmetic scale, data are evenly distributed about the line of identity, indicating no major bias in the random component of the model allowing an appropriate individual fitting.*


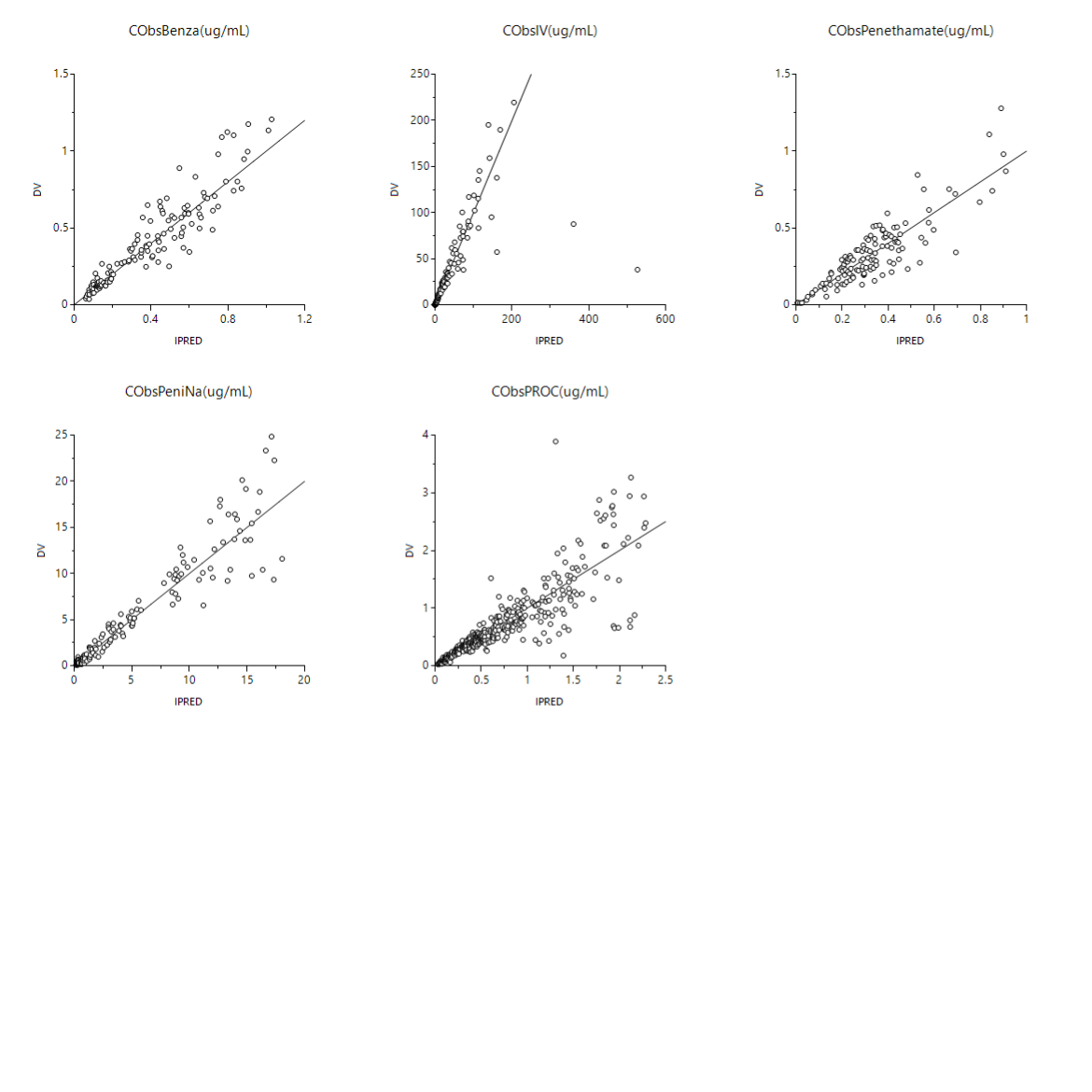


**Figure S5E:** Histogram plot of the conditional weighted residual values (CWRES). Values of CWRES should be approximately N(0,1) and hence concentrated between y=-2 and y=+2. Values significantly above 3 or below -3 are suspect and may indicate a lack of fit and/or model misspecification.


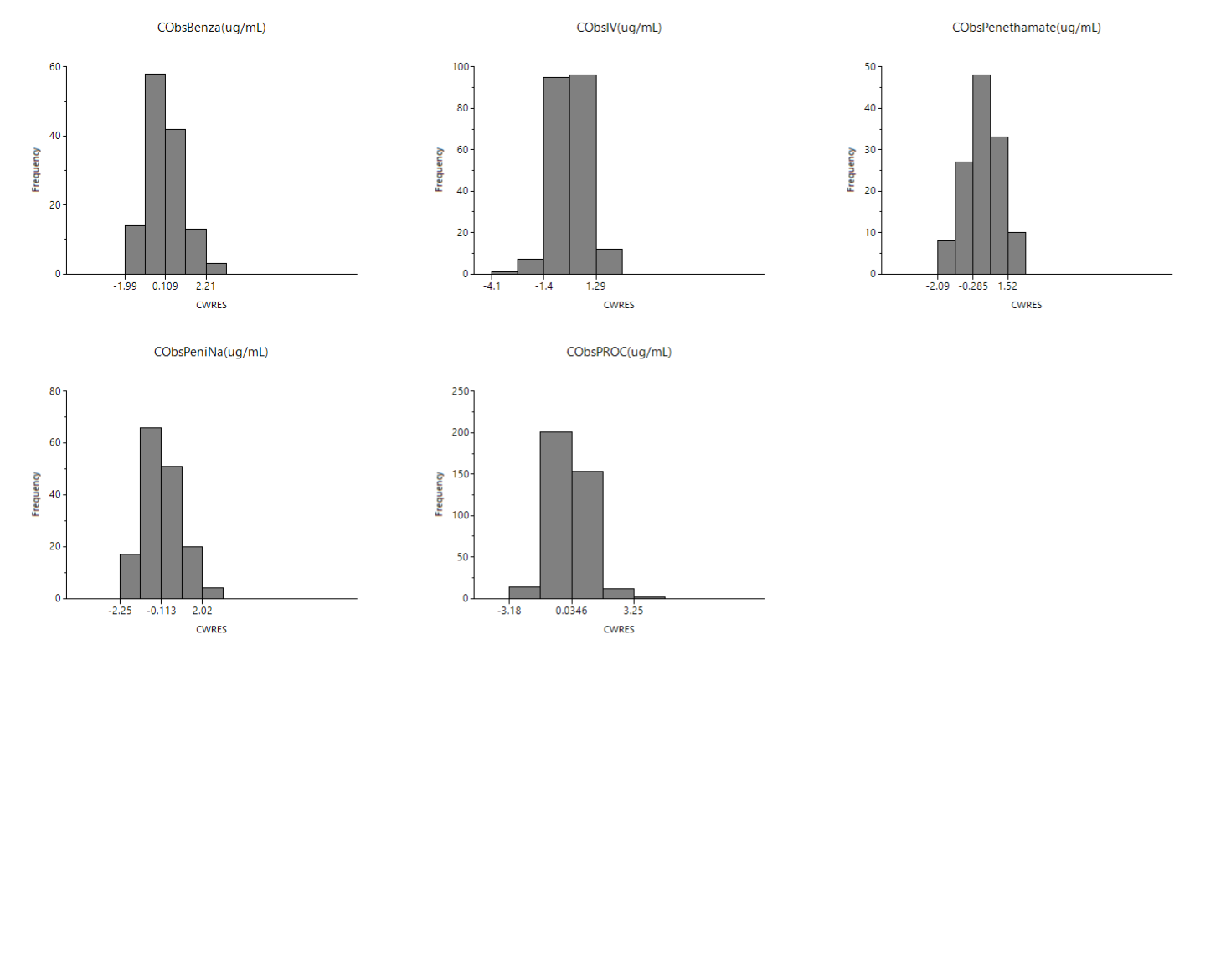


**Figure S5F: CWRES vs Time after administration**. Plot of CWRES (conditional weighted residuals), a proposed replacement for the classical WRES (weighted residuals) goodness of fit statistic, against IVAR (time). Values of CWRES should be approximately N(0,1) and hence concentrated between y=-2 and y=+2. Values significantly above 3 or below -3 are suspect and may indicate a lack of fit and/or model misspecification.

*Inspection of the figure shows that data are evenly distributed about zero (see the trends as given by the blue line), indicating no major bias in the structural model. The blue curve takes into account the sign of the residuals (positive or negative) while the red curve and its reflection only consider absolute value of residuals. Ideally, the blue line should be at 0 and the red line (with its negative reflection) should not show any fanning. Fanning indicates room for improving the distribution of residuals. For BP benzathine, the blue line is over the line 0 indicating some bias.*


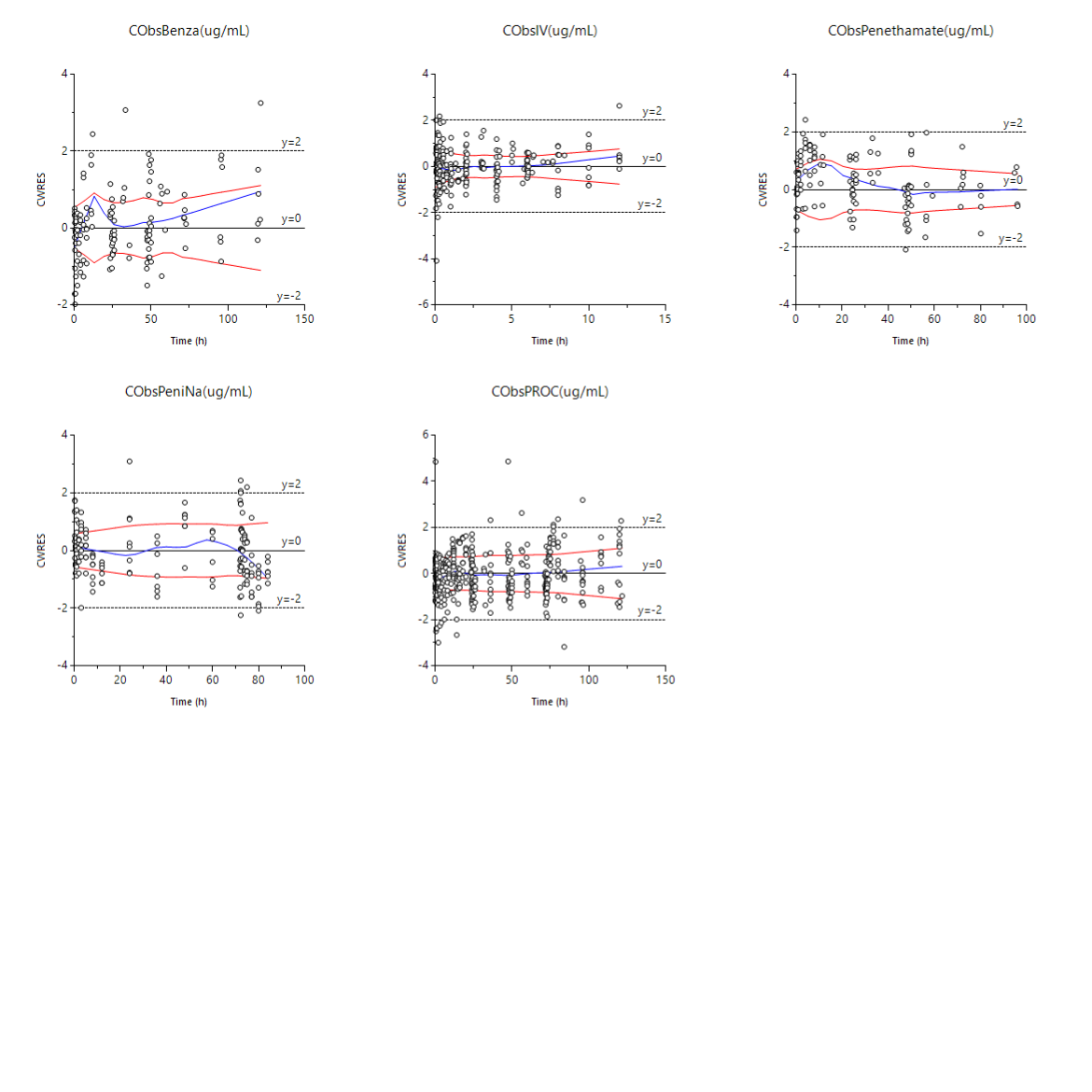


**Figure S5G: CWRES vs PRED.** Plot of CWRES (conditional weighted residuals), a proposed replacement for the classical WRES (weighted residuals) goodness of fit statistic, against PRED i.e. the population predictions (the predictions obtained by setting the random effect values to zero) used for the X axis. Values of CWRES should be approximately N(0,1) and hence concentrated between y=-2 and y=+2. Values significantly above 3 or below -3 are suspect and may indicate a lack of fit and/or model misspecification.

*Inspection of the figure shows that data are evenly distributed about zero (see the trends as given by the blue line), indicating no bias. Ideally, the blue line should be at 0 and the red line (with its negative reflection) should not show any fanning. Fanning indicates room for improving the distribution of residuals.*


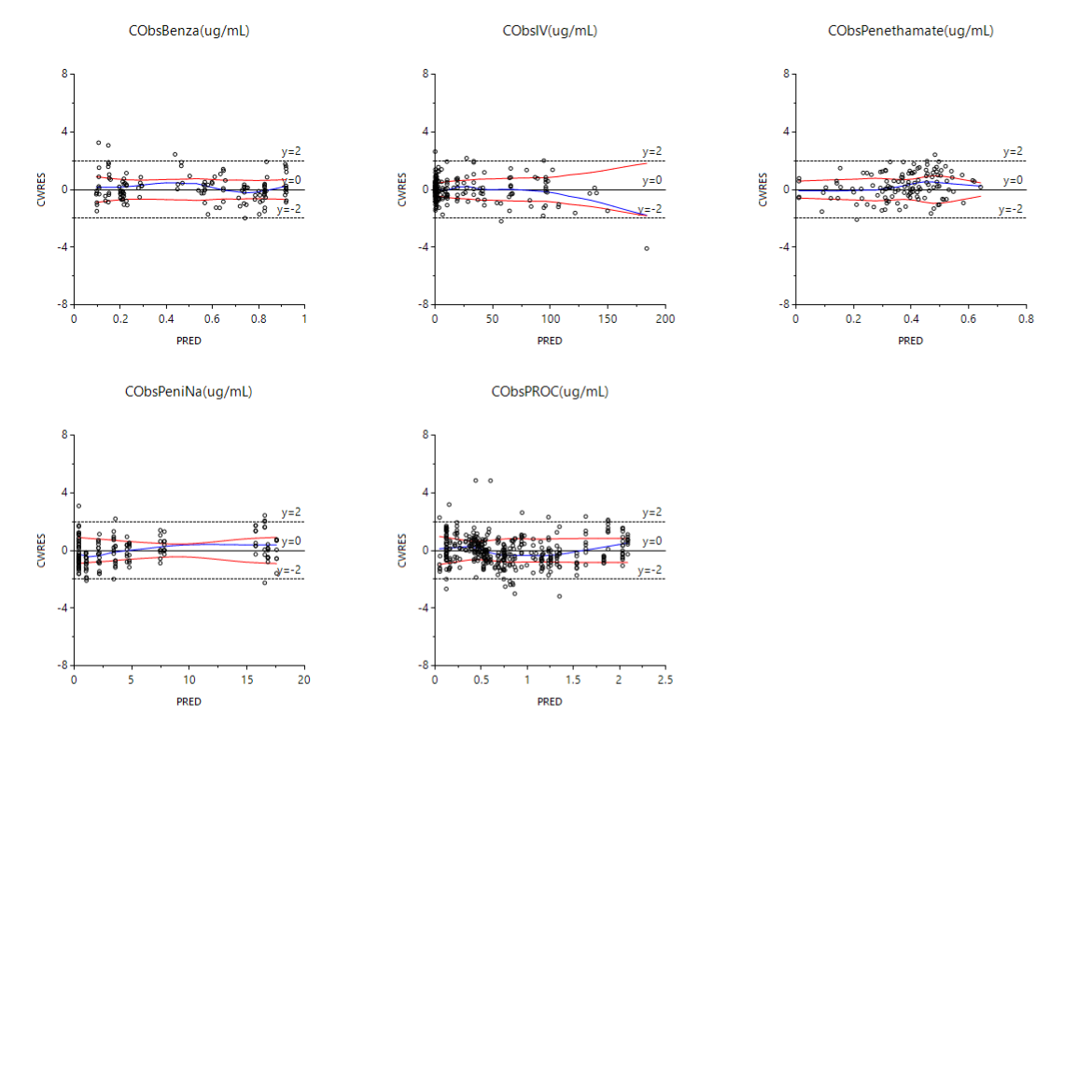


**Figure S5H: Eta Histogram.** Histogram plot of the ETA values. ETA is the Random effect describing the deviation of the individual Empirical Bayes Estimate of the parameter from the typical population parameter estimate. They were estimated using an exponential model with a mean of 0 for the different parameters.

*The presence of a large modal value indicates the presence of an ETA shrinkage and the impossibility of correctly estimating the EBE.*


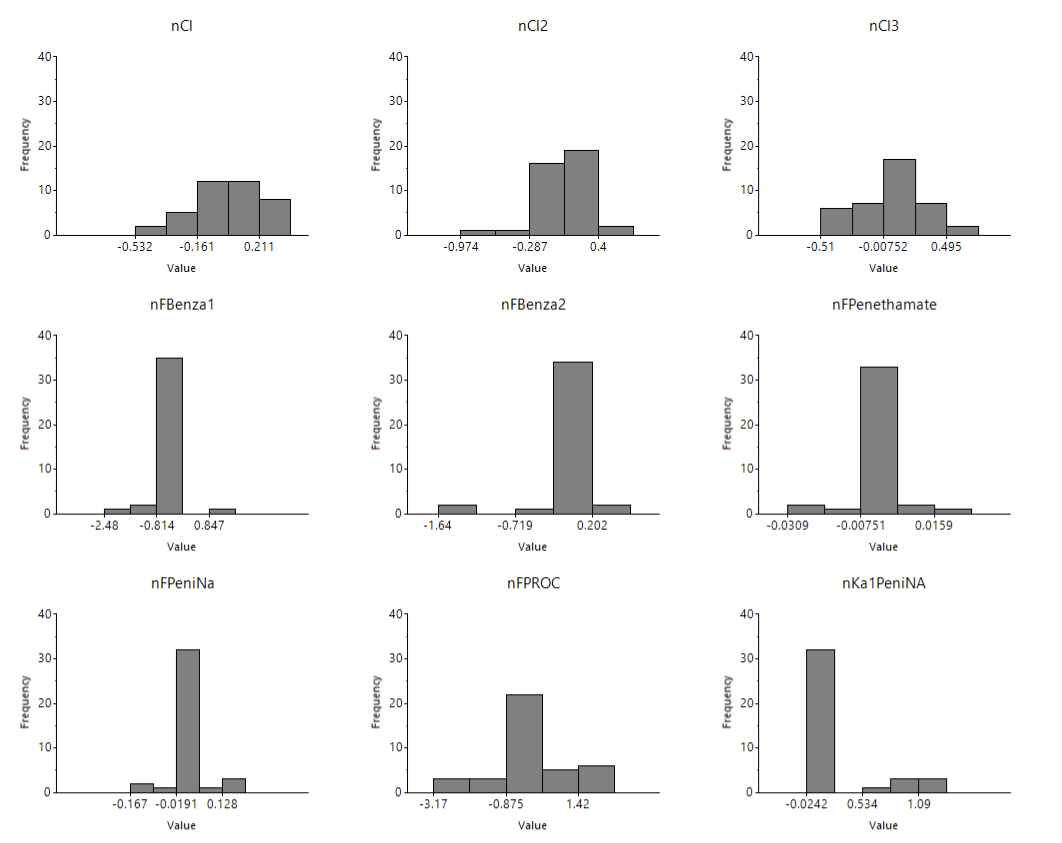


**Figure S5I: CWRES QQ plot.** Quantile-quantile (QQ) plot for each eta in the model. If the components of eta are well described by a normal distribution, plotted values will fall roughly along a straight line of unity y=x. Significant deviations from normality, particularly in the tails of the distribution, can be seen by deviations from this line.


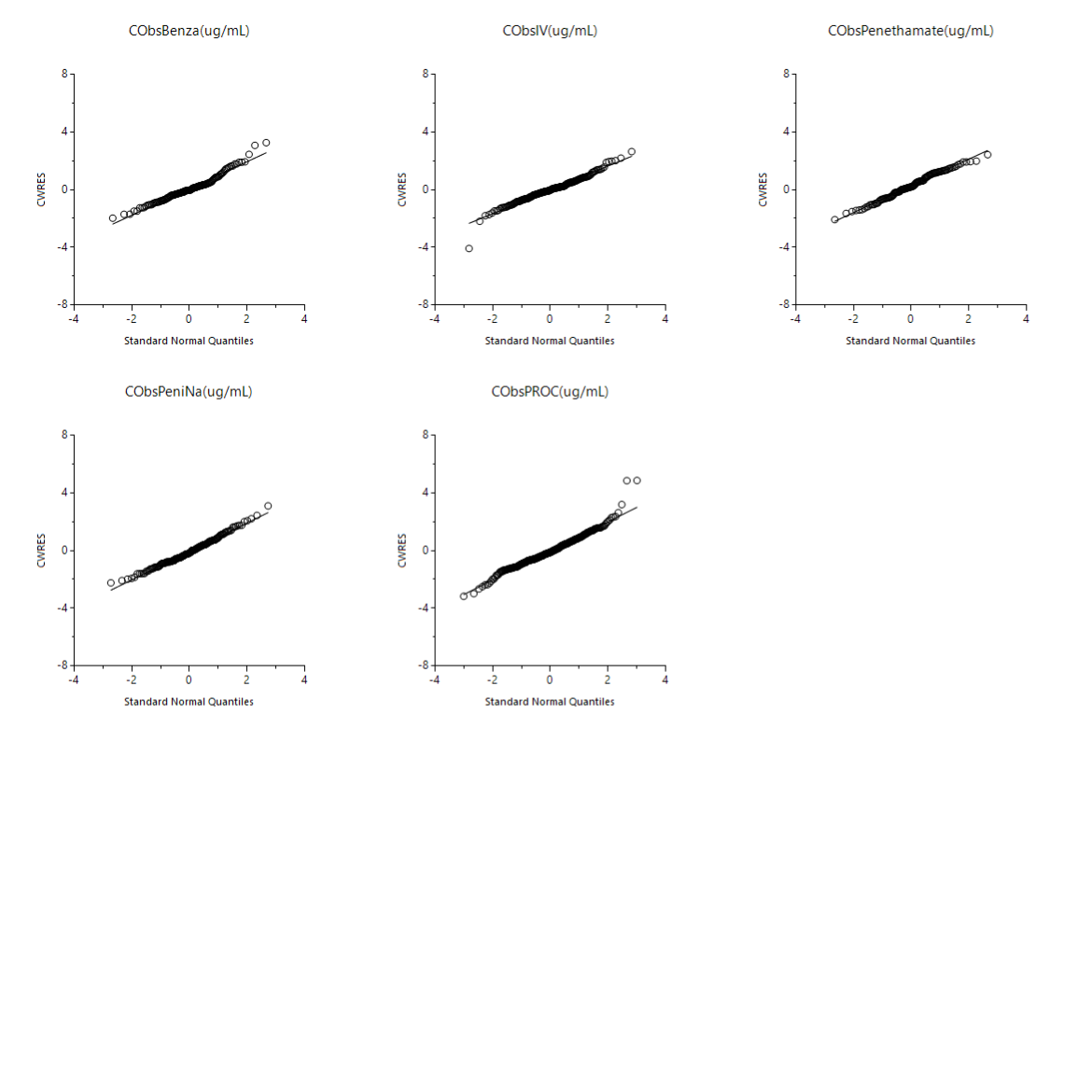


**Figure S5J:** Semi logarithmic plot (latticed by individual) of dependent variable (plasma BP concentration, black circles) and individual predicted curve (black line) vs Time (h) after dose administration (one row per horses, 31 horses).

**French horses (BP-BENZA, IV, penethamate & BP-PROC)**


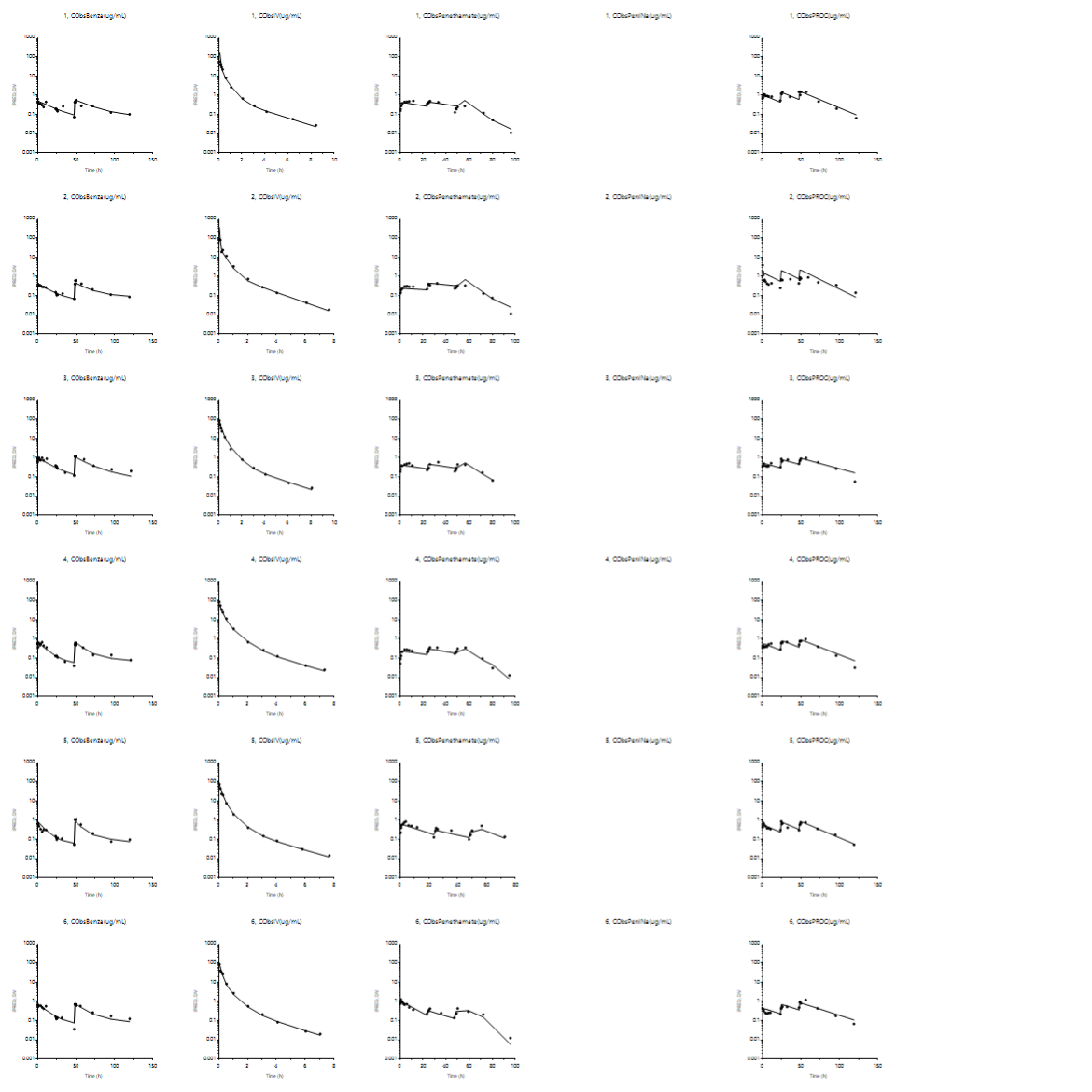


**Japanese horses (BP-PROC)**


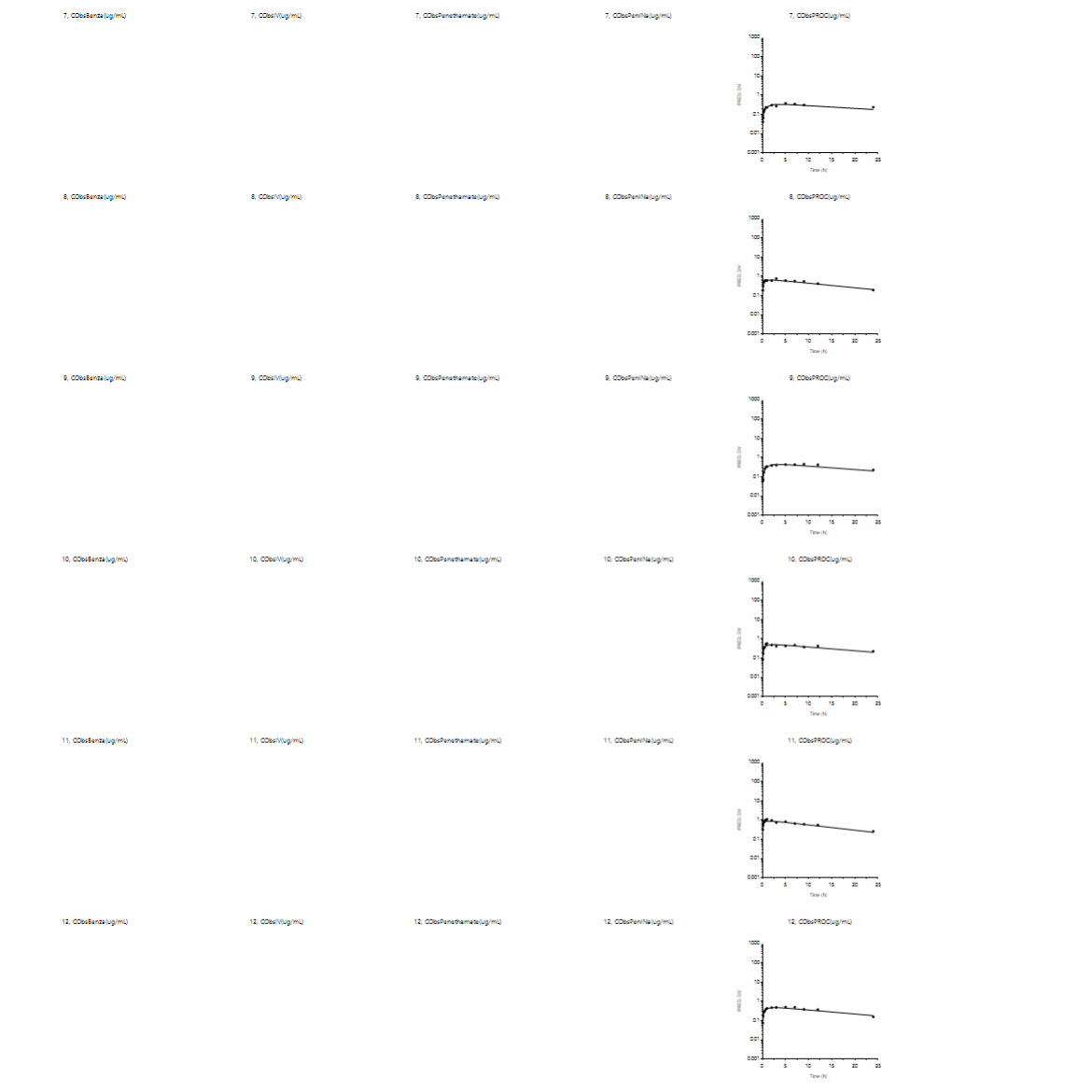


**USA1 horses (IV and BP-PROC)**


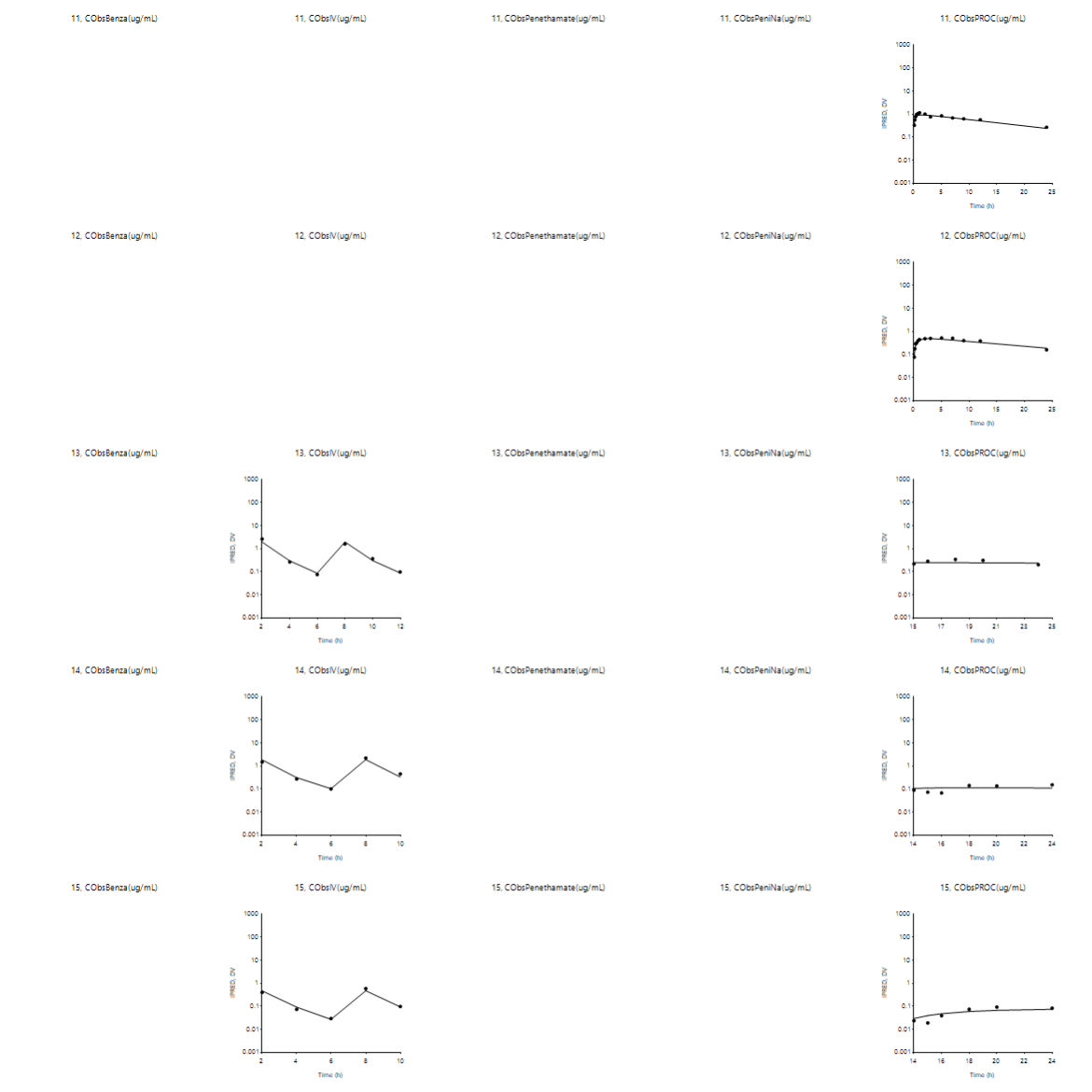


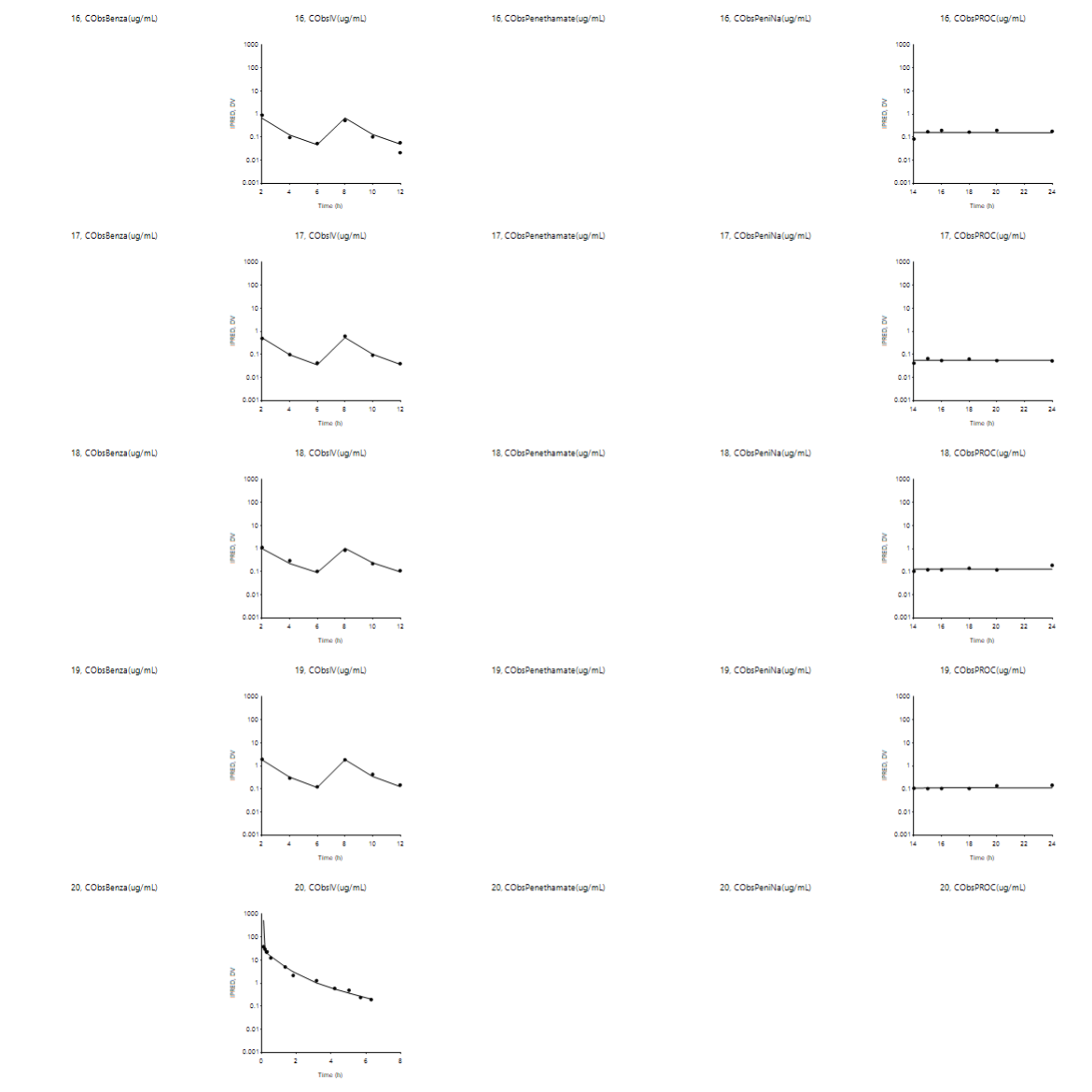


**Swedish horses (IV)**


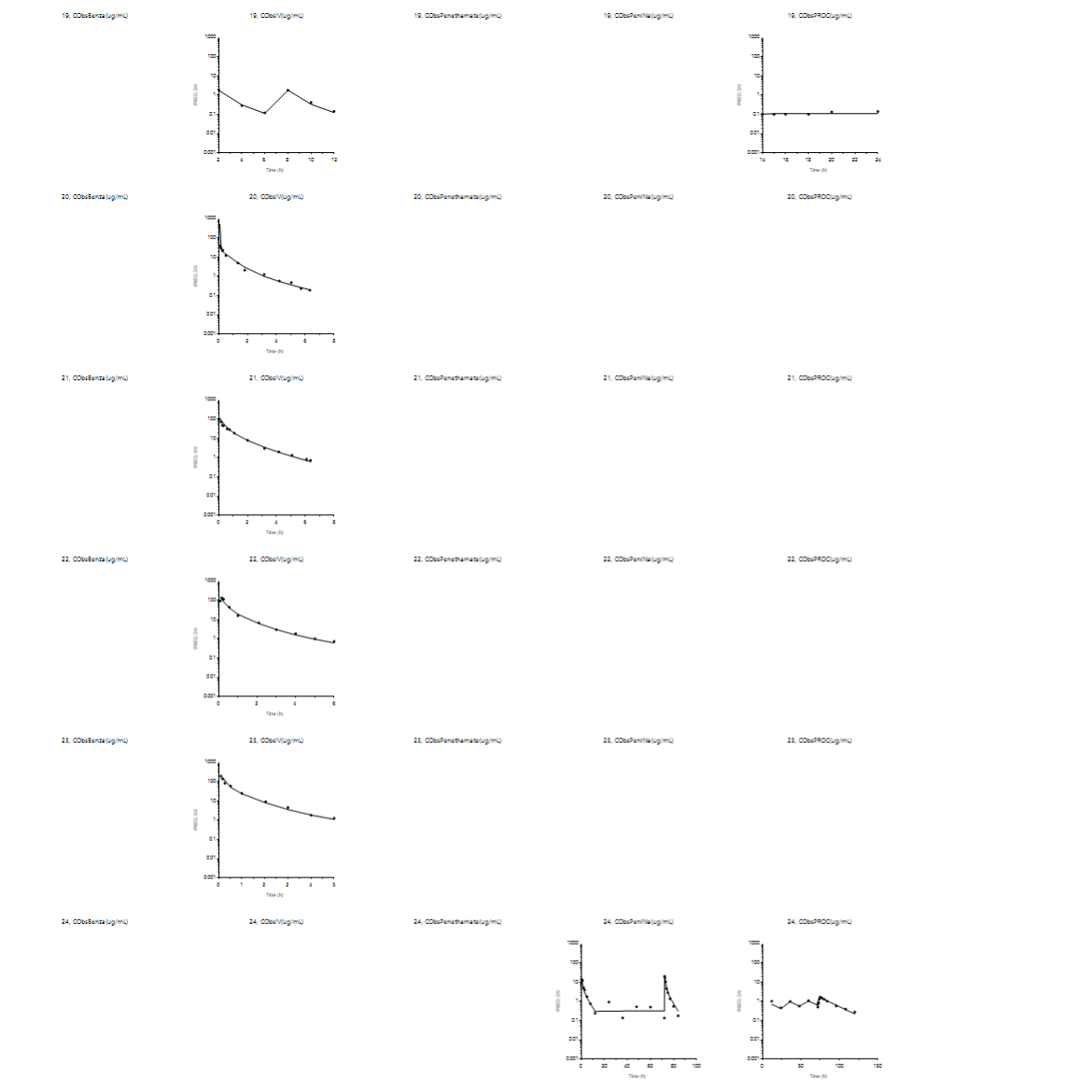


**Swedish horses (BP-PROC & BP-Na)**


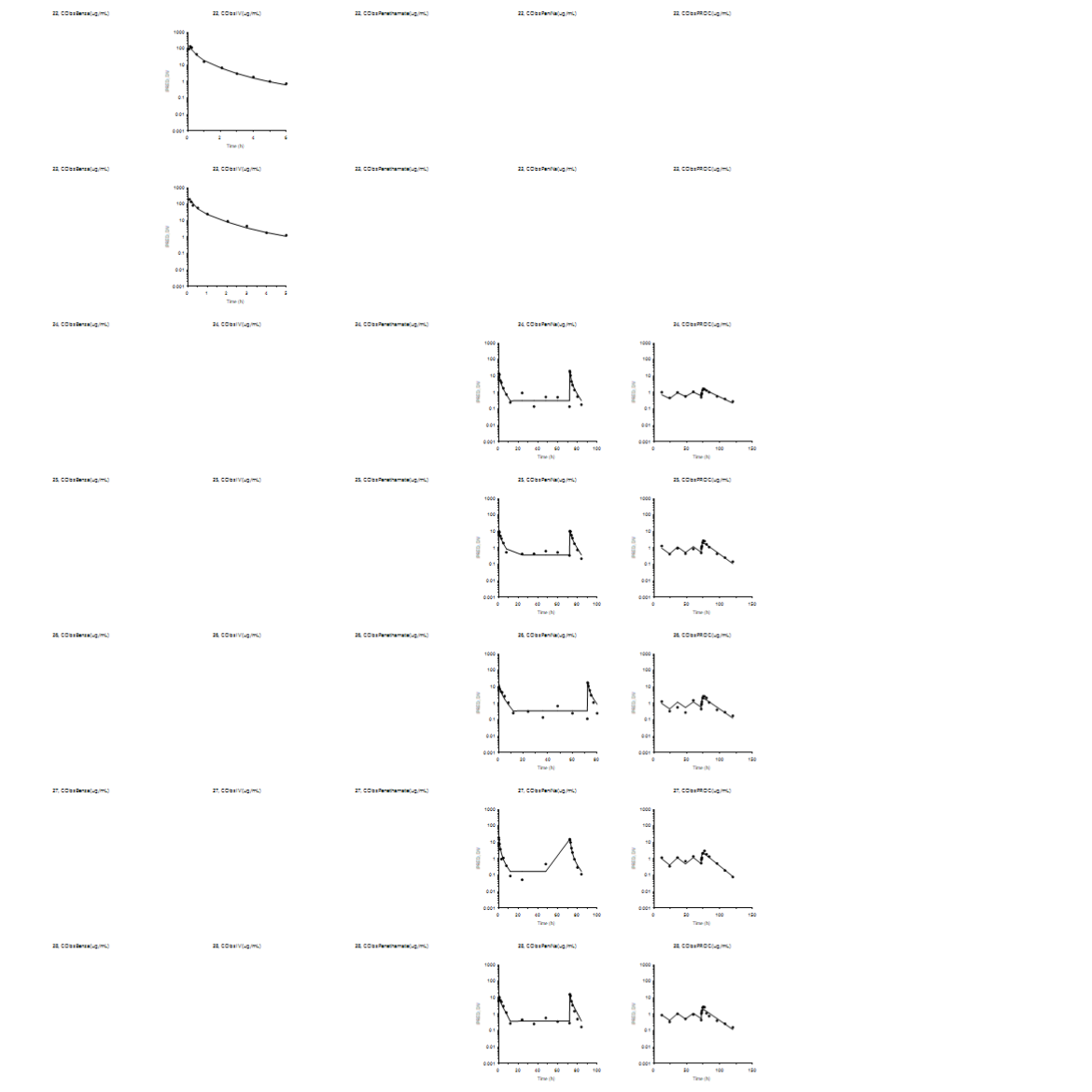


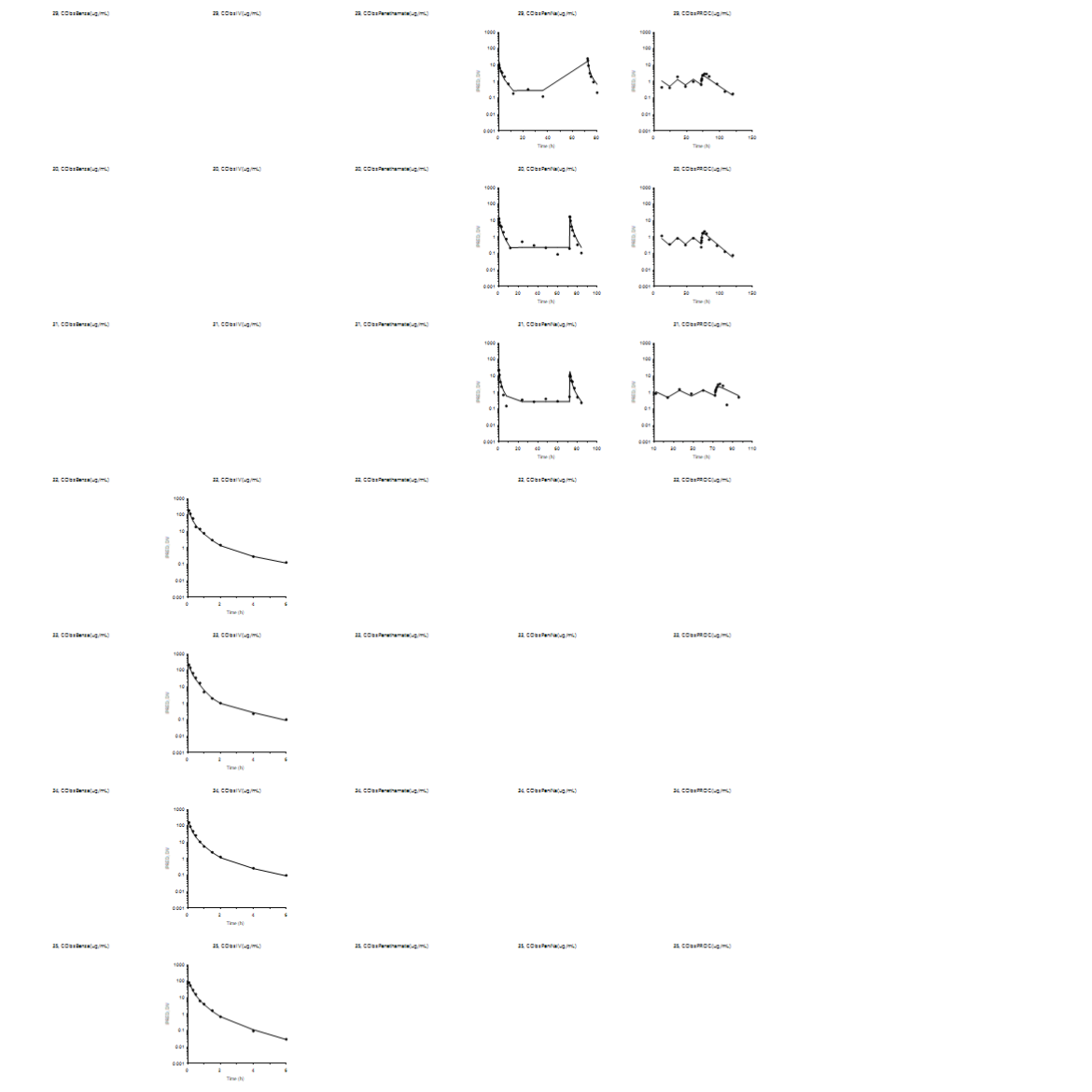


**USA2 horses (IV)**


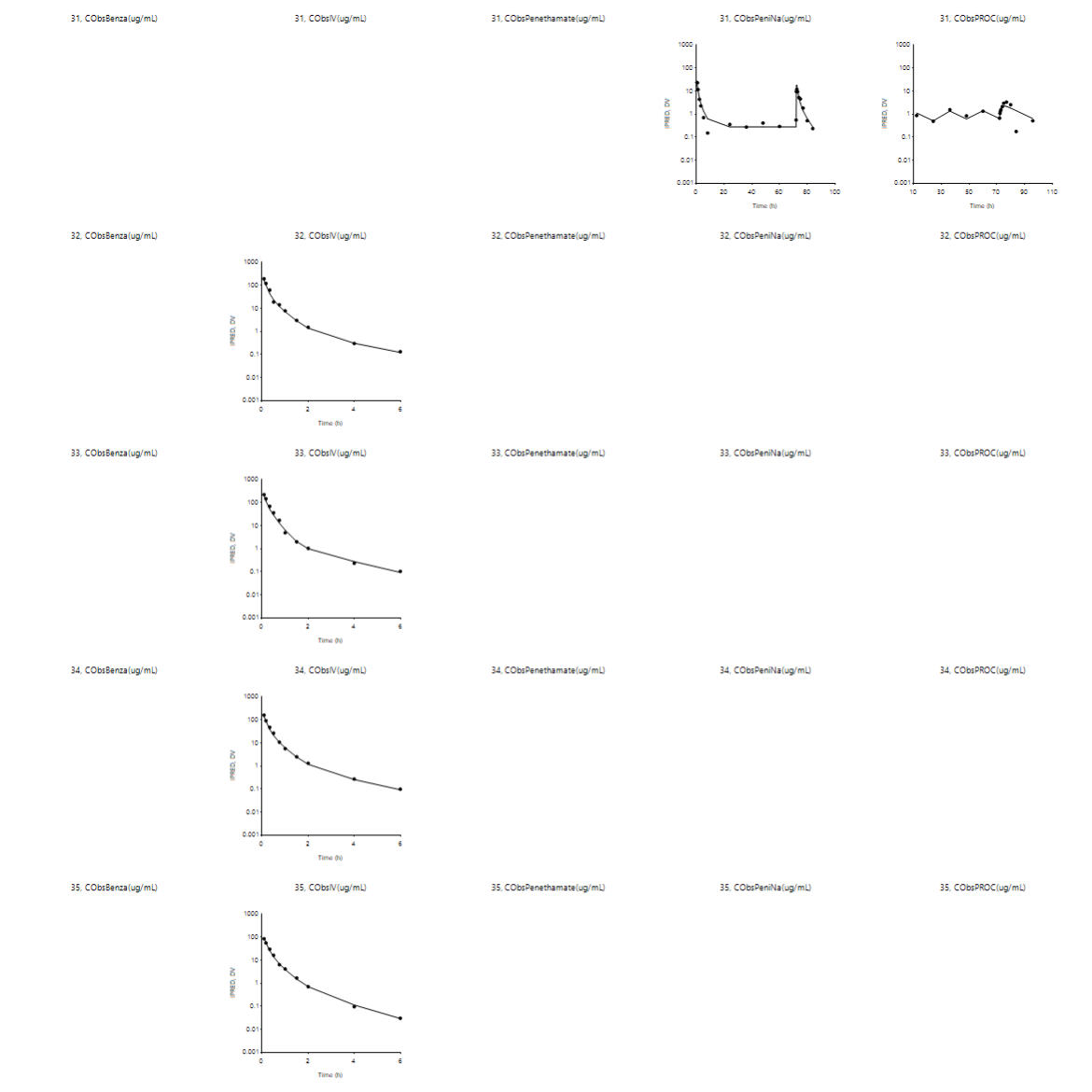


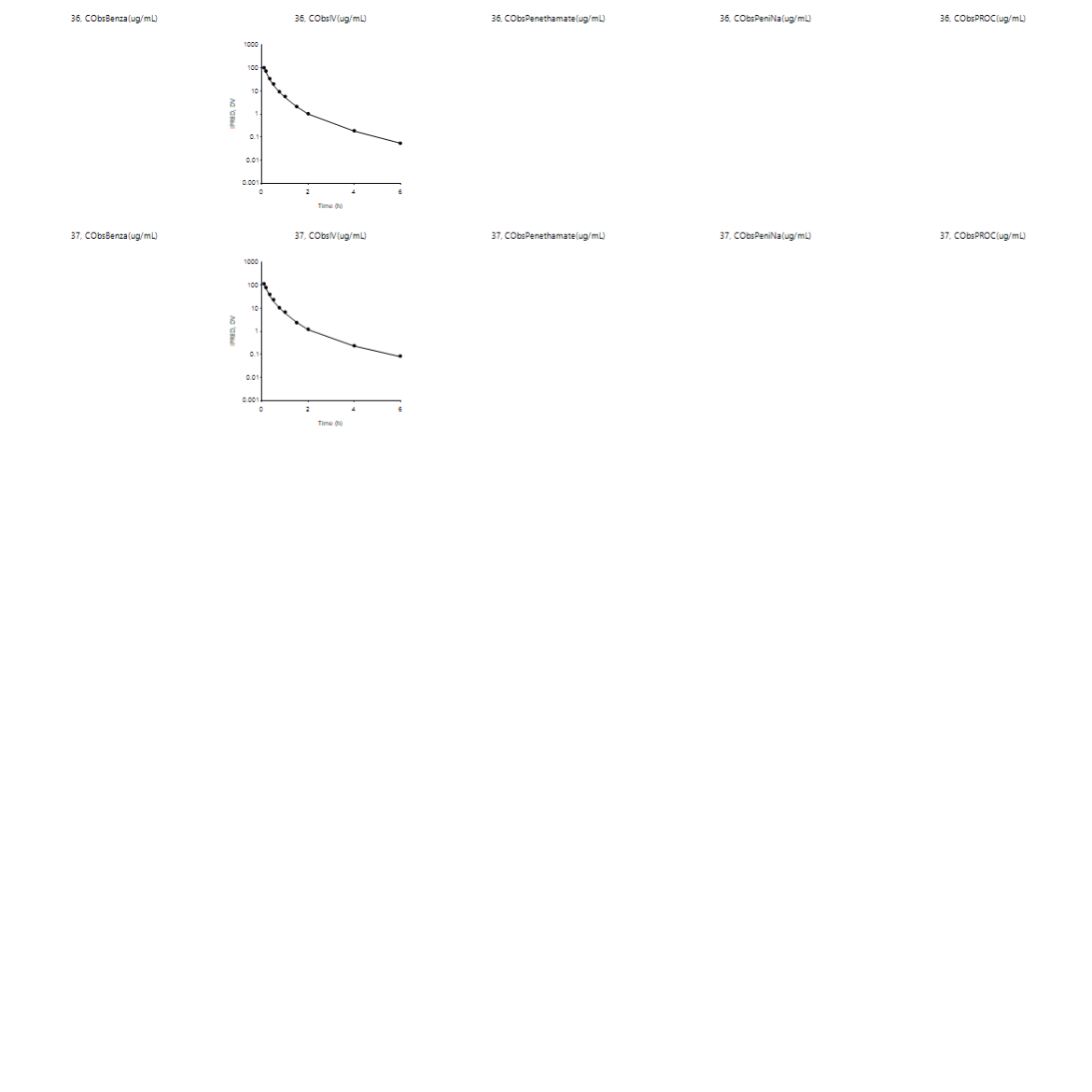


# Supplementary Table S1: Benzylpenicillin (BP) equivalence for the different salts and prodrug of BP used in the present meta-analysis. The table gives Molecular Weight (MW) and the different conversion factors that was used to express dosing in terms of BP when dosing was reported either for the BP salt or using International Units (IU).

| **Substances** | **MW** | **IU per mg of substance** | **Ratio potency BP=1780** |
| --- | --- | --- | --- |
| BP (BP) | 334.4 | 1780 | 1.00 |
| Potassium BP (BP-K) | 372.5 | 1598 | 0.90 |
| Sodium BP (BP-Na) | 356.4 | 1670 | 0.94 |
| Penethamate hydriodide | 561.5 | 1060 | 0.60 |
| Penethamate | 433.6 | 1373 | 0.77 |
| Procaine BP (BP-PROC) | 570.7 | 1043 | 0.59 |
| Procaine BP monohydrate | 588.7 | 1011 | 0.57 |
| Benzathine BP (BP-BENZA) | 909.1 | 1333 | 0.75 |

Supplementary Table S2: Dosage used for the different pharmaceutical products considered in the meta-analysis:

| **Countries** | **Trade name**  **Pharmaceutical form**  **Route of administration**  **(company)** | **Active ingredients** | **Number of horses** | **Dose SPC (mg/kg or IU/kg)** | **Dose as BP (mg/kg)** |
| --- | --- | --- | --- | --- | --- |
| France | Depocilline®  Suspension  IM  (Intervet/MSD) | BP-PROC | 6 | 7-17 mg/kg as BP | 10 mg/kg,  3 doses at 24h interval |
| France | Duplocilline®  Suspension  IM  (Intervet/MSD) | BP-BENZA + BP-PROC | 6 | 12.4 mg/kg as BP | 12.4 mg/kg  Two administrations at 48h interval. For data analysis, dose was split into 5.96376 mg/kg as BP-PROC and 6.43624 mg/kg as BP-BENZA |
| France | Penetavet®  Suspension  IM  (Boehringer Ingelheim) | Penethamate (as hydriodide) | 6 | 7.72 g in toto as penethamate at day1 followed by 3.86 g in toto at day2 and day3. | For a 500 kg BW horse: 11.91 mg/kg at Day 1 followed by 5.96 mg/kg at day 2 and day 3 |
| France | Benzylpenicillin G Panpharma 5MUI®  IV  Solution  (Panpharma) | BP-Na | 6 | 22,000 IU/kg | 12.36 mg/kg  Single dose |
| Japan | Procaine BP G sol for Animals "KS"®,  Suspension  IM  (Kyoritsu Seiyaku Inc.) | BP-PROC | 6 | 10,000 IU/kg as BP-PROC | 5.617 mg/kg  Single dose |
| Sweden | Geepenil ®  IV  Solution  (Orion Pharma Animal Health) | BP-Na | 2 | 24.84 & 26.77 mg/kg as BP-Na | 23.30 & 25.11 mg/kg  Single dose |
| Sweden | Bensylpenicillin Meda®  IV  Solution  (Meda) | BP-Na | 2 | 17.53 and 19.86 mg/kg as BP-Na | 16.44 & 18.63 mg/kg  Single dose |
| Sweden | Geepenil ®  IM  Solution  (Orion Pharma Animal Health) | BP-Na | 8 | About 15 mg/kg as BP-Na | about 14.07 mg/kg  (7 administrations  at 12 h interval) |
| Sweden | Penovet Vet®  Suspension  IM  (Boehringer Ingelheim) | BP-PROC | 8 | About 21,000 IU/kg | About 11.80 mg/kg  Four doses at 24h interval |
| USA1 & USA2 | Pfizerpen  Solution  IV  (Pfizer) | BP-K | 7+6 | 22,000 IU/kg | USA1:12.36 mg/kg  Two administrations  at 6h interval  USA2: single dose administration |
| USA1 | Norocillin®  Suspension  IM  (Norbrook Laboratories Limited) | BP-PROC | 7 | 22,000 IU/kg | 12.36 mg/kg  Single dose 6 h  after the second  IV administration |

# Supplementary table S3: Demographics of the 40 horses; X: data unknown

| **Metastudy Horse ID** | **Country** | **Sex** | **Weight** | **Age(years)** | **Breed** |
| --- | --- | --- | --- | --- | --- |
| 1 | France | Gelding | 521 | 11 | Half blood arabian |
| 2 | France | Female | 450 | 5 | French trotter |
| 3 | France | Gelding | 456 | 11 | Purebred spanish |
| 4 | France | Female | 539 | 6 | French trotter |
| 5 | France | Gelding | 538 | 11 | Saddlebred |
| 6 | France | Female | 511 | 6 | French trotter |
| 7 | Japan | Female | 425 | 7 | Thouroughbred |
| 8 | Japan | Female | 500 | 7 | Thouroughbred |
| 9 | Japan | Male | 530 | 4 | Thouroughbred |
| 10 | Japan | Male | 530 | 7 | Thouroughbred |
| 11 | Japan | Male | 441 | 6 | Thouroughbred |
| 12 | Japan | Male | 481 | 7 | Thouroughbred |
| 13 | USA1 | Female | 545 | 19 | Paint |
| 14 | USA1 | Female | 497 | 19 | Quarterhorse |
| 15 | USA1 | Gelding | 538 | 10 | Quarterhorse cross |
| 16 | USA1 | Gelding | 562 | 9 | Quarterhorse |
| 17 | USA1 | Gelding | 498 | 7 | Quarterhorse |
| 18 | USA1 | Gelding | 560 | 11 | Quarterhorse |
| 19 | USA1 | Gelding | 525 | 19 | Thoroughbred |
| 20 | Sweden | Gelding | 473 | X | Standardbred trotters |
| 21 | Sweden | Female | 484 | 24 | Standardbred trotters |
| 22 | Sweden | X | 438 | X | X |
| 23 | Sweden | Gelding | 308 | 16 | Mixed Pony |
| BP PROC study with Penovet | | | |  |  |
| 24 | Sweden | Female | 557 | 16 | Standardbred trotters |
| 25 | Sweden | Female | 528 | 18 | Standardbred trotters |
| 26 | Sweden | Female | 494 | 23 | Standardbred trotters |
| 27 | Sweden | Female | 474 | 20 | Standardbred trotters |
| 28 | Sweden | Female | 578 | 13 | Standardbred trotters |
| 29 | Sweden | Female | 592 | 15 | Standardbred trotters |
| 30 | Sweden | Gelding | 504 | 15 | Standardbred trotters |
| 31 | Sweden | Female | 576 | 15 | Standardbred trotters |
| BP-Na study with Geepenil | | | |  |  |
| 28 | Sweden | Female | 581 | 7 | Standardbred trotters |
| 32 | Sweden | Female | 554 | 17 | Standardbred trotters |
| 26 | Sweden | Female | 518 | 17 | Standardbred trotters |
| 33 | Sweden | Female | 513 | 14 | Standardbred trotters |
| 29 | Sweden | Female | 577 | 9 | Standardbred trotters |
| 30 | Sweden | Gelding | 497 | 9 | Standardbred trotters |
| 31 | Sweden | Female | 549 | 9 | Standardbred trotters |
| 34 | Sweden | Female | 471 | 17 | Standardbred trotters |
| 35 | USA2 | Gelding | 472,7 | 7 | Thoroughbred |
| 36 | USA2 | Mare | 492,7 | 6 | Thoroughbred |
| 37 | USA2 | Gelding | 488,6 | 6 | Thoroughbred |
| 38 | USA2 | Mare | 579,5 | 6 | Thoroughbred |
| 39 | USA2 | Gelding | 529 | 6 | Thoroughbred |
| 40 | USA2 | Gelding | 509,5 | 6 | Thoroughbred |

# Supplementary Table S4: IV and IM dosage regimen scenario for which the PK/PD cutoffs have been computed using Monte Carlo simulations.

| Route | BP form | modality of administration | Dose or rate  (IU  per dosing) | dose per 24h  as BP (µg/kg) | interval of administration (h) | Duration  h |
| --- | --- | --- | --- | --- | --- | --- |
| IV | BP-Na or potassium salt | bolus | 22,000 | 12,360 | Single dose | 24 |
| IV | BP-Na or potassium salt | bolus | 33,000 | 18,450 | Single dose | 24 |
| IV | BP-Na or potassium salt | bolus | 44,000 | 24,720 | Single dose | 24 |
| IV | BP-Na or potassium salt | bolus | 22,000 | 12,360x2 | 12 | 24 |
| IV | BP-Na or potassium salt | bolus | 22,000 | 12,360x3 | 8 | 24 |
| IV | BP-Na or potassium salt | bolus | 22,000 | 12,360x4 | 6 | 24 |
| IV | BP-Na or potassium salt | Continuous infusion over 24 h | 22,000 | 12,360 |  | 24 |
| IV | BP-Na or potassium salt | Continuous infusion over 24 h | 33,000 | 18,450 |  | 24 |
| IV | BP-Na or potassium salt | Continuous infusion over 24 h | 44,000 | 24,720 |  | 24 |
| IM | Procaine BP- |  | 22,000 | 12,360x3 | 24 | 72 |
| IM | Procaine BP- |  | 44,000 | 24,720x3 | 24 | 72 |
| IM | Procaine BP |  | 22,000 | 12,360x6 | 12 | 72 |
| IM | Sodium or potassium BP |  | 22,000 | 12,360x4 | 6 | 72 |
| IM | Sodium or potassium BP |  | 22,000 | 12,360x3 | 8 | 72 |
| IM | Sodium or potassium BP |  | 22,000 | 12,360x2 | 12 | 72 |
| IM | Penethamate |  | 22,000  & 11,000 | 12,360+6180+6180 | 24 | 72 |
| IM | Procaine plus benzathine BP |  | 22,000 | (5963 as procaine BP  +6436 as benzathine BP | 48 | 96 |

**Supplementary Table S5:** Population secondary parameters of BP after an IV administration in horses as obtained with a 3-compartment model (estimates, CV%, 2.5 and 97.5% percentiles). Values are for the model without covariate i.e. for the French horses.

| Secondary | Estimate | Units | CV% | 2.5% CI | 97.5% CI |
| --- | --- | --- | --- | --- | --- |
| tvKe | 4.53 | 1/h | 5.98 | 3.99 | 5.06 |
| tvK12 | 1.19 | 1/h | 35.44 | 0.36 | 2.02 |
| tvK13 | 0.235 | 1/h | 18.82 | 0.148 | 0.323 |
| tvK21 | 2.72 | 1/h | 25.75 | 1.34 | 4.10 |
| tvK31 | 0.495 | 1/h | 10.05 | 0.397 | 0.594 |
| tvVss | 203 | mL/kg | 25.57 | 101 | 305 |
| tvVarea | 1030 | ml/kg | 15.7 | 710 | 1350 |
| tvMRTIV | 0.422 | h | 5.48 | 0.376 | 0.468 |
| tvAlpha | 6.77 | 1/h | 14.31 | 4.86 | 8.68 |
| tvBeta | 1.93 | 1/h | 15.03 | 1.36 | 2.51 |
| tvGamma | 0.467 | 1/h | 9.08 | 0.383 | 0.550 |
| tvHL_alpha | 0.102 | h | 14.31 | 0.074 | 0.131 |
| tvHL_Beta | 0.359 | h | 15.03 | 0.252 | 0.465 |
| tvHL_Gamma | 1.48 | h | 9.08 | 1.22 | 1.75 |
| tvA | 94.17 | kg/mL | 30.62 | 37.27 | 151 |
| tvB | 18.05 | kg/mL | 29.92 | 7.40 | 28.71 |
| tvC | 0.788 | kg/mL | 26.05 | 0.383 | 1.19 |

*Ke: rate constant of elimination from the central compartment; K12 and K21: first-order rate constants of exchange between compartment 1 and 2. K13 and K31: first-order rate constants of exchange between compartment 1 and 3. Vss: steady-state volume of distribution; MRTIV: Mean residence times; Alpha: slope of the initial phase; Beta: slope of the second distribution phase; Gamma: slope of the terminal phase; Half_life; Gamma Half-life of the terminal phase. Half_life_Alpha: half-life of initial distribution phase; Half_life_Beta: half-life of second distribution phase. HL Gamma: Half-life of the terminal phase. A, B and C are pre-exponential concentrations of the Alpha, Beta and Gamma phases respectively computed for a standard dose of 12 mg/kg of BP.*

# Supplementary Table S6: OMEGA for the IV route

The following tables give the value of the OMEGA matrices (random components) of the corresponding Between Subject variability and of the shrinkages for the IV route.

| Label | nVc | nCl | nV2 | nV3 | nCl2 | nCl3 |
| --- | --- | --- | --- | --- | --- | --- |
| Omega |  |  |  |  |  |  |
| nVc | 1.401 |  |  |  |  |  |
| nCl | 0.209 | 0.067 |  |  |  |  |
| nV2 | 0.402 | 0.088 | 0.280 |  |  |  |
| nV3 | 0.347 | 0.065 | 0.146 | 0.165 |  |  |
| nCl2 | 0.317 | 0.049 | 0.110 | 0.054 | 0.237 |  |
| nCl3 | 0.335 | 0.066 | 0.078 | 0.109 | 0.128 | 0.214 |
| **BSV%** | **175** | **26.4** | **56.8** | **42.4** | **51.7** | **48.9** |
| Correlation |  |  |  |  |  |  |
| nV | 1.000 |  |  |  |  |  |
| nCl | 0.682 | 1.000 |  |  |  |  |
| nV2 | 0.642 | 0.641 | 1.000 |  |  |  |
| nV3 | 0.720 | 0.611 | 0.678 | 1.000 |  |  |
| nCl2 | 0.550 | 0.391 | 0.427 | 0.274 | 1.000 |  |
| nCl3 | 0.611 | 0.546 | 0.317 | 0.578 | 0.568 | 1.000 |
| **Shrinkage** | **0.11** | **0.16** | **0.29** | **0.25** | **0.45** | **0.42** |

*Inspection of Table S8 indicates that the BSV of clearance is moderate (26.6%) with low shrinkage indicating good individual plasma clearance estimates.*

# Supplementary Table S7: typical values of primary parameters of BP as obtained by the full population model integrating the different modalities of BP administration (IV, IM with different formulations). Associated CV% and SE as measure of precision of thetas estimates were not computed due to the size of the model that would have required several months of computation by a bootstrap method.

| **Parameters** | **Estimate** | **Units** | **Definition** |
| --- | --- | --- | --- |
| tvVc | 106 | mL/kg | Volume of central compartment; All nations/no covariate |
| tvV2 | 46 | mL/kg | Volume of the first peripheral compartment; All nations/no covariate |
| tvV3 | 50 | mL/kg | Volume of the second peripheral compartment; All nations/no covariate |
| tvCl (France) | 481 | mL/kg/h | Plasma clearance; France as reference & covariate for others nations |
| tvCl2 | 126 | mL/kg/h | Clearance of distribution to V2; All nations/no covariate |
| tvCl3 | 25 | mL/kg/h | Clearance of distribution to V3; All nations/no covariate |
| dCldNationcode1 | -0.6833 | Scalar | Covariate Clearance SW IV |
| dCldNationcode2 | -0.1258 | Scalar | Covariate Clearance USA1 |
| dCldNationcode3 | -0.1567 | Scalar | Covariate Clearance JP |
| dCldNationcode11 | -0.3146 | Scalar | Covariate Clearance SW11 BP-PROC & BP-NA |
| dCldNationcode4 | -0.1361 | Scalar | Covariate Clearance USA2 |
| tvCl_SW1 (IV) | 243 | mL/kg | Plasma clearance IV SW1 |
| tvCl_USA1 (IV & BP-PROC) | 424 | mL/kg | Plasma clearance USA1 |
| tvCl_JP (BP-PROC) | 411 | mL/kg | Plasma clearance JP (Bayesian estimate) |
| tvCl_SW11 (BP-PROC & BP-NA) | 351 | mL/kg | Plasma clearance SW11 (Bayesian estimate) |
| tvCl_USA2 (IV) | 420 | mL/kg | Plasma clearance USA2 |
| tvKa1BP-Na | 1.024 | 1/h | first rate constant of absorption for BP-Na |
| tvKa2BP-Na | 0.248 | 1/h | second rate constant of absorption for BP-Na after Tlag |
| tvTlagBP-Na | 0.471 | h | Lag-time between Ka1 and Ka2 for BP-NA |
| **tvFBP-Na** | **89.1** | **%** | **Bioavailability for BP-Na;** |
| tvKaBP-PROC _from Duplocilline | 0.0827 | 1/h | Rate constant of absorption of BP for BP-PROC of Duplocilline |
| tvFBP-PROC _from Duplocilline | 1.532 | Scalar | Bioavailability of BP from BP-PROC for Duplocilline ; ilogit |
| **Bioavailability BP-Proc from Duplocilline** | **82.2** | **%** | **Bioavailability of BP from BP-PROC for Duplocilline** |
| tvKaBP-BENZA _from Duplocilline | 0.0094 | 1/h | Rate constant of absorption of BP for BP-BENZA of Duplocilline |
| tvFBP-BENZA _from Duplocilline | 10.9422 | Scalar | Bioavailability of BP from BP-BENZA for Duplocilline ; ilogit |
| **Bioavailability BP-BENZA from Duplocilline** | **100.0** | **%** | **Bioavailability of BP from BP-BENZA for Duplocilline** |
| tvKaPROC | 0.0466 | 1/h | Rate constant of absorption for BP-PROC (France as reference) |
| tvFBP-PROC | 7.8310 | Scalar | Bioavailability for BP from BP-PROC ; ilogit; France as reference |
| **Bioavailability BP-PROC** | **100.0** | **%** | **Bioavailability for BP from BP-PROC (France)** |
| dKadNationcode11 | 0.0390 | Scalar | Covariate Ka for BP-PROC SW11 |
| dKadNationcode2 | -2.372 | Scalar | Covariate Ka for BP-PROC USA1 |
| dKadNationcode3 | -0.0043 | Scalar | Covariate Ka for BP-PROC Japan |
| dFNationcode11 | -0.0018 | Scalar | Covariate bioavailability ilogit BP-PROC SW11 |
| dFNationcode2 | 0.052 | Scalar | Covariate bioavailability ilogit BP-PROC USA1 |
| dFNationcode3 | 0.423 | Scalar | Covariate bioavailability ilogit BP-PROC Japan |
| tvKaPenethamate_rapid1 | 0.0350 | 1/h | Penethamate: initial rate constant of absorption for site 1 |
| tvKaPenethamate_rapid2 | 0.0223 | 1/h | Penethamate: initial rate constant of absorption for site 2 |
| tvKaPenethamate_rapid3 | 0.0152 | 1/h | Penethamate: initial rate constant of absorption for site 3 |
| tvKaPenethamate_slow1 | 0.0142 | 1/h | Penethamate: Second rate constant of absorption for site 1 |
| tvKaPenethamate_slow2 | 0.1083 | 1/h | Penethamate: Second rate constant of absorption for site 2 |
| tvKaPenethamate_slow3 | 0.0016 | 1/h | Penethamate: Second rate constant of absorption for site 3 |
| tvFPenethamate | 0.7888 | 1/h | Bioavailability of BP from penethamate; ilogit |
| **Bioavailability BP_ from Penethamate** | **68.8** | % | **Bioavailability of BP from penethamate** |
| tvTlag1 | 70.6 | h | delay after the first administration for the switch between penethamate_rapid1 and penethamate_slow1 |
| tvTlag2 | 27.2 | h | delay after the second administration for the switch between penethamate_rapid2 and penethamate_slow2 |
| tvTlag3 | 42.3 | h | delay after the third administration for the switch between penethamate_rapid3 and penethamate_slow3 |
| **Parameters of the Error model** | **Estimate** | **Units** | **Definition** |
| tvCMultStdev | 0.283 | Scalar | CV for the multiplicative component of the residual error; All nations |
| stdev0 | 0.007928 | µg/mL | residual error; additive component ; IV |
| stdev1 | 0.162677 | µg/mL | residual error; additive component ; IM BP-Na |
| stdev2 | 0.006537 | µg/mL | residual error; additive component ; IM Duplocilline |
| stdev3 | 0.004579 | µg/mL | residual error; additive component , IM BP-PROC |
| stdev4 | 0.008068 | µg/mL | residual error; additive component ; IM BP penethamate |

*The clearance of Swedish and Japanese horses that had not received BP by the IV route were estimated in a Bayesian manner. For Japanese horses, plasma clearance was similar to the French, USA1 and USA2 values. For Swedish horses, the clearance of the 8 horses having received BP-PROC and BP-NA was clearly higher (351 mL/kg/h) than those of the other 4 Swedish horses having received BP-NA by IV route (243 mL/kg/h) but lower than those of the other nations (420-480 mL/kg/h).*

**Supplementary Table S8:** secondary parameters estimated with the full model (IV and IM data)

| **Secondary** | **Estimate** |  | **Units** | **Definition** |
| --- | --- | --- | --- | --- |
| ClearanceSW1 | 243 |  | mL/kg/h | Plasma clearance Swedish SW1 (IV) |
| ClearanceSW11 | 351 |  | mL/kg/h | Plasma clearance Swedish SW11 (no IV), Bayesian estimate |
| ClearanceJP | 411 |  | mL/kg/h | Plasma clearance JP (no IV), Bayesian estimate |
| ClearanceUSA1 | 424 |  | mL/kg/h | Plasma clearance USA1 (IV) |
| ClearanceUSA2 | 420 |  | mL/kg/h | Plasma clearance USA2 (IV) |
| tvKe | 4.53 |  | 1/h | Rate constant of elimination of BP from central compartment |
| tvK12 | 1.19 |  | 1/h | Rate constant of exchange of BP between compartment 1 and compartment 2 |
| tvK13 | 0.235 |  | 1/h | Rate constant of exchange of BP between compartment 1 and compartment 3 |
| tvK21 | 2.72 |  | 1/h | Rate constant of exchange of BP between compartment 2 and compartment 1 |
| tvK31 | 0.495 |  | 1/h | Rate constant of exchange of BP between compartment 3 and compartment 1 |
| tvVss | 203 |  | mL/kg | Steady-state volume of distribution |
| tvVarea | 1030 |  | mL/kg | Volume of distribution of the terminal phase |
| tvMRTIV | 0.422 |  | h | Mean Residence Time of BP (IV) |
| tvAlpha | 6.77 |  | 1/h | First slope of distribution |
| tvBeta | 1.93 |  | 1/h | Second slope of distribution |
| tvGamma | 0.467 |  | 1/h | Slope of elimination |
| tvHL_alpha | 0.102 |  | h | Half-life of the first slope of distribution |
| tvHL_Beta | 0.359 |  | h | Half-life of the second slope of distribution |
| tvHL_Gamma | 1.48 |  | h | Terminal half-life |
| tvA | 78 |  | kg/mL | Intercept for the first phase for a dose of 10 mg/kg |
| tvB | 15 |  | kg/mL | Intercept for the second phase for a dose of 10 mg/kg |
| tvC | 0.657 |  | kg/mL | Intercept for the terminal phase for a dose of 10 mg/kg |
| MAT1_BP-Na | 0.976 |  | h | Mean Absorption Time of BP after administration of BP-Na (initial rapid phase of absorption) |
| MAT2_BP-Na | 4.03 |  | h | Mean Absorption Time of BP after administration of BP-Na (second slow phase of absorption after Tlag) |
| **Bioavailability_BP-PROC from Duplocilline** | **82.2** |  | **%** | **Bioavailability of BP from BP-Proc of Duplocilline** |
| **Bioavailability_BP-BENZA from Duplocilline** | **100.0** |  | **%** | **Bioavailability of BP from BP-BENZA of Duplocilline** |
| MAT_BP-PROC from Duplocilline | 12.1 |  | h | Mean Absorption Time of BP after administration of BP-PROC with Duplocilline |
| MAT_BP-BENZA from Duplocilline | 107 |  | h | Mean Absorption Time of BP after administration of BP-BENZA with Duplocilline |
| **Bioavailability_BP-PROC_Fr** | **99.960** |  | **%** | **Bioavailability of BP from BP-Proc of French formulation of BP-Proc** |
| **Bioavailability_BP-PROC_SW11** | **99.960** |  | **%** | **Bioavailability of BP from BP-Proc of Swedish formulation of BP-Proc** |
| **Bioavailability_BP-PROC_USA1** | **99.974** |  | **%** | **Bioavailability of BP from BP-Proc of USA1 formulation of BP-Proc** |
| **Bioavailability_BP-PROC_JP** | **99.999** |  | **%** | **Bioavailability of BP from BP-Proc of JP formulation of BP-Proc** |
| KaSW11_PROC | 0.048 |  | 1/h | rate constant of absorption BP-PROC Swedish |
| KaUSA1_PROC | 0.00435 |  | 1/h | rate constant of absorption BP-PROC USA1 |
| KaJP_PROC | 0.04640 |  | 1/h | rate constant of absorption BP-PROC JP |
| MAT_BP-PROC_France | 21.46 |  | h | Mean Absorption Time of BP after administration of BP-Proc ; French formulation |
| MAT_KaSW11_PROc | 20.64 |  | h | Mean Absorption Time of BP after administration of BP-Proc ; Swedish formulation |
| MAT_KaUSA1_PROc | 230 |  | h | Mean Absorption Time of BP after administration of BPProc ; USA1 formulation |
| MAT_KaJP_PROc | 21.55 |  | h | Mean Absorption Time of BP after administration of BP-Proc ; JP formulation |
| MAT_Penethamate1 | 28.57 |  | h | Mean Absorption Time of BP after the first administration of penethamate |
| MAT_Penethamate2 | 44.80 |  | h | Mean Absorption Time of BP after administration of the second administration of penethamate |
| MAT_Penethamate3 | 65.92 |  | h | Mean Absorption Time of BP after administration the third administration of penethamate |
| **Bioavailabity_Penethamate** | **68.8** |  | **%** | **Bioavailability of BP after administration of penethamate** |

**Supplementary Table S9:** OMEGA for BP-PROC

| Label | nKaPROC | nFPROC |
| --- | --- | --- |
| nKaPROC | 0.110 |  |
| nFPROC (ilogit) | 0.524 | 3.662 |
| **BSV %** | **34.0** | **616** |
| nKaPROC | 1.000 |  |
| nFPROC | 0.826 | 1.000 |
| **Shrinkage** | **0.24** | **0.37** |

*Inspection of Table S9 indicates that the BSV of Ka for BP-PROC is moderate (34%) with low shrinkage (0.24) indicating good individual estimates of absorption rates. For bioavailability, the BSV indicated is that of ilogit estimates but the BSV of the bioavailability of BP-PROC is very low given the fact that bioavailability is total for all formulations.*

**Supplementary Table S10:** OMEGA for BP-Na

| Label | nFPeniNa | nTlagPeni | nKa1PeniNA | nKa2PeniNA |
| --- | --- | --- | --- | --- |
| nFPeniNa | 0.031 |  |  |  |
| nTlagPeni | 0.001 | 0.044 |  |  |
| nKa1PeniNA | 0.002 | -0.073 | 0.805 |  |
| nKa2PeniNA | 0.006 | 0.002 | -0.169 | 0.044 |
| **BSV %** | **17.7** | **21.3** | **111.2** | **21.3** |
| nFPeniNa | 1.000 |  |  |  |
| nTlagPeni | 0.020 | 1.000 |  |  |
| nKa1PeniNA | 0.014 | -0.386 | 1.000 |  |
| nKa2PeniNA | 0.162 | 0.038 | -0.894 | 1.000 |
| **Shrinkage** | **0.62** | **0.73** | **0.55** | **0.62** |

*Inspection of table S10 indicates that the BSV of bioavailability (17.7%) and that of Ka2, (21.3%) for BP-Na are moderate but with rather high shrinkages (>0.5) indicating that the individual estimates are not robustly estimated*

**Supplementary table S11:** OMEGA for Duplocilline (combination of BP-PROC+BP-BENZA)

| Label | nKaBP_PROC | nKaBP-BENZA | nFBP-PROC (ilogit) | nFBP-BENZA (ilogit) |
| --- | --- | --- | --- | --- |
| nKaBP_PROC | 0.127 |  |  |  |
| nKaBP-BENZA | 0.180 | 0.616 |  |  |
| nFBP-PROC | 0.082 | 0.779 | 2.163 |  |
| nFBP-BENZA | 0.201 | 0.589 | 1.344 | 1.161 |
| **BSV %** | **36.7** | **92.3** | **277** | **148** |
| nKaBP_PROC | 1.000 |  |  |  |
| nKaBP-BENZA | 0.644 | 1.000 |  |  |
| nFBP-PROC (ilogit) | 0.157 | 0.674 | 1.000 |  |
| nFBP-BENZA (ilogit) | 0.525 | 0.696 | 0.848 | 1.000 |
| **Shrinkage** | **0.67** | **0.64** | **0.64** | **0.67** |

*Inspection of Table S11 indicates that the BSV of Ka for BP-PROC is moderate (-36.7 %) but that of Ka for BP benzathine clearly higher (92.3%) with rather high shrinkages (>0.5) for both ka indicating that the individual estimates are not very robust. The BSVs for bioavailability are those of the ilogit estimators and are not directly interpretable*.

**Supplementary Table S12:** OMEGA for Penethamate

| label | nKaPenethamate_rapid 1 | nKaPenethamate_slow1 | nKaPenethamate_rapid2 | nKaPenethamate_slow2 | nKaPenethamate_rapid3 | nKaPenethamate_slow3 | nFPenethamate |
| --- | --- | --- | --- | --- | --- | --- | --- |
| nKaPenethamate_rapid1 | 1.216 |  |  |  |  |  |  |
| nKaPenethamate_slow1 | 0.248 | 0.051 |  |  |  |  |  |
| nKaPenethamate_rapid2 | 0.000 | 0.000 | 0.094 |  |  |  |  |
| nKaPenethamate_slow2 | 0.000 | 0.000 | 0.045 | 0.021 |  |  |  |
| nKaPenethamate_rapid3 | 0.000 | 0.000 | 0.000 | 0.000 | 3.146 |  |  |
| nKaPenethamate_slow3 | 0.000 | 0.000 | 0.000 | 0.000 | 0.310 | 0.031 |  |
| nFPenethamate (ilogit) | 0.000 | 0.000 | 0.000 | 0.000 | 0.000 | 0.000 | 0.005 |
| **BSV %** | **154** | **22.8** | **31.5** | **14.6** | **472** | **17.6** | **6.9** |
| nKaPenethamate_rapid1 | 1.000 |  |  |  |  |  |  |
| nKaPenethamate_slow1 | 0.999 | 1.000 |  |  |  |  |  |
| nKaPenethamate_rapid2 | 0.000 | 0.000 | 1.000 |  |  |  |  |
| nKaPenethamate_slow2 | 0.000 | 0.000 | 1.000 | 1.000 |  |  |  |
| nKaPenethamate_rapid3 | 0.000 | 0.000 | 0.000 | 0.000 | 1.000 |  |  |
| nKaPenethamate_slow3 | 0.000 | 0.000 | 0.000 | 0.000 | 0.999 | 1.000 |  |
| nFPenethamate | 0.000 | 0.000 | 0.000 | 0.000 | 0.000 | 0.000 | 1.000 |
| **Shrinkage** | **0.73** | **0.73** | **0.82** | **0.82** | **0.68** | **0.68** | **0.88** |

*The adjustments for penethamate were difficult requiring to consider each site separately for the two rate constant of absorption (initially fast then slow) and the confidence in these estimators is limited. However, this has no implication for the rest of the project (no PK/PD cutoff for penethamate).*

**Supplementary Table S13**: Quantiles of the horse population able to achieve a PK/PD index of 72 h for fAUC/MIC (0-72h) (units in h) for different MIC (0.0625 to 2.0 mg/L) when BP is administered as BP-PROC over 72 h. Plot of quantiles 90th vs MIC in figures 61 to 64. Selected associated plots of PTA are also given.

PK/PD cutoff were computed for France, Sweden, USA1 and Japan.

| MIC (mg/L) | Dose (µg/kg) | Interval (h) | Nation | Mean | CV  Percent | Q95% | Q90% | Q75% | Q50% |
| --- | --- | --- | --- | --- | --- | --- | --- | --- | --- |
| 0.0625 | 12360 | 24 | France | 423 | 28.70 | 254 | 282 | 336 | 406 |
| 0.0625 | 12360 | 24 | Japan | 491 | 28.31 | 297 | 331 | 392 | 474 |
| 0.0625 | 12360 | 24 | Sweden | 585 | 28.56 | 353 | 391 | 465 | 564 |
| 0.0625 | 12360 | 24 | **USA1** | 109 | 40.62 | 53 | **61** | 78 | 102 |
| 0.0625 | 24720 | 24 | France | 847 | 27.89 | 510 | 568 | 678 | 819 |
| 0.0625 | 24720 | 24 | Japan | 993 | 28.43 | 594 | 666 | 791 | 956 |
| 0.0625 | 24720 | 24 | Sweden | 1174 | 28.34 | 710 | 794 | 939 | 1129 |
| 0.0625 | 24720 | 24 | USA1USA1 | 221 | 40.05 | 105 | 121 | 157 | 207 |
| 0.0625 | 12360 | 12 | France | 785 | 29.24 | 463 | 519 | 623 | 758 |
| 0.0625 | 12360 | 12 | Japan | 920 | 29.09 | 550 | 616 | 730 | 883 |
| 0.0625 | 12360 | 12 | Sweden | 1089 | 29.71 | 639 | 715 | 862 | 1042 |
| 0.0625 | 12360 | 12 | USA1USA1 | 194 | 40.25 | 92.6 | 107 | 139 | 181 |
| 0.1000 | 12360 | 24 | France | 264 | 28.70 | 159 | 176 | 210 | 254 |
| 0.1000 | 12360 | 24 | Japan | 307 | 28.31 | 185 | 207 | 245 | 297 |
| 0.1000 | 12360 | 24 | Sweden | 366 | 28.56 | 221 | 245 | 291 | 353 |
| 0.1000 | 12360 | 24 | USA1 | 68.4 | 40.62 | 32.9 | 37.9 | 48.6 | 63.7 |
| 0.1000 | 24720 | 24 | France | 529 | 27.89 | 319 | 355 | 424 | 512 |
| 0.1000 | 24720 | 24 | Japan | 621 | 28.43 | 372 | 416 | 495 | 598 |
| 0.1000 | 24720 | 24 | Sweden | 734 | 28.34 | 444 | 496 | 587 | 706 |
| 0.1000 | 24720 | 24 | USA1 | 138 | 40.05 | 65.8 | 75.5 | 98.2 | 129 |
| 0.1000 | 12360 | 12 | France | 491 | 29.24 | 289 | 324 | 389 | 474 |
| 0.1000 | 12360 | 12 | Japan | 575 | 29.09 | 344 | 385 | 456 | 552 |
| 0.1000 | 12360 | 12 | Sweden | 681 | 29.71 | 400 | 447 | 539 | 652 |
| 0.1000 | 12360 | 12 | USA1 | 121 | 40.25 | 57.9 | 67.2 | 86.6 | 113 |
| 0.1250 | 12360 | 24 | France | 211 | 28.70 | 127 | 141 | 168 | 203 |
| 0.1250 | 12360 | 24 | Japan | 246 | 28.31 | 148 | 165 | 196 | 237 |
| 0.1250 | 12360 | 24 | Sweden | 293 | 28.56 | 177 | 196 | 232 | 282 |
| 0.1250 | 12360 | 24 | USA1 | 54.8 | 40.62 | 26.3 | 30.3 | 38.8 | 51.0 |
| 0.1250 | 24720 | 24 | France | 424 | 27.89 | 255 | 284 | 339 | 410 |
| 0.1250 | 24720 | 24 | Japan | 496 | 28.43 | 297 | 333 | 396 | 478 |
| 0.1250 | 24720 | 24 | Sweden | 587 | 28.34 | 355 | 397 | 470 | 565 |
| 0.1250 | 24720 | 24 | USA1 | 110 | 40.05 | 52.6 | 60.4 | 78.6 | 104 |
| 0.1250 | 12360 | 12 | France | 393 | 29.24 | 232 | 260 | 312 | 379 |
| 0.1250 | 12360 | 12 | Japan | 460 | 29.09 | 275 | 308 | 365 | 442 |
| 0.1250 | 12360 | 12 | Sweden | 545 | 29.71 | 320 | 358 | 431 | 521 |
| 0.1250 | 12360 | 12 | USA1 | 97.0 | 40.25 | 46.3 | 53.8 | 69.3 | 90.4 |
| 0.2500 | **12360** | **24** | **France** | 106 | 28.70 | 63.4 | **70.4** | 84.0 | 102 |
| 0.2500 | **12360** | **24** | **Japan** | 123 | 28.31 | 74.2 | **82.7** | 97.9 | 119 |
| 0.2500 | **12360** | **24** | **Sweden** | 146 | 28.56 | 88.4 | **97.9** | 116 | 141 |
| 0.2500 | **12360** | **24** | **USA1** | 27.4 | 40.62 | 13.2 | **15.2** | 19.4 | 25.5 |
| 0.2500 | 24720 | 24 | France | 212 | 27.89 | 128 | 142 | 170 | 205 |
| 0.2500 | 24720 | 24 | Japan | 248 | 28.43 | 149 | 166 | 198 | 239 |
| 0.2500 | 24720 | 24 | Sweden | 294 | 28.34 | 178 | 198 | 235 | 282 |
| 0.2500 | 24720 | 24 | USA1 | 55.2 | 40.05 | 26.3 | 30.2 | 39.3 | 51.8 |
| 0.2500 | 12360 | 12 | France | 196 | 29.24 | 116 | 130 | 156 | 190 |
| 0.2500 | 12360 | 12 | Japan | 230 | 29.09 | 138 | 154 | 182 | 221 |
| 0.2500 | 12360 | 12 | Sweden | 272 | 29.71 | 160 | 179 | 215 | 261 |
| 0.2500 | 12360 | 12 | USA1 | 48.5 | 40.25 | 23.2 | 26.9 | 34.6 | 45.2 |
| 0.3750 | 12360 | 24 | France | 70.5 | 28.70 | 42.3 | 46.9 | 56.0 | 67.8 |
| 0.3750 | 12360 | 24 | Japan | 81.8 | 28.31 | 49.5 | 55.1 | 65.3 | 79.1 |
| 0.3750 | 12360 | 24 | Sweden | 97.6 | 28.56 | 58.9 | 65.2 | 77.5 | 94.0 |
| 0.3750 | 12360 | 24 | USA1 | 18.3 | 40.62 | 8.8 | 10.1 | 12.9 | 17.0 |
| 0.3750 | 24720 | 24 | France | 141 | 27.89 | 85.1 | 94.7 | 113 | 137 |
| 0.3750 | 24720 | 24 | Japan | 165 | 28.43 | 99.1 | 111 | 132 | 159 |
| 0.3750 | 24720 | 24 | Sweden | 196 | 28.34 | 118 | 132 | 157 | 188 |
| 0.3750 | 24720 | 24 | USA1 | 36.8 | 40.05 | 17.5 | 20.1 | 26.2 | 34.5 |
| 0.3750 | 12360 | 12 | France | 131 | 29.24 | 77.2 | 86.5 | 104 | 126 |
| 0.3750 | 12360 | 12 | Japan | 153 | 29.09 | 91.7 | 103 | 122 | 147 |
| 0.3750 | 12360 | 12 | Sweden | 182 | 29.71 | 107 | 119 | 144 | 174 |
| 0.3750 | 12360 | 12 | USA1 | 32.3 | 40.25 | 15.4 | 17.9 | 23.1 | 30.1 |
| 0.5000 | 12360 | 24 | France | 52.8 | 28.70 | 31.7 | 35.2 | 42.0 | 50.8 |
| 0.5000 | 12360 | 24 | Japan | 61.4 | 28.31 | 37.1 | 41.3 | 49.0 | 59.3 |
| 0.5000 | 12360 | 24 | Sweden | 73.2 | 28.56 | 44.2 | 48.9 | 58.1 | 70.5 |
| 0.5000 | 12360 | 24 | USA1 | 13.7 | 40.62 | 6.6 | 7.6 | 9.7 | 12.7 |
| 0.5000 | 24720 | 24 | France | 106 | 27.89 | 63.8 | 71.0 | 84.8 | 102 |
| 0.5000 | 24720 | 24 | Japan | 124 | 28.43 | 74.3 | 83.2 | 98.9 | 120 |
| 0.5000 | 24720 | 24 | Sweden | 147 | 28.34 | 88.8 | 99.2 | 117.5 | 141 |
| 0.5000 | 24720 | 24 | USA1 | 28 | 40.05 | 13.2 | 15.1 | 19.6 | 25.9 |
| 0.5000 | 12360 | 12 | France | 98.2 | 29.24 | 57.9 | 64.9 | 77.9 | 94.8 |
| 0.5000 | 12360 | 12 | Japan | 115 | 29.09 | 68.8 | 77.0 | 91.2 | 110 |
| 0.5000 | 12360 | 12 | Sweden | 136 | 29.71 | 80.0 | 89.4 | 108 | 130 |
| 0.5000 | 12360 | 12 | USA1 | 24.2 | 40.25 | 11.6 | 13.4 | 17.3 | 22.6 |
| 1.0000 | 12360 | 24 | France | 26.4 | 28.70 | 15.9 | 17.6 | 21.0 | 25.4 |
| 1.0000 | 12360 | 24 | Japan | 30.7 | 28.31 | 18.5 | 20.7 | 24.5 | 29.7 |
| 1.0000 | 12360 | 24 | Sweden | 36.6 | 28.56 | 22.1 | 24.5 | 29.1 | 35.3 |
| 1.0000 | 12360 | 24 | USA1 | 6.8 | 40.62 | 3.3 | 3.8 | 4.9 | 6.4 |
| 1.0000 | 24720 | 24 | France | 52.9 | 27.89 | 31.9 | 35.5 | 42.4 | 51.2 |
| 1.0000 | 24720 | 24 | Japan | 62.1 | 28.43 | 37.2 | 41.6 | 49.5 | 59.8 |
| 1.0000 | 24720 | 24 | Sweden | 73.4 | 28.34 | 44.4 | 49.6 | 58.7 | 70.6 |
| 1.0000 | 24720 | 24 | USA1 | 13.8 | 40.05 | 6.6 | 7.5 | 9.8 | 12.9 |
| 1.0000 | 12360 | 12 | France | 49.1 | 29.24 | 28.9 | 32.4 | 38.9 | 47.4 |
| 1.0000 | 12360 | 12 | Japan | 57.5 | 29.09 | 34.4 | 38.5 | 45.6 | 55.2 |
| 1.0000 | 12360 | 12 | Sweden | 68.1 | 29.71 | 40.0 | 44.7 | 53.9 | 65.2 |
| 1.0000 | 12360 | 12 | USA1 | 12.1 | 40.25 | 5.8 | 6.7 | 8.7 | 11.3 |
| 2.0000 | 12360 | 24 | France | 13.2 | 28.7 | 7.9 | 8.8 | 10.5 | 12.7 |
| 2.0000 | 12360 | 24 | Japan | 15.3 | 28.3 | 9.3 | 10.3 | 12.2 | 14.8 |
| 2.0000 | 12360 | 24 | Sweden | 18.3 | 28.6 | 11.0 | 12.2 | 14.5 | 17.6 |
| 2.0000 | 12360 | 24 | USA1 | 3.4 | 40.6 | 1.6 | 1.9 | 2.4 | 3.2 |
| 2.0000 | 24720 | 24 | France | 26.5 | 27.9 | 16.0 | 17.8 | 21.2 | 25.6 |
| 2.0000 | 24720 | 24 | Japan | 31.0 | 28.4 | 18.6 | 20.8 | 24.7 | 29.9 |
| 2.0000 | 24720 | 24 | Sweden | 36.7 | 28.3 | 22.2 | 24.8 | 29.4 | 35.3 |
| 2.0000 | 24720 | 24 | USA1 | 6.9 | 40.0 | 3.3 | 3.8 | 4.9 | 6.5 |
| 2.0000 | 12360 | 12 | France | 24.5 | 29.2 | 14.5 | 16.2 | 19.5 | 23.7 |
| 2.0000 | 12360 | 12 | Japan | 28.7 | 29.1 | 17.2 | 19.2 | 22.8 | 27.6 |
| 2.0000 | 12360 | 12 | Sweden | 34.0 | 29.7 | 20.0 | 22.4 | 26.9 | 32.6 |
| 2.0000 | 12360 | 12 | USA1 | 6.1 | 40.2 | 2.9 | 3.4 | 4.3 | 5.6 |

*Inspection of table S13 shows that the PK/PD cutoff is of 0.25 mg/L with three nations/formulations able to reach it (France, Sweden and Japan). For France, Q90th is border line (70.4h rather than a PDT of 72h) On the other hand, the USA1 formulation was unable to achieve a PK/PD index of 72 hours. This was expected after inspection of the plasma concentrations.*

**Supplementary Table S14:** Quantiles of the horse population able to achieve a PK/PD index of 96h for fAUC/MIC (0-96h) (units in h) for different MIC (0.0625 to 2.0 mg/L) when BP is administered as Duplocilline© (a combination of BP-PROC and BP-BENZ) at dose of 12360 µg/kg twice at 48 h interval. The duration of treatment is 96 rather than 72h because the SPC indicate an interval of administration of 48h assuming implicitly a total treatment duration of 96h.

| **MIC (mg/L)** | **Dose (µg/kg** | **Interval (h)** | **Nation** | **Mean (h)** | **CVPercent** | **Q95** | **Q90** | **Q75** | **Q50** |
| --- | --- | --- | --- | --- | --- | --- | --- | --- | --- |
| 0.0625 | 12360 | 48 | France | 208 | 42.00 | 78.7 | 101 | 144 | 201 |
| 0.1000 | 12360 | 48 | France | 130 | 42.00 | 49.2 | 63.1 | 89.8 | 126 |
| 0.1250 | 12360 | 48 | France | 104 | 42.00 | 39.3 | 50.5 | 71.9 | 101 |
| **0.2500** | 12360 | 48 | France | 52.1 | 42.00 | 19.7 | **25.3** | 35.9 | 50.3 |
| 0.3750 | 12360 | 48 | France | 34.8 | 42.00 | 13.1 | 16.8 | 24.0 | 33.6 |
| 0.5000 | 12360 | 48 | France | 26.1 | 42.00 | 9.8 | 12.6 | 18.0 | 25.2 |
| 1.0000 | 12360 | 48 | France | 13.0 | 42.00 | 4.9 | 6.3 | 9.0 | 12.6 |
| 2.0000 | 12360 | 48 | France | 6.5 | 42.00 | 2.5 | 3.2 | 4.5 | 6.3 |

*Inspection of table S14 shows that the PK/PD cutoff of 0.25 mg/L is not achievable with Duplocilline© (Q90% of 25.3 h i.e. an average BP plasma concentration over 96 h 3.8 times lower than this PK/PD target of 96h). For a MIC of 0.0625 mg/L, the Q90% is of 101 h i.e. just above the PK/PD target of 96h and as for BP-PROC USA1, this is likely sufficient for Streptococcus sp having a MIC90 ≤ 0.06 mg/L.*

**Supplementary Table S15:** Quantiles of the horse population able to achieve a PK/PD index of 72h for fAUC/MIC (0-72h) (units in h) for different MIC (0.065 to 2.0 mg/L) when BP is administered as penethamate (Penetavet©) at initial dose of 12360 µg/kg then at dose of 6180 µg/kg twice at 24 h interval.

| **MIC (mg/L)** | **Dosage regimen** | **Nation** | **Mean** | **CVPercent** | **Q95** | **Q90** | **Q75** | **Q50** |
| --- | --- | --- | --- | --- | --- | --- | --- | --- |
| 0.0625 | 12360 then 6180 twice at 24h intervals | France | 173 | 33.24 | 92.4 | 106 | 133 | 165 |
| 0.1 | 12361 then 6180 twice at 24h intervals | France | 108 | 33.24 | 57.8 | 66.4 | 83.2 | 103 |
| 0.125 | 12362 then 6180 twice at 24h intervals | France | 86.8 | 33.24 | 46.2 | 53.1 | 66.5 | 82.7 |
| **0.25** | 12363 then 6180 twice at 24h intervals | France | 43.4 | 33.24 | 23.1 | **26.5** | 33.3 | 41.4 |
| 0.375 | 12364 then 6180 twice at 24h intervals | France | 28.9 | 33.24 | 15.4 | 17.7 | 22.2 | 27.6 |
| 0.5 | 12365 then 6180 twice at 24h intervals | France | 21.7 | 33.24 | 11.6 | 13.3 | 16.6 | 20.7 |
| 1 | 12366 then 6180 twice at 24h intervals | France | 10.8 | 33.24 | 5.8 | 6.6 | 8.3 | 10.3 |
| 2 | 12367 then 6180 twice at 24h intervals | France | 5.4 | 33.24 | 2.9 | 3.3 | 4.2 | 5.2 |

*Inspection of table S15 shows that the PK/PD cutoff of 0.25 mg/L is not achievable with Penetavet© (Q90% of 26.5 h i.e. an average BP plasma concentration over 72h three times lower than 0.25 mg/L). The same conclusion holds for a MIC of 0.125 mg/L with a PK/PD value of 53h. For a MIC of 0.0625 mg/L, the Q90% is of 106 h i.e. above the PK/PD target of 72h and this is sufficient for Streptococcus sp having a MIC90 ≤ 0.06 mg/L.*

**Supplementary Table S16***:* Cumulative times (h) spent above the MIC for different formulation of BP-PROC at different dosage regimen for MIC ranging from 0.0625 to 2 mg/L over the 72 h of a treatment duration. Target is 30 or 40% of the dosing interval i.e. a cumulated duration of 21.3 or 28.8 h over 72h.

| **MIC (mg/L)** | **Dose (µg/kg)** | **Interval (h)** | **Nation** | **Mean** | **CV**  **Percent** | **Q95% (h)** | **Q90% (h)** | **Q75% (h)** | **Q50% (h)** |
| --- | --- | --- | --- | --- | --- | --- | --- | --- | --- |
|  |  |  |  |  |  |  |  |  |  |
| 0.0625 | 12360 | 24 | France | 71.9 | 0.17 | 71.8 | 71.8 | 71.9 | 72.0 |
| 0.0625 | 12360 | 24 | Japan | 71.9 | 0.22 | 71.7 | 71.8 | 71.9 | 72.0 |
| 0.0625 | 12360 | 24 | Sweden | 71.9 | 0.33 | 71.8 | 71.9 | 71.9 | 72.0 |
| 0.0625 | **12360** | **24** | **USA1** | 48.5 | 35.17 | 22.9 | **23.8** | 47.1 | 47.9 |
| 0.0625 | 24720 | 24 | France | 72.0 | 0.09 | 71.9 | 71.9 | 72.0 | 72.0 |
| 0.0625 | 24720 | 24 | Japan | 72.0 | 0.24 | 71.9 | 71.9 | 72.0 | 72.0 |
| 0.0625 | 24720 | 24 | Sweden | 72.0 | 0.08 | 71.9 | 71.9 | 72.0 | 72.0 |
| 0.0625 | 24720 | 24 | USA1 | 67.7 | 13.10 | 47.8 | 47.9 | 70.7 | 71.6 |
| 0.0625 | 12360 | 12 | France | 71.9 | 0.31 | 71.7 | 71.8 | 71.9 | 72.0 |
| 0.0625 | 12360 | 12 | Japan | 71.9 | 0.19 | 71.8 | 71.8 | 71.9 | 72.0 |
| 0.0625 | 12360 | 12 | Sweden | 71.9 | 0.23 | 71.8 | 71.8 | 71.9 | 72.0 |
| 0.0625 | 12360 | 12 | USA1 | 60.6 | 14.28 | 47.4 | 47.9 | 59.4 | 59.9 |
| 0.100 | 12360 | 24 | France | 71.7 | 1.80 | 71.1 | 71.6 | 71.9 | 71.9 |
| 0.100 | 12360 | 24 | Japan | 71.8 | 0.80 | 71.5 | 71.7 | 71.9 | 71.9 |
| 0.100 | 12360 | 24 | Sweden | 71.9 | 0.76 | 71.6 | 71.7 | 71.9 | 71.9 |
| 0.100 | 12360 | 24 | USA1 | 27.9 | 71.28 | 0.0 | 0.0 | 9.9 | 23.9 |
| 0.100 | 24720 | 24 | France | 71.9 | 0.28 | 71.8 | 71.9 | 71.9 | 72.0 |
| 0.100 | 24720 | 24 | Japan | 71.9 | 0.42 | 71.8 | 71.9 | 71.9 | 72.0 |
| 0.100 | 24720 | 24 | Sweden | 71.9 | 0.13 | 71.8 | 71.9 | 71.9 | 72.0 |
| 0.100 | 24720 | 24 | USA1 | 56.9 | 26.34 | 23.9 | 46.5 | 47.8 | 48.0 |
| 0.100 | 12360 | 12 | France | 71.9 | 0.80 | 71.5 | 71.7 | 71.9 | 71.9 |
| 0.100 | 12360 | 12 | Japan | 71.9 | 0.43 | 71.6 | 71.7 | 71.9 | 71.9 |
| 0.100 | 12360 | 12 | Sweden | 71.9 | 0.46 | 71.6 | 71.7 | 71.9 | 71.9 |
| 0.100 | 12360 | 12 | USA1 | 49.9 | 25.22 | 23.8 | 35.5 | 47.2 | 48.0 |
| 0.125 | 12360 | 24 | France | 70.8 | 4.32 | 65.3 | 68.4 | 71.5 | 71.9 |
| 0.125 | 12360 | 24 | Japan | 71.5 | 2.31 | 69.8 | 71.3 | 71.8 | 71.9 |
| 0.125 | 12360 | 24 | Sweden | 71.7 | 1.49 | 71.3 | 71.6 | 71.8 | 71.9 |
| 0.125 | 12360 | 24 | USA1 | 17.8 | 103 | 0.0 | 0.0 | 0.0 | 20.7 |
| 0.125 | 24720 | 24 | France | 71.9 | 0.50 | 71.7 | 71.8 | 71.9 | 72.0 |
| 0.125 | 24720 | 24 | Japan | 71.9 | 0.53 | 71.7 | 71.8 | 71.9 | 72.0 |
| 0.125 | 24720 | 24 | Sweden | 71.9 | 0.19 | 71.8 | 71.9 | 71.9 | 72.0 |
| 0.125 | 24720 | 24 | USA1 | 49.0 | 34.64 | 23.1 | 23.8 | 47.1 | 47.9 |
| 0.125 | 12360 | 12 | France | 71.8 | 1.27 | 71.3 | 71.6 | 71.8 | 71.9 |
| 0.125 | 12360 | 12 | Japan | 71.8 | 0.69 | 71.4 | 71.6 | 71.8 | 71.9 |
| 0.125 | 12360 | 12 | Sweden | 71.8 | 0.74 | 71.4 | 71.7 | 71.8 | 71.9 |
| 0.125 | 12360 | 12 | USA1 | 42.8 | 35.41 | 11.7 | 23.5 | 35.7 | 47.6 |
| **0.250** | **12360** | **24** | **France** | 51.5 | 28.32 | 24.0 | **31.4** | 42.5 | 53.8 |
| **0.250** | **12360** | **24** | **Japan** | 58.9 | 20.22 | 35.4 | **42.4** | 52.6 | 61.9 |
| **0.250** | **12360** | **24** | **Sweden** | 64.7 | 13.47 | 46.8 | **52.5** | 60.8 | 67.8 |
| **0.250** | **12360** | **24** | **USA1** | 1.1 | 476 | 0.0 | **0.0** | 0.0 | 0.0 |
| 0.250 | 24720 | 24 | France | 70.8 | 4.48 | 64.7 | 68.1 | 71.6 | 71.9 |
| 0.250 | 24720 | 24 | Japan | 71.5 | 2.52 | 69.9 | 71.3 | 71.8 | 71.9 |
| 0.250 | 24720 | 24 | Sweden | 71.7 | 1.46 | 71.3 | 71.6 | 71.8 | 71.9 |
| 0.250 | 24720 | 24 | USA1 | 18.3 | 101 | 0.0 | 0.0 | 0.0 | 22.7 |
| 0.250 | 12360 | 12 | France | 69.3 | 6.80 | 59.7 | 60.0 | 68.9 | 71.7 |
| 0.250 | 12360 | 12 | Japan | 70.6 | 4.59 | 60.0 | 67.5 | 71.3 | 71.8 |
| 0.250 | 12360 | 12 | Sweden | 71.2 | 3.31 | 68.1 | 70.7 | 71.6 | 71.8 |
| 0.250 | 12360 | 12 | USA1 | 14.3 | 111 | 0.0 | 0.0 | 0.0 | 11.5 |
| **0.375** | **12360** | **24** | **France** | 28.3 | 57.81 | 0.0 | 4.7 | **16.4** | 28.5 |
| **0.375** | **12360** | **24** | **Japan** | 37.2 | 44.30 | 6.8 | 14.7 | **26.0** | 38.3 |
| **0.375** | **12360** | **24** | **Sweden** | 47.1 | 32.58 | 19.4 | 26.1 | **37.7** | 48.8 |
| **0.375** | **12360** | **24** | **USA1** | 0.1 | 1766 | 0.0 | 0.0 | **0.0** | 0.0 |
| 0.375 | 24720 | 24 | France | 63.7 | 14.87 | 44.6 | 50.3 | 59.5 | 67.1 |
| 0.375 | 24720 | 24 | Japan | 67.8 | 9.47 | 54.1 | 59.4 | 66.3 | 70.8 |
| 0.375 | 24720 | 24 | Sweden | 70.1 | 5.83 | 61.9 | 65.9 | 70.5 | 71.8 |
| 0.375 | 24720 | 24 | USA1 | 4.7 | 227 | 0.0 | 0.0 | 0.0 | 0.0 |
| 0.375 | 12360 | 12 | France | 62.5 | 14.87 | 45.1 | 52.2 | 59.0 | 64.3 |
| 0.375 | 12360 | 12 | Japan | 65.8 | 10.42 | 54.8 | 58.5 | 59.9 | 68.2 |
| 0.375 | 12360 | 12 | Sweden | 68.4 | 8.00 | 59.1 | 59.8 | 66.7 | 71.4 |
| 0.375 | 12360 | 12 | USA1 | 3.3 | 255 | 0.0 | 0.0 | 0.0 | 0.0 |
| **0.500** | **12360** | **24** | **France** | 13.8 | 95.65 | 0.0 | 0.0 | **0.0** | 11.4 |
| **0.500** | **12360** | **24** | **Japan** | 20.8 | 73.38 | 0.0 | 0.0 | **7.6** | 20.1 |
| **0.500** | **12360** | **24** | **Sweden** | 30.5 | 53.41 | 0.0 | 6.7 | **18.9** | 31.2 |
| **0.500** | **12360** | **24** | **USA1** | 0.0 | 6910 | 0.0 | 0.0 | **0.0** | 0.0 |
| **0.500** | **24720** | **24** | **France** | 51.7 | 27.78 | 24.6 | 31.6 | **43.1** | 54.1 |
| **0.500** | **24720** | **24** | **Japan** | 59.2 | 19.80 | 35.7 | 43.4 | **53.0** | 62.1 |
| **0.500** | **24720** | **24** | **Sweden** | 64.9 | 13.14 | 47.2 | 52.9 | **61.5** | 67.9 |
| 0.500 | 24720 | 24 | USA1 | 1.0 | 500 | 0.0 | 0.0 | 0.0 | 0.0 |
| **0.500** | **12360** | **12** | **France** | 52.0 | 29.68 | 18.5 | 29.6 | **45.2** | 56.3 |
| **0.500** | **12360** | **12** | **Japan** | 58.7 | 19.34 | 37.4 | 44.9 | **54.9** | 59.9 |
| **0.500** | **12360** | **12** | **Sweden** | 63.5 | 13.83 | 47.5 | 54.3 | **59.6** | 65.4 |
| 0.500 | 12360 | 12 | USA1 | 0.7 | 546 | 0.0 | 0.0 | 0.0 | 0.0 |
| 1.000 | 12360 | 24 | France | 0.0 |  | 0.0 | 0.0 | 0.0 | 0.0 |
| 1.000 | 12360 | 24 | Japan | 0.0 |  | 0.0 | 0.0 | 0.0 | 0.0 |
| 1.000 | 12360 | 24 | Sweden | 0.0 |  | 0.0 | 0.0 | 0.0 | 0.0 |
| 1.000 | 12360 | 24 | USA1 | 0.0 |  | 0.0 | 0.0 | 0.0 | 0.0 |
| 1.000 | 24720 | 24 | France | 0.0 |  | 0.0 | 0.0 | 0.0 | 0.0 |
| 1.000 | 24720 | 24 | Japan | 0.0 |  | 0.0 | 0.0 | 0.0 | 0.0 |
| 1.000 | 24720 | 24 | Sweden | 0.0 |  | 0.0 | 0.0 | 0.0 | 0.0 |
| 1.000 | 24720 | 24 | USA1 | 0.0 |  | 0.0 | 0.0 | 0.0 | 0.0 |
| 1.000 | 12360 | 12 | France | 0.0 |  | 0.0 | 0.0 | 0.0 | 0.0 |
| 1.000 | 12360 | 12 | Japan | 0.0 |  | 0.0 | 0.0 | 0.0 | 0.0 |
| 1.000 | 12360 | 12 | Sweden | 0.0 |  | 0.0 | 0.0 | 0.0 | 0.0 |
| 1.000 | 12360 | 12 | USA1 | 0.0 |  | 0.0 | 0.0 | 0.0 | 0.0 |
| 2.000 | 12360 | 24 | France | 0.0 |  | 0.0 | 0.0 | 0.0 | 0.0 |
| 2.000 | 12360 | 24 | Japan | 0.0 | 5101 | 0.0 | 0.0 | 0.0 | 0.0 |
| 2.000 | 12360 | 24 | Sweden | 0.0 | 1640 | 0.0 | 0.0 | 0.0 | 0.0 |
| 2.000 | 12360 | 24 | USA1 | 0.0 |  | 0.0 | 0.0 | 0.0 | 0.0 |
| 2.000 | 24720 | 24 | France | 0.4 | 479 | 0.0 | 0.0 | 0.0 | 0.0 |
| 2.000 | 24720 | 24 | Japan | 1.4 | 299 | 0.0 | 0.0 | 0.0 | 0.0 |
| 2.000 | 24720 | 24 | Sweden | 3.3 | 201 | 0.0 | 0.0 | 0.0 | 0.0 |
| 2.000 | 24720 | 24 | USA1 | 0.0 |  | 0.0 | 0.0 | 0.0 | 0.0 |
| 2.000 | 12360 | 12 | France | 0.1 | 1417 | 0.0 | 0.0 | 0.0 | 0.0 |
| 2.000 | 12360 | 12 | Japan | 0.3 | 748 | 0.0 | 0.0 | 0.0 | 0.0 |
| 2.000 | 12360 | 12 | Sweden | 1.1 | 399 | 0.0 | 0.0 | 0.0 | 0.0 |
| 2.000 | 12360 | 12 | USA1 | 0.0 |  | 0.0 | 0.0 | 0.0 | 0.0 |

*Inspection of table S16 shows that the PK/PD target is only reached for a MIC of 0.25 mg/L for the French, Swedish and Japanese formulations. On the other hand, the USA1 formulation cannot reach plasma concentrations which exceed 0.25 mg/L, confirming the conclusions obtained by considering fAUC/MIC for USA1. With the usual recommended dosage (22,000 IU/kg or 12.4 mg/kg/day), no formulation reaches the PK/PD target of 30% for a MIC of 0.50 mg/L over 72h*

**Supplementary Table S17:** Cumulative times (h) for which free plasma concentration remained above the MIC for Duplocilline© over the 96 h of a treatment duration as suggested by the SPC.

| **MIC**  **(mg/L)** | **Dose**  **(µg/kg)** | **Interval** | **Nation** | **Mean** | **CV**  **Percent** | **Q95 (h)** | **Q90 (h)** | **Q75 (h)** | **Q50 (h)** |
| --- | --- | --- | --- | --- | --- | --- | --- | --- | --- |
| **0.0625** | 12360 | Twice at 48 h | France | 68.1 | 31.34 | 27.9 | **39.2** | 55.6 | 71.8 |
| 0.1000 | 12360 | Twice at 48 h | France | 45.1 | 43.41 | 6.0 | 18.8 | 33.4 | 46.3 |
| 0.1250 | 12360 | Twice at 48 h | France | 35.4 | 49.71 | 0 | 9.2 | 24.8 | 37.0 |
| 0.2500 | 12360 | Twice at 48 h | France | 12.9 | 89.19 | 0 | 0 | 0 | 12.8 |
| 0.3750 | 12360 | Twice at 48 h | France | 5.1 | 142 | 0 | 0 | 0 | 0 |
| 0.5000 | 12360 | Twice at 48 h | France | 2.0 | 214 | 0 | 0 | 0 | 0 |
| 1.0000 | 12360 | Twice at 48 h | France | 0.0572 | 998 | 0 | 0 | 0 | 0 |
| 2.0000 | 12360 | Twice at 48 h | France | 0 |  | 0 | 0 | 0 | 0 |

*The cumulative time spent above the MIC should reach 30 or 40% of the duration of the treatment, i.e. for the 96 hours supposed to be covered by Duplocilline, cumulative duration of 28.8 or 38.4 hours. Only for MIC of 0.0625 mg/L, is Duplocilline able to achieve such an objective for 90% of horse’s population.*

**Supplementary Table S18:** Cumulative times (h) spent above the MIC for penethamate (Penetavet©) over the 72 h of a treatment duration.

| **MIC** | **Dosage regimen (µg/kg)** | **Nation** | **Mean** | **CV**  **Percent** | **Q95**  **(h)** | **Q90**  **(h)** | **Q75%**  **(h)** | **Q50%**  **(h)** |
| --- | --- | --- | --- | --- | --- | --- | --- | --- |
| 0.0625 | 12360 then 6180 twice at 24h intervals | France | 62.0 | 20.36 | 35.9 | **43.6** | 54.6 | 68.2 |
| 0.1000 | 12361 then 6180 twice at 24h intervals | France | 44.9 | 40.60 | 14.4 | 19.4 | 30.0 | 46.3 |
| 0.1250 | 12362 then 6180 twice at 24h intervals | France | 33.6 | 53.11 | 8.1 | 11.3 | 19.1 | 31.9 |
| 0.2500 | 12363 then 6180 twice at 24h intervals | France | 8.3 | 96.70 | 0 | 0 | 0.79 | 7.2 |
| 0.3750 | 12364 then 6180 twice at 24h intervals | France | 3.2 | 135 | 0 | 0 | 0 | 0 |
| 0.5000 | 12365 then 6180 twice at 24h intervals | France | 1.6 | 175 | 0 | 0 | 0 | 0 |
| 1.0000 | 12366 then 6180 twice at 24h intervals | France | 0.23 | 337 | 0 | 0 | 0 | 0 |
| 2.0000 | 12367 then 6180 twice at 24h intervals | France | 0.029 | 692 | 0 | 0 | 0 | 0 |

*The cumulative time spent above the MIC should reach 30 or 40% of the duration of the treatment, i.e. for the 72 hours supposed to be covered by Penetavet®, cumulative duration of 21.6 or 28.8 hours. Only for a MICs of 0.0625 mg/L, is Penetavet® able to achieve such an objective for 90% of the horse’s population*.

**Supplementary Table S19:** Cumulative times (h) spent above the MIC for BP-Na administered by the IM route (Geepenil ©) over the 72 h of a treatment duration for different dose levels and different dosing intervals for MIC ranging from 0.0625 to 2.0 mg/L. Pharmacodynamic target is 30 or 40% of the dosing interval i.e. a cumulated duration of 21.3 or 28.8 h over 72h.

| **MIC**  **(mg/L)** | **Dose**  **(µg/kg)** | **Interval**  **(h)** | **Mean**  **(h)** | **CV**  **Percent** | **Q95**  **(h)** | **Q90**  **(h)** | **Q75**  **(h)** | **Q50**  **(h)** |
| --- | --- | --- | --- | --- | --- | --- | --- | --- |
| 0.0625 | 12360 | 6 | 72.0 | 0.55 | 72.0 | 72.0 | 72.0 | 72.0 |
| 0.1000 | 12360 | 6 | 71.9 | 0.98 | 72.0 | 72.0 | 72.0 | 72.0 |
| 0.1250 | 12360 | 6 | 71.9 | 1.34 | 71.9 | 72.0 | 72.0 | 72.0 |
| **0.2500** | **12360** | **6** | 71.5 | 3.27 | 70.7 | **71.8** | 72.0 | 72.0 |
| 0.3750 | 12360 | 6 | 70.8 | 5.19 | 62.7 | 69.9 | 71.9 | 72.0 |
| **0.5000** | **12360** | 6 | 69.8 | 7.27 | 56.7 | **62.9** | 71.0 | 71.9 |
| 1.0000 | 12360 | 6 | 57.9 | 21.53 | 33.9 | 40.8 | 50.8 | 60.6 |
| 2.0000 | 12360 | 6 | 26.7 | 57.71 | 4.7 | 6.8 | 12.5 | 27.2 |
| 0.0625 | 12360 | 8 | 71.9 | 1.08 | 72.0 | 72.0 | 72.0 | 72.0 |
| 0.1000 | 12360 | 8 | 71.8 | 1.92 | 71.9 | 72.0 | 72.0 | 72.0 |
| 0.1250 | 12360 | 8 | 71.7 | 2.46 | 71.7 | 72.0 | 72.0 | 72.0 |
| **0.2500** | **12360** | **8** | 70.6 | 5.43 | 61.9 | **67.2** | 71.9 | 72.0 |
| 0.3750 | 12360 | 8 | 68.0 | 8.88 | 54.1 | 58.3 | 65.8 | 71.5 |
| **0.5000** | **12360** | **8** | **63.7** | 12.45 | 48.5 | **51.9** | 58.3 | 65.5 |
| **1.0000** | **12360** | **8** | **43.8** | 25.49 | 24.7 | **31.4** | 37.6 | 44.3 |
| 2.0000 | 12360 | 8 | 18.3 | 67.84 | 0.0 | 2.8 | 6.1 | 18.6 |
| 0.0625 | 12360 | 12 | 70.8 | 7.68 | 64.4 | 72.0 | 72.0 | 72.0 |
| 0.1000 | 12360 | 12 | 68.9 | 11.57 | 51.3 | 62.0 | 70.8 | 72.0 |
| 0.1250 | 12360 | 12 | 66.8 | 14.05 | 45.1 | 56.1 | 65.7 | 71.8 |
| **0.2500** | **12360** | **12** | 53.5 | 21.56 | 28.7 | 37.0 | 48.7 | 55.5 |
| 0.3750 | 12360 | 12 | 44.1 | 25.35 | 21.1 | 27.3 | 38.5 | 45.9 |
| **0.5000** | **12360** | **12** | 37.5 | 28.45 | 16.8 | 21.6 | 31.1 | 39.1 |
| 1.0000 | 12360 | 12 | 22.3 | 39.41 | 8.9 | 10.8 | 15.4 | 22.4 |
| 2.0000 | 12360 | 12 | 9.6 | 62.40 | 0.0 | 2.8 | 5.9 | 8.4 |

*For BP-Na, the objective of 40% of the dosing interval is achieved for a MIC of 0.25 mg/L for all dosing regimens including for a dosing interval of 12 h (i.e. a total daily dose of 24720 µg/kg or 44000 IU per day). For a MIC of 0.5 mg/L, an objective of 30% of the dosing interval is also achieved for all regimens. For an MIC of 1 mg/L, only a dosage regiment of 12360 µg/kg every 6 or 8 h could reach PDT of 40% of the dosing interval in 90% of horses.*

**Supplementary Table S20:** Cumulative times (h) over 24 h spent above the MIC (0.0625 to 2 mg/L) for BP administered by the IV route as a 24 h **continuous infusion** for three dose levels (12360,18540 or 24720 µg/kg in toto). Being an infusion, it was assumed a rather severe condition (e.g. sepsis) and the pharmacodynamic target (PDT) has been set at 90% of the infusion time which means that the selected PK/PD cutoffs (0.25, 0.5 mg/L) must guarantee in 90% of horses, that free plasma BP concentration must be at least equal or higher than 0.25 or 0.5 mg/L over 90% of the infusion time i.e. 21.6 h. Results are given for the three nations having provided rich data for IV administration (France, Sweden and USA2). Their plasma clearances were 481, 243 and 420 mL/kg/h respectively. PDT was fixed to 90% of the infusion duration i.e. 21.6 h.

| **QMIC**  **(mg/L)** | **Dose**  **(µg/kg)** | **Infusion duration (h)** | **Nation** | **Mean**  **(h)** | **CV**  **%** | **Q95**  **(h)** | **Q90**  **(h)** | **Q75**  **(h)** | **Q50**  **(h)** |
| --- | --- | --- | --- | --- | --- | --- | --- | --- | --- |
| 0.0625 | 12360 | 24 | France | 23.9 | 0.53 | 23.7 | 23.8 | 23.9 | 24.0 |
| 0.0625 | 12360 | 24 | Sweden | 23.9 | 0.43 | 23.8 | 23.8 | 23.9 | 24.0 |
| 0.0625 | 12360 | 24 | USA2 | 23.9 | 0.53 | 23.7 | 23.8 | 23.9 | 24.0 |
| 0.0625 | 18840 | 24 | France | 24.0 | 0.33 | 23.8 | 23.9 | 23.9 | 24.0 |
| 0.0625 | 18840 | 24 | Sweden | 24.0 | 0.36 | 23.8 | 23.9 | 24.0 | 24.0 |
| 0.0625 | 18840 | 24 | USA2 | 24.0 | 0.33 | 23.8 | 23.9 | 23.9 | 24.0 |
| 0.0625 | 24720 | 24 | France | 24.0 | 0.25 | 23.9 | 23.9 | 24.0 | 24.0 |
| 0.0625 | 24720 | 24 | Sweden | 24.0 | 0.24 | 23.9 | 23.9 | 24.0 | 24.0 |
| 0.0625 | 24720 | 24 | USA2 | 24.0 | 0.24 | 23.9 | 23.9 | 24.0 | 24.0 |
| 0.1000 | 12360 | 24 | France | 23.9 | 0.96 | 23.5 | 23.7 | 23.9 | 23.9 |
| 0.1000 | 12360 | 24 | Sweden | 23.9 | 0.72 | 23.6 | 23.7 | 23.9 | 23.9 |
| 0.1000 | 12360 | 24 | USA2 | 23.9 | 0.93 | 23.5 | 23.7 | 23.9 | 23.9 |
| 0.1000 | 18840 | 24 | France | 23.9 | 0.57 | 23.7 | 23.8 | 23.9 | 24.0 |
| 0.1000 | 18840 | 24 | Sweden | 23.9 | 0.60 | 23.7 | 23.8 | 23.9 | 24.0 |
| 0.1000 | 18840 | 24 | USA2 | 23.9 | 0.55 | 23.7 | 23.8 | 23.9 | 24.0 |
| 0.1000 | 24720 | 24 | France | 23.9 | 0.42 | 23.8 | 23.9 | 23.9 | 24.0 |
| 0.1000 | 24720 | 24 | Sweden | 23.9 | 0.39 | 23.8 | 23.9 | 23.9 | 24.0 |
| 0.1000 | 24720 | 24 | USA2 | 23.9 | 0.40 | 23.8 | 23.9 | 23.9 | 24.0 |
| 0.1250 | 12360 | 24 | France | 23.8 | 1.31 | 23.4 | 23.6 | 23.8 | 23.9 |
| 0.1250 | 12360 | 24 | Sweden | 23.9 | 0.94 | 23.5 | 23.7 | 23.8 | 23.9 |
| 0.1250 | 12360 | 24 | USA2 | 23.8 | 1.24 | 23.4 | 23.6 | 23.8 | 23.9 |
| 0.1250 | 18840 | 24 | France | 23.9 | 0.74 | 23.6 | 23.7 | 23.9 | 24.0 |
| 0.1250 | 18840 | 24 | Sweden | 23.9 | 0.77 | 23.7 | 23.8 | 23.9 | 24.0 |
| 0.1250 | 18840 | 24 | USA2 | 23.9 | 0.71 | 23.7 | 23.8 | 23.9 | 24.0 |
| 0.1250 | 24720 | 24 | France | 23.9 | 0.54 | 23.7 | 23.8 | 23.9 | 24.0 |
| 0.1250 | 24720 | 24 | Sweden | 23.9 | 0.50 | 23.8 | 23.8 | 23.9 | 24.0 |
| 0.1250 | 24720 | 24 | USA2 | 23.9 | 0.51 | 23.7 | 23.8 | 23.9 | 24.0 |
| **0.2500** | **12360** | 24 | **France** | 23.0 | 15.16 | 21.0 | **22.5** | 23.4 | 23.8 |
| **0.2500** | **12360** | 24 | **Sweden** | 23.7 | 2.36 | 22.8 | **23.2** | 23.6 | 23.8 |
| **0.2500** | **12360** | 24 | **USA2** | 23.5 | 5.30 | 22.3 | **22.9** | 23.5 | 23.8 |
| 0.2500 | 18840 | 24 | France | 23.7 | 2.55 | 23.0 | 23.3 | 23.7 | 23.9 |
| 0.2500 | 18840 | 24 | Sweden | 23.8 | 1.77 | 23.3 | 23.5 | 23.8 | 23.9 |
| 0.2500 | 18840 | 24 | USA2 | 23.8 | 1.75 | 23.2 | 23.5 | 23.7 | 23.9 |
| 0.2500 | 24720 | 24 | France | 23.8 | 1.33 | 23.4 | 23.6 | 23.8 | 23.9 |
| 0.2500 | 24720 | 24 | Sweden | 23.9 | 1.10 | 23.5 | 23.7 | 23.8 | 23.9 |
| 0.2500 | 24720 | 24 | USA2 | 23.8 | 1.17 | 23.4 | 23.6 | 23.8 | 23.9 |
| 0.3750 | 12360 | 24 | France | 15.9 | 67.01 | 0.0 | 0.0 | 0.0 | 23.0 |
| 0.3750 | 12360 | 24 | Sweden | 23.3 | 5.79 | 21.6 | 22.5 | 23.3 | 23.7 |
| 0.3750 | 12360 | 24 | USA2 | 21.6 | 27.26 | 0.0 | 20.0 | 22.7 | 23.5 |
| 0.3750 | 18840 | 24 | France | 23.0 | 15.06 | 21.0 | 22.4 | 23.4 | 23.8 |
| 0.3750 | 18840 | 24 | Sweden | 23.6 | 3.28 | 22.7 | 23.2 | 23.6 | 23.8 |
| 0.3750 | 18840 | 24 | USA2 | 23.5 | 4.69 | 22.4 | 23.0 | 23.5 | 23.8 |
| 0.3750 | 24720 | 24 | France | 23.7 | 3.33 | 22.8 | 23.2 | 23.7 | 23.9 |
| 0.3750 | 24720 | 24 | Sweden | 23.8 | 1.83 | 23.1 | 23.4 | 23.7 | 23.9 |
| 0.3750 | 24720 | 24 | USA2 | 23.7 | 2.11 | 23.0 | 23.4 | 23.7 | 23.9 |
| 0.5000 | 12360 | 24 | France | 6.1 | 166.18 | 0.0 | 0.0 | 0.0 | 0.0 |
| 0.5000 | 12360 | 24 | Sweden | 22.5 | 15.85 | 18.8 | 21.1 | 22.7 | 23.4 |
| 0.5000 | 12360 | 24 | USA2 | 15.2 | 70.73 | 0.0 | 0.0 | 0.0 | 22.3 |
| 0.5000 | 18840 | 24 | France | 19.1 | 46.66 | 0.0 | 0.0 | 21.9 | 23.4 |
| **0.5000** | **18840** | 24 | Sweden | 23.4 | 5.32 | 22.0 | **22.7** | 23.4 | 23.7 |
| **0.5000** | **18840** | 24 | USA2 | 22.7 | 16.05 | 20.1 | **22.0** | 23.1 | 23.7 |
| **0.5000** | **24720** | 24 | France | 23.1 | 13.63 | 21.4 | **22.5** | 23.4 | 23.8 |
| 0.5000 | 24720 | 24 | Sweden | 23.6 | 2.75 | 22.7 | 23.2 | 23.6 | 23.8 |
| 0.5000 | 24720 | 24 | USA2 | 23.5 | 3.91 | 22.3 | 23.0 | 23.5 | 23.8 |
| 1.0000 | 12360 | 24 | France | 0.0 | 3231.07 | 0.0 | 0.0 | 0.0 | 0.0 |
| 1.0000 | 12360 | 24 | Sweden | 5.8 | 167.34 | 0.0 | 0.0 | 0.0 | 0.0 |
| 1.0000 | 12360 | 24 | USA2 | 0.3 | 835.84 | 0.0 | 0.0 | 0.0 | 0.0 |
| 1.0000 | 18840 | 24 | France | 1.1 | 451.68 | 0.0 | 0.0 | 0.0 | 0.0 |
| 1.0000 | 18840 | 24 | Sweden | 18.3 | 47.59 | 0.0 | 0.0 | 19.6 | 22.7 |
| 1.0000 | 18840 | 24 | USA2 | 5.8 | 169.71 | 0.0 | 0.0 | 0.0 | 0.0 |
| 1.0000 | 24720 | 24 | France | 6.3 | 163.18 | 0.0 | 0.0 | 0.0 | 0.0 |
| 1.0000 | 24720 | 24 | Sweden | 22.4 | 16.63 | 18.2 | 20.9 | 22.7 | 23.5 |
| 1.0000 | 24720 | 24 | USA2 | 15.6 | 68.56 | 0.0 | 0.0 | 0.0 | 22.6 |
| 2.0000 | 12360 | 24 | France | 0.0 |  | 0.0 | 0.0 | 0.0 | 0.0 |
| 2.0000 | 12360 | 24 | Sweden | 0.0 |  | 0.0 | 0.0 | 0.0 | 0.0 |
| 2.0000 | 12360 | 24 | USA2 | 0.0 |  | 0.0 | 0.0 | 0.0 | 0.0 |
| 2.0000 | 18840 | 24 | France | 0.0 |  | 0.0 | 0.0 | 0.0 | 0.0 |
| 2.0000 | 18840 | 24 | Sweden | 0.8 | 523.05 | 0.0 | 0.0 | 0.0 | 0.0 |
| 2.0000 | 18840 | 24 | USA2 | 0.0 | 3574.40 | 0.0 | 0.0 | 0.0 | 0.0 |
| 2.0000 | 24720 | 24 | France | 0.0 | 4085.63 | 0.0 | 0.0 | 0.0 | 0.0 |
| 2.0000 | 24720 | 24 | Sweden | 5.9 | 165.33 | 0.0 | 0.0 | 0.0 | 0.0 |
| 2.0000 | 24720 | 24 | USA2 | 0.3 | 793.55 | 0.0 | 0.0 | 0.0 | 0.0 |

*For a MIC of 0.25 mg/L, 24 h infusions of a total dose of 12.4 mg/kg (22,000 IU/kg) were able to maintain BP concentrations for more than 22 h out of the 24 h infusion for 90% of the horses and this, for the three nations. On the other hand, for a MIC of 0.50 mg/L, only the Swedish horses were able to be above the MIC for 21 hours. To be above the MIC of 0.5 mg/L, a total dose of 18840 µg/kg (USA2) or 24720 µg/kg (France) was required. These differences reflect those of plasma clearances.*

**Supplementary Table S21**: Cumulative times (h) over 24 h spent above the MIC (0.0625 to 2 mg/L) for BP administered by the **IV route as single boluses** (short infusion of 0.083 h) for three dose levels (12360,18540 or 24720 µg/kg in toto). Results are given for the three nations having provided rich data for IV administration (France, Sweden and USA2). Their plasma clearances were 481, 243 and 420 mL/kg/h respectively. Target is 30 or 40% of the dosing interval i.e. a cumulated duration of 2.4 or 3.2 h over 8h assuming that the prescriber anticipates repeating this administration in the form of a bolus 3 times per 24 hours.

| **MIC (mg/L)** | **Dose (µg/kg)** | **Infusion duration**  **(h)** | **Nation** | **Mean (h)** | **CV**  **%** | **Q95 (h)** | **Q90 (h)** | **Q75 (h)** | **Q50 (h)** |
| --- | --- | --- | --- | --- | --- | --- | --- | --- | --- |
| 0.0625 | 12360 | 0.0830 | France | 4.37 | 35.54 | 2.74 | 2.98 | 3.44 | 4.04 |
| 0.0625 | 12360 | 0.0830 | Sweden | 8.41 | 35.34 | 5.21 | 5.65 | 6.57 | 7.75 |
| 0.0625 | 12360 | 0.0830 | USA2 | 5.71 | 35.25 | 3.58 | 3.89 | 4.48 | 5.27 |
| 0.0625 | 18840 | 0.0830 | France | 5.11 | 33.42 | 3.26 | 3.52 | 4.03 | 4.75 |
| 0.0625 | 18840 | 0.0830 | Sweden | 9.44 | 34.27 | 5.80 | 6.33 | 7.39 | 8.74 |
| 0.0625 | 18840 | 0.0830 | USA2 | 6.63 | 35.20 | 4.22 | 4.58 | 5.24 | 6.13 |
| 0.0625 | 24720 | 0.0830 | France | 5.62 | 30.69 | 3.70 | 4.00 | 4.53 | 5.30 |
| 0.0625 | 24720 | 0.0830 | Sweden | 10.11 | 33.01 | 6.27 | 6.80 | 7.91 | 9.43 |
| 0.0625 | 24720 | 0.0830 | USA2 | 7.23 | 33.03 | 4.62 | 5.04 | 5.78 | 6.79 |
| **0.1000** | **12360** | 0.0830 | **France** | 3.64 | 37.93 | 2.20 | **2.40** | 2.80 | 3.33 |
| 0.1000 | 12360 | 0.0830 | Sweden | 7.26 | 37.09 | 4.44 | 4.81 | 5.58 | 6.63 |
| 0.1000 | 12360 | 0.0830 | USA2 | 4.81 | 37.50 | 2.92 | 3.19 | 3.70 | 4.40 |
| 0.1000 | 18840 | 0.0830 | France | 4.28 | 36.08 | 2.65 | 2.87 | 3.31 | 3.93 |
| 0.1000 | 18840 | 0.0830 | Sweden | 8.27 | 36.11 | 5.06 | 5.52 | 6.41 | 7.57 |
| 0.1000 | 18840 | 0.0830 | USA2 | 5.65 | 37.77 | 3.51 | 3.83 | 4.38 | 5.16 |
| 0.1000 | 24720 | 0.0830 | France | 4.74 | 33.09 | 3.04 | 3.31 | 3.76 | 4.41 |
| 0.1000 | 24720 | 0.0830 | Sweden | 8.93 | 34.69 | 5.52 | 6.00 | 6.93 | 8.26 |
| 0.1000 | 24720 | 0.0830 | USA2 | 6.21 | 35.27 | 3.92 | 4.27 | 4.90 | 5.74 |
| 0.1250 | 12360 | 0.0830 | France | 3.33 | 38.87 | 1.98 | 2.17 | 2.52 | 3.04 |
| 0.1250 | 12360 | 0.0830 | Sweden | 6.74 | 37.92 | 4.10 | 4.44 | 5.13 | 6.13 |
| 0.1250 | 12360 | 0.0830 | USA2 | 4.42 | 38.44 | 2.64 | 2.88 | 3.37 | 4.02 |
| 0.1250 | 18840 | 0.0830 | France | 3.93 | 37.30 | 2.40 | 2.59 | 3.00 | 3.59 |
| 0.1250 | 18840 | 0.0830 | Sweden | 7.72 | 36.99 | 4.71 | 5.13 | 5.95 | 7.04 |
| 0.1250 | 18840 | 0.0830 | USA2 | 5.21 | 38.96 | 3.20 | 3.48 | 4.00 | 4.74 |
| 0.1250 | 24720 | 0.0830 | France | 4.35 | 34.33 | 2.74 | 3.00 | 3.42 | 4.03 |
| 0.1250 | 24720 | 0.0830 | Sweden | 8.38 | 35.60 | 5.16 | 5.61 | 6.47 | 7.72 |
| 0.1250 | 24720 | 0.0830 | USA2 | 5.75 | 36.40 | 3.59 | 3.91 | 4.52 | 5.29 |
| 0.2500 | 12360 | 0.0830 | France | 2.52 | 40.55 | 1.44 | 1.58 | 1.86 | 2.27 |
| **0.2500** | **12360** | 0.0830 | **Sweden** | 5.22 | 39.43 | 3.06 | **3.34** | 3.88 | 4.72 |
| **0.2500** | **12360** | 0.0830 | **USA2** | 3.37 | 40.39 | 1.93 | **2.11** | 2.49 | 3.02 |
| 0.2500 | 18840 | 0.0830 | France | 3.00 | 40.21 | 1.75 | 1.90 | 2.22 | 2.68 |
| 0.2500 | 18840 | 0.0830 | Sweden | 6.10 | 39.31 | 3.62 | 3.94 | 4.59 | 5.49 |
| 0.2500 | 18840 | 0.0830 | USA2 | 4.00 | 41.25 | 2.34 | 2.57 | 2.98 | 3.58 |
| 0.2500 | 24720 | 0.0830 | France | 3.33 | 37.85 | 1.97 | 2.17 | 2.53 | 3.05 |
| 0.2500 | 24720 | 0.0830 | Sweden | 6.72 | 38.59 | 4.04 | 4.42 | 5.10 | 6.12 |
| 0.2500 | 24720 | 0.0830 | USA2 | 4.45 | 39.28 | 2.67 | 2.91 | 3.40 | 4.04 |
| 0.3750 | 12360 | 0.0830 | France | 2.12 | 40.70 | 1.18 | 1.30 | 1.55 | 1.91 |
| 0.3750 | 12360 | 0.0830 | Sweden | 4.43 | 39.10 | 2.55 | 2.78 | 3.25 | 4.01 |
| 0.3750 | 12360 | 0.0830 | USA2 | 2.84 | 40.57 | 1.60 | 1.75 | 2.07 | 2.54 |
| 0.3750 | 18840 | 0.0830 | France | 2.55 | 41.01 | 1.45 | 1.58 | 1.86 | 2.26 |
| 0.3750 | 18840 | 0.0830 | Sweden | 5.24 | 39.87 | 3.04 | 3.32 | 3.88 | 4.69 |
| 0.3750 | 18840 | 0.0830 | USA2 | 3.40 | 41.63 | 1.94 | 2.14 | 2.49 | 3.03 |
| 0.3750 | 24720 | 0.0830 | France | 2.83 | 39.27 | 1.64 | 1.80 | 2.12 | 2.57 |
| 0.3750 | 24720 | 0.0830 | Sweden | 5.82 | 39.79 | 3.43 | 3.76 | 4.35 | 5.24 |
| 0.3750 | 24720 | 0.0830 | USA2 | 3.80 | 40.25 | 2.21 | 2.43 | 2.85 | 3.43 |
| 0.5000 | 12360 | 0.0830 | France | 1.86 | 40.57 | 1.02 | 1.13 | 1.35 | 1.68 |
| 0.5000 | 12360 | 0.0830 | Sweden | 3.91 | 38.42 | 2.22 | 2.44 | 2.86 | 3.54 |
| 0.5000 | 12360 | 0.0830 | USA2 | 2.50 | 40.25 | 1.39 | 1.53 | 1.82 | 2.24 |
| 0.5000 | 18840 | 0.0830 | France | 2.26 | 41.17 | 1.26 | 1.38 | 1.63 | 2.00 |
| 0.5000 | 18840 | 0.0830 | Sweden | 4.67 | 39.91 | 2.67 | 2.91 | 3.43 | 4.18 |
| 0.5000 | 18840 | 0.0830 | USA2 | 3.02 | 41.35 | 1.70 | 1.87 | 2.19 | 2.69 |
| 0.5000 | 24720 | 0.0830 | France | 2.52 | 39.89 | 1.44 | 1.58 | 1.87 | 2.27 |
| 0.5000 | 24720 | 0.0830 | Sweden | 5.21 | 40.06 | 3.03 | 3.31 | 3.87 | 4.68 |
| 0.5000 | 24720 | 0.0830 | USA2 | 3.38 | 40.53 | 1.94 | 2.13 | 2.51 | 3.04 |
| 1.0000 | 12360 | 0.0830 | France | 1.32 | 39.64 | 0.66 | 0.77 | 0.95 | 1.21 |
| 1.0000 | 12360 | 0.0830 | Sweden | 2.80 | 35.71 | 1.58 | 1.75 | 2.07 | 2.59 |
| 1.0000 | 12360 | 0.0830 | USA2 | 1.78 | 38.36 | 0.95 | 1.07 | 1.29 | 1.63 |
| 1.0000 | 18840 | 0.0830 | France | 1.64 | 40.53 | 0.87 | 0.98 | 1.18 | 1.48 |
| 1.0000 | 18840 | 0.0830 | Sweden | 3.45 | 38.38 | 1.91 | 2.12 | 2.52 | 3.13 |
| 1.0000 | 18840 | 0.0830 | USA2 | 2.20 | 39.35 | 1.21 | 1.34 | 1.59 | 1.99 |
| 1.0000 | 24720 | 0.0830 | France | 1.87 | 40.15 | 1.02 | 1.13 | 1.35 | 1.67 |
| 1.0000 | 24720 | 0.0830 | Sweden | 3.91 | 39.16 | 2.21 | 2.42 | 2.86 | 3.52 |
| 1.0000 | 24720 | 0.0830 | USA2 | 2.51 | 39.76 | 1.40 | 1.54 | 1.82 | 2.27 |
| 2.0000 | 12360 | 0.0830 | France | 0.87 | 37.59 | 0.40 | 0.49 | 0.64 | 0.83 |
| 2.0000 | 12360 | 0.0830 | Sweden | 1.87 | 32.55 | 1.04 | 1.18 | 1.42 | 1.79 |
| 2.0000 | 12360 | 0.0830 | USA2 | 1.18 | 35.97 | 0.58 | 0.70 | 0.88 | 1.13 |
| 2.0000 | 18840 | 0.0830 | France | 1.13 | 38.92 | 0.53 | 0.65 | 0.82 | 1.05 |
| 2.0000 | 18840 | 0.0830 | Sweden | 2.41 | 34.34 | 1.35 | 1.49 | 1.80 | 2.25 |
| 2.0000 | 18840 | 0.0830 | USA2 | 1.52 | 36.94 | 0.79 | 0.91 | 1.12 | 1.42 |
| 2.0000 | 24720 | 0.0830 | France | 1.32 | 39.10 | 0.67 | 0.77 | 0.95 | 1.21 |
| 2.0000 | 24720 | 0.0830 | Sweden | 2.80 | 36.01 | 1.58 | 1.73 | 2.08 | 2.57 |
| 2.0000 | 24720 | 0.0830 | USA2 | 1.78 | 37.78 | 0.96 | 1.08 | 1.31 | 1.65 |

*For the single IV bolus and the three doses tested, only Swedish and USA2 horses were able to achieve a PDT of 30% over 8 h for a MIC of 0.25 mg/L.*
